# Bridging energy and proteome allocation models allows prediction of the resource cost of traits across environments

**DOI:** 10.1371/journal.pbio.3003995

**Published:** 2026-10-13

**Authors:** Mohammadjavad Meghrazi, Sarah P. Otto

**Affiliations:** Department of Zoology and Biodiversity Research Center, The University of British Columbia, Vancouver, BC, Canada; Universität Bern: Universitat Bern, SWITZERLAND

## Abstract

Many traits divert resources from reproduction and are costly due to the physiological trade-offs organisms face. These traits might require the allocation of different resources, e.g., energy expenditure or production of macromolecules, and it is unclear how their costs can be compared given their different units. Moreover, the effect of environmental conditions and genetic background on the cost of traits is not well understood. Two different frameworks have been proposed to quantify the resource costs of traits, focusing on either energy allocation or proteome allocation. These frameworks implicitly assume energy provisioning or protein production limits growth, respectively, but the connection between the two limitations has been underexplored. To better connect these frameworks, we develop a framework that considers limitations in energy provisioning and macromolecule biosynthesis simultaneously to quantify and compare the cost of traits that require different resources. Using this model, we explore how environmental quality and genetic background affect the cost of traits. Notably, our model predicts that increasing food quality increases the cost of traits that require the production of new structures, while decreasing the cost of traits requiring energy expenditure. Moreover, our model makes novel predictions about the effect of degradation, recycling, and energetic demands for maintenance on the relationship between the macromolecular composition of the cells and growth rate (also known as bacterial growth laws).

## Abstract 2

Physiological trade-offs make traits costly, yet comparing costs across traits that require different resources remains challenging. This study shows that a unified energy-proteome allocation framework predicts how environmental conditions shape trait costs and growth.

## Introduction

To understand how evolution shapes organismal traits, it is necessary to consider both positive and negative fitness consequences of traits. While positive effects can result from improving a wide range of functions, one of the main sources of negative impacts is the resource cost of traits [1,2]. Due to physiological trade-offs, the diversion of resources to trait construction can reduce the reproduction rate, and this reduction can be defined as the resource cost of traits (hereafter, cost of traits) [2].

Various traits may require the allocation of different resources, making it challenging to convert their costs to a reduction in reproduction rate using a unified framework. For example, resistance to antibiotics in bacteria could be achieved by the production of new proteins (e.g., proteins that degrade the antibiotics) or energy expenditure (e.g., faster activity of pumps that remove antibiotics from the cytoplasm) [3]. Both of these traits are costly, but it is unclear how their costs, i.e., protein production and energy expenditure, can be compared given their different units. Moreover, the cost depends on the environment (e.g., temperature) and genetic background (e.g., temperature tolerance) of the cells in a manner that is not well understood [4]. As less costly traits are more likely to evolve, if we can convert the costs of different traits to a common unit and compare them, we can predict which traits are more likely to evolve, all else being equal. Moreover, understanding how the environment and genetic background affect the cost of traits will help us predict the conditions that facilitate the evolution of various traits, including resistance to stressors (e.g., antibiotics, extreme temperatures, and predation). This can have important implications for the management of the antimicrobial resistance crisis and, more broadly, understanding the evolutionary response of organisms to the increased prevalence of human-induced stressors.

Energy allocation and proteome allocation are the two main frameworks used to study the cost of traits, however, they have different underlying assumptions, and the connection between them is underexplored [5]. According to energy allocation models, the cell divides its limited energy budget between maintenance, growth, and different activities. The traits that directly or indirectly (i.e., by using metabolites) consume energy reduce energy allocation to growth and are thus costly. In this framework, the cost of a trait is thus translated into energy requirements and depends on the fraction of the cell’s energy budget consumed [2,5–7]. This is equivalent to assuming cells are energy-limited, i.e., they cannot increase their energy budget [5], and thus omitting the limitations imposed by the catalytic capacity of enzymes that convert metabolites and energy into biomass, i.e., biosynthetic limitations [8–10]. On the other hand, the proteome allocation framework (also known as ribosomal allocation) considers protein production to be the limiting process in cell reproduction, emphasizing limitations in translation. From this perspective, allocating proteins to a trait decreases the allocation to the protein production machinery, which increases the time required to reproduce the proteome of a cell [9–14]. While proteome allocation models investigate the connections between nutritional and translational limitations [9,10,15,16], their scope remains limited to proteins, and it is unclear how the cost of traits that require other macromolecules or energy can be modeled in this framework. Overall, energy provisioning and translation are specific cases of processing nutrients (provisioning) and converting them to macromolecules (biosynthesis), respectively (Fig 2). As there is ample evidence for limitations in both provisioning and biosynthesis [6,8,9,17], the unification of these two frameworks is necessary for a better understanding of the cost of traits and the constraints under which cells grow and reproduce.

We develop a macromolecule allocation framework that considers provisioning and biosynthesis simultaneously to quantify how each limits cell growth. Using this model, we quantify the cost of allocating different resources to traits and the effect of environmental conditions and genetic background on this cost. Notably, we show that the cost of traits that require macromolecule production is higher in environments favorable for growth. However, the cost of traits that require energy expenditure or a higher degradation rate of harmful proteins depends on the extent to which provisioning and biosynthesis limit growth, which in turn depends on genotype and environment. Factors that increase the speed of provisioning and make it less limiting (e.g., higher food quality) decrease the cost of energy expenditure and increase the cost of degradation, while factors that increase the speed of biosynthesis (e.g., faster ribosomes) have the opposite effect. Moreover, our model makes novel predictions about the effect of degradation, recycling, and energetic demands for maintenance on the relationship between the fraction of ribosomes in the proteome and growth rate (also known as bacterial growth laws; explained in Box 1), particularly during slow growth.

**Box 1. Bacterial growth laws**

The macromolecular composition of single-celled organisms is tightly correlated with their growth rate at steady state [18]. When food quality is altered, a positive linear relationship is observed between the growth rate and the fraction of ribosomes in the proteome, which is often measured approximately using the ratio of RNA to proteins (Fig 1a) [10]. However, when ribosomes are partially inhibited, the relationship between growth rate and the ribosomal fraction switches to a negative one [10]. Both of these relationships between the fraction of ribosomes and growth rate are robust and linear, except at slow growth, where the ribosomal fraction is higher than expected based on the linear relationship (concave up) [19]. These patterns are called bacterial growth laws [10] (Fig 1a). More recently, these trends have been confirmed by directly measuring the fraction of ribosomes in the proteome [17,20–24].

**Fig 1:**
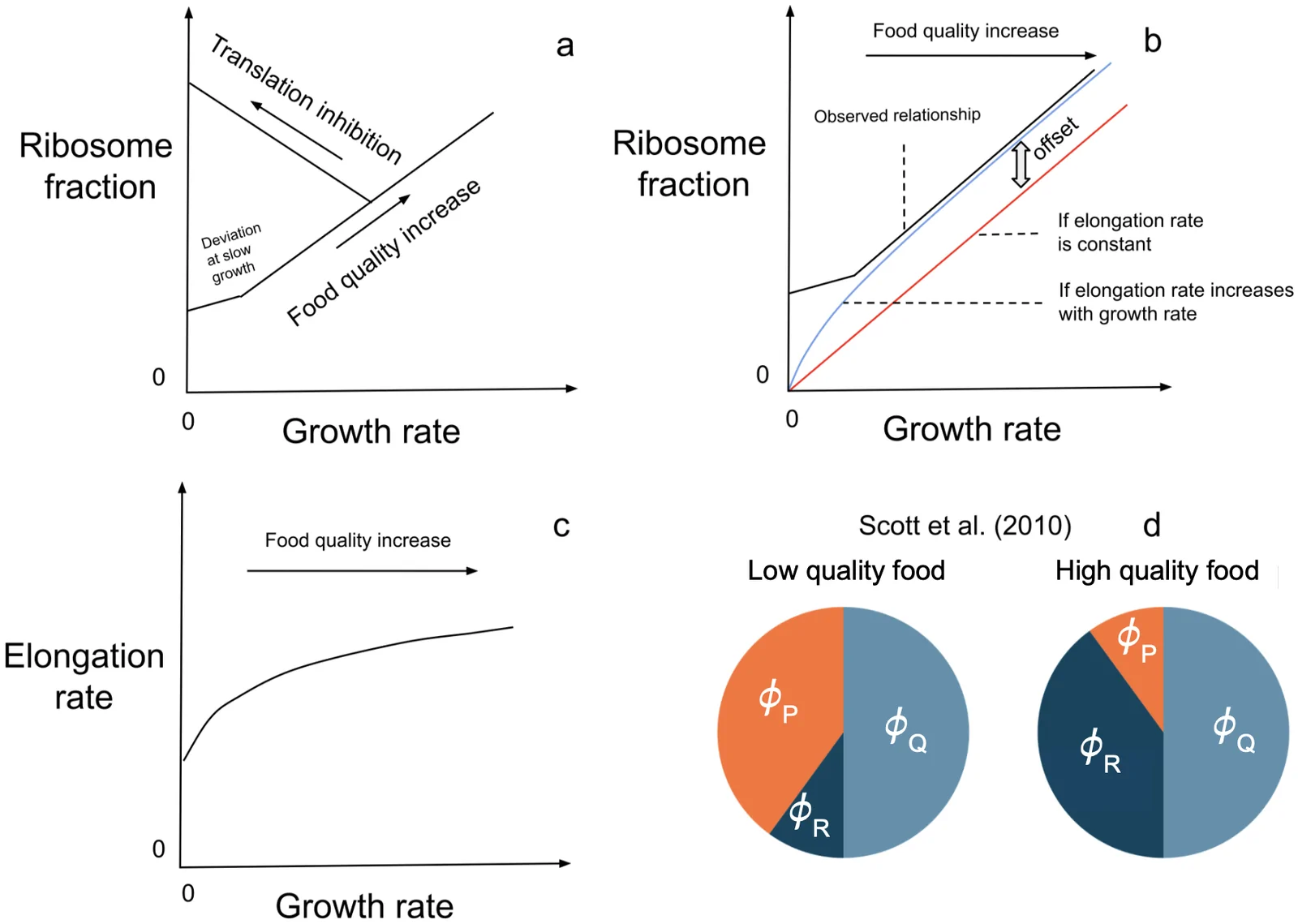
Bacterial growth laws. Bacterial growth laws describe the relationship between growth rate and the fraction of ribosomes in the proteome when altering food quality or inhibiting translation (panel **a**). When altering food quality, this relationship has a positive y-intercept (panel **b**, black line), which is not expected if ribosomes were always working with a constant elongation rate (red line) [23]. On the other hand, if the elongation rate increases with growth rate, a decelerating relationship between growth rate and ribosomal fraction is expected (blue line), which is not observed [14]. Nevertheless, the elongation rate is positively correlated with food quality and approaches half of its maximum value as the growth rate of cells approaches zero (panel **c**) [19]. To explain the bacterial growth laws, Scott and colleagues (2010) used a three-compartment model of the proteome (panel **d**). In their model, when food quality is low, the cell increases allocation to the metabolic proteins that supply ribosomes ($\phi_{P}$). On the other hand, when ribosomes are inhibited, the cell increases the ribosomal fraction ($\phi_{R}$) to compensate for the inhibition. The fixed fraction ($\phi_{Q}$) is composed of proteins with constant relative abundance.

Scott and colleagues (2010) used a three-compartment model of the proteome to account for bacterial growth laws [10]. In this model, ribosomes and other proteins involved in translation are called the R fraction ($\phi_{R}$), metabolic enzymes that provide energy and substrates for ribosomes are called the P fraction ($\phi_{P}$), and the remaining proteins (mainly structural and maintenance proteins), which are assumed to have a fixed relative abundance, are called the fixed fraction ($\phi_{Q}$). The cell can adjust the ratio of R and P fractions to maximize protein production rate, which they assumed is tightly linked to growth rate. Therefore, when the cell is placed in low-quality food, which requires more enzymes to be processed, the P fraction is expected to increase. In the presence of ribosome inhibitors, however, the R fraction must rise to compensate for the inhibition (Fig 1d).

From the perspective of Scott and colleagues (2010), the growth rate equals the ribosomal fraction times the protein-production rate per ribosome mass (which is proportional to the elongation rate). Notice that as the death rate is negligible during exponential growth [25], they consider the growth rate and birth rate as equivalent. To explain the linear relationship between the ribosomal fraction and growth rate when altering food quality, ribosomes are assumed to work at a constant elongation rate, regardless of the conditions within the cell [10]. Otherwise, if the elongation rate covaries with the ribosomal fraction, the relationship between the ribosomal fraction and growth rate would not be linear (Fig 1b, blue line). Nevertheless, it is known that the elongation rate is positively correlated with food quality and approaches half of its maximal value as the growth rate approaches zero (Fig 1c) [19,26–28]. Moreover, if the elongation rate was always constant, then the ribosomal fraction is expected to decline to zero as the growth rate approaches zero, which is not observed (Fig 1b, red line) [23].

To explain these discrepancies, previous studies have investigated the role of inactive ribosomes, degradation, and variable elongation rate [14,19,23,29–31]. To our knowledge, none of the previous studies have investigated the effects of the recycling of degraded macromolecules and energetic demands for maintenance (hereafter maintenance demands), which are particularly important during slow growth [6].

## Model

To quantify the rate of reproduction (i.e., biomass production), we develop a three-compartment model similar to Scott and colleagues (2010) (described in Box 1) but with three differences [10]. First, we include all macromolecules in our model rather than just proteins. Second, the speed of the biosynthesis machinery is allowed to vary. Third, we incorporate degradation, recycling, and energetic demands for maintenance.

We divide cell macromolecules into three fractions based on their metabolic role. The macromolecules that uptake food and nutrients and convert them into substrates (i.e., energy stored in different forms, and building blocks of macromolecules such as amino acids, nucleotides, etc.) make up the provisioning fraction ($\phi_{P}$). The macromolecules that synthesize new macromolecules using substrates comprise the biosynthesis fraction ($\phi_{B}$). The remaining macromolecules are grouped into the structural fraction ($\phi_{S}$) (Fig 2). While these fractions are structurally similar to the ones defined by Scott and colleagues (2010), we broaden the scope of macromolecules considered in the model beyond proteins to capture cell contents more completely. See more details on the components of each fraction in Fig 2 caption and S1 Appendix.

We assume that the structural fraction is fixed, but the cell can optimize the ratio allocated to provisioning versus biosynthesis to maximize biomass production. Previous studies have suggested molecular mechanisms for top-down regulation of proteome allocation between nutritional and biosynthetic pathways [32,33], which motivates our analysis of the impacts on growth rate.

Three processes affect the flux of substrates supplied to the substrate pool. (1) The provisioning fraction supplies substrates proportional to its mass at the rate $M_{P}\;\epsilon_{P}$, where $M_{P}$ is the mass of the provisioning machinery and $\epsilon_{P}$ (provisioning efficiency) is the substrate supply rate per unit mass of the provisioning machinery. (2) Maintenance demands consume substrates (mainly in the form of energy) at a constant rate equal to $M_{Total}\;S_{M}$, where $S_{M}$ is the substrate demand for maintenance per unit mass of cell macromolecules and $M_{Total}$ is the total mass of cell macromolecules. (3) Recycling of degraded macromolecules supplies substrates proportional to the degradation rate and the efficiency of substrate recycling at the rate $M_{Total}\;c\;D$, where *D* is the degradation rate per unit of macromolecule mass and *c* (recycling efficiency) is the fraction of substrates recycled after degradation. Overall, substrates are supplied to the substrate pool at the rate $M_{Total}(\phi_{P}\;\epsilon_{P}-S_{M}+c\;D)$, where $\phi_{P}=M_{P}/M_{Total}$. The biosynthesis fraction consumes substrates proportional to the mass and speed of biosynthesis machinery at the rate $M_{B}\;\epsilon_{B}\;\gamma_{B}(\phi_{B})$, where $M_{B}$ is the mass of biosynthesis machinery, $\epsilon_{B}$ (biosynthesis efficiency) is the rate of substrate consumption per unit mass of biosynthetic machinery when saturated, and $\gamma_{B}(\phi_{B})$ is the biosynthesis saturation level, which depends on the steady-state substrate concentration. As the steady-state substrate concentration depends on the biosynthesis fraction, we model the biosynthesis saturation level directly as a function of the biosynthesis fraction rather than the steady-state substrate concentration. Lastly, substrates are allocated to new cells in proportion to the birth rate and the steady-state substrate concentration (dilution due to growth, see S1 Appendix for more details). Table 1 summarizes the notation.

**Table 1:** List of variables and their units.

Variable symbol	Description	Unit
$M_{X}$	Mass of macromolecules in fraction *X* (*X* = *P*, *B*, or *S*)	g
$M_{Total}$	Total mass of cell macromolecules	g
$\phi_{X}$	The size of fraction X, which equals $\frac{M_{X}}{M_{Total}}$ (*X* = *P*, *B*, or *S*)	dimensionless
$\epsilon_{P}$	Provisioning efficiency: mass of substrates produced by provisioning machinery, per unit mass of provisioning machinery, per unit time	*h* ^–1^
$\epsilon_{B}$	Biosynthesis efficiency: mass of substrates consumed (or macromolecules produced) by biosynthetic machinery when saturated, per unit mass of biosynthetic machinery, per unit time	*h* ^–1^
$\gamma_{B}(\phi_{B})$	The saturation level of biosynthesis which is a function of biosynthesis fraction.	dimensionless
*b*	Birth rate: the mass of macromolecules produced per unit mass of macromolecules, per unit time	*h* ^–1^
*D*	Degradation rate: mass of macromolecules degraded, per unit mass of macromolecules, per unit time	*h* ^–1^
$S_{M}$	Maintenance demands: mass of substrate consumed, per unit mass of macromolecules, per unit time	*h* ^–1^
α	The maximum substrate supply rate (when the provisioning fraction is maximal), which equals $\epsilon_{P}(1-\phi_{S})-S_{M}+c\;D$	*h* ^–1^
*A*	The extent to which provisioning is limiting, which equals $\frac{\epsilon_{B}}{(\sqrt{\epsilon_{B}}+\sqrt{\epsilon_{P}}{)}^{2}}$. When provisioning is the limiting step, *A* approaches 1, and when biosynthesis is limiting, *A* approaches 0.	dimensionless
*R*	The return on investment, which equals $\frac{\epsilon_{P}\;\epsilon_{B}}{(\sqrt{\epsilon_{B}}+\sqrt{\epsilon_{P}}{)}^{2}}$. *R* equals the birth rate achieved in the absence of degradation and maintenance demands if all macromolecules were allocated to provisioning and biosynthesis ($\phi_{S}=0$). Notice that $\frac{1}{\sqrt{R}}=\frac{1}{\sqrt{\epsilon_{B}}}+\frac{1}{\sqrt{\epsilon_{P}}}$.	*h* ^–1^

We assume the macromolecule production rate determines the rate of cell reproduction. Therefore, the birth rate (*b*) equals the net macromolecule production rate per unit of macromolecule mass, which equals the biosynthesis rate minus macromolecule degradation rate divided by the mass of all macromolecules. Assuming biosynthesis rate equals the rate of substrate consumption by the biosynthesis fraction (i.e., no mass lost during biosynthesis), the birth rate equals:


$$\begin{array}{c} b=\frac{1}{M_{Total}}\;\frac{dM_{Total}}{dt}=\frac{M_{B}\;\epsilon_{B}\;\gamma_{B}(\phi_{B})-M_{Total}\;D}{M_{Total}}=\phi_{B}\;\epsilon_{B}\;\gamma_{B}(\phi_{B})-D \end{array}$$ (1)

Assuming the degradation rate is independent of macromolecular allocation, the allocation that maximizes $\phi_{B}\;\epsilon_{B}\;\gamma_{B}(\phi_{B})$ will maximize the birth rate. However, there is a trade-off between the biosynthesis fraction ($\phi_{B}$) and biosynthesis saturation level ($\gamma_{B}(\phi_{B})$). As the biosynthesis fraction increases, there are fewer provisioning enzymes to supply each biosynthetic enzyme, and the biosynthesis saturation level decreases at an accelerating rate. Fig 3a shows a graphical representation of this trade-off.

**Fig 2:**
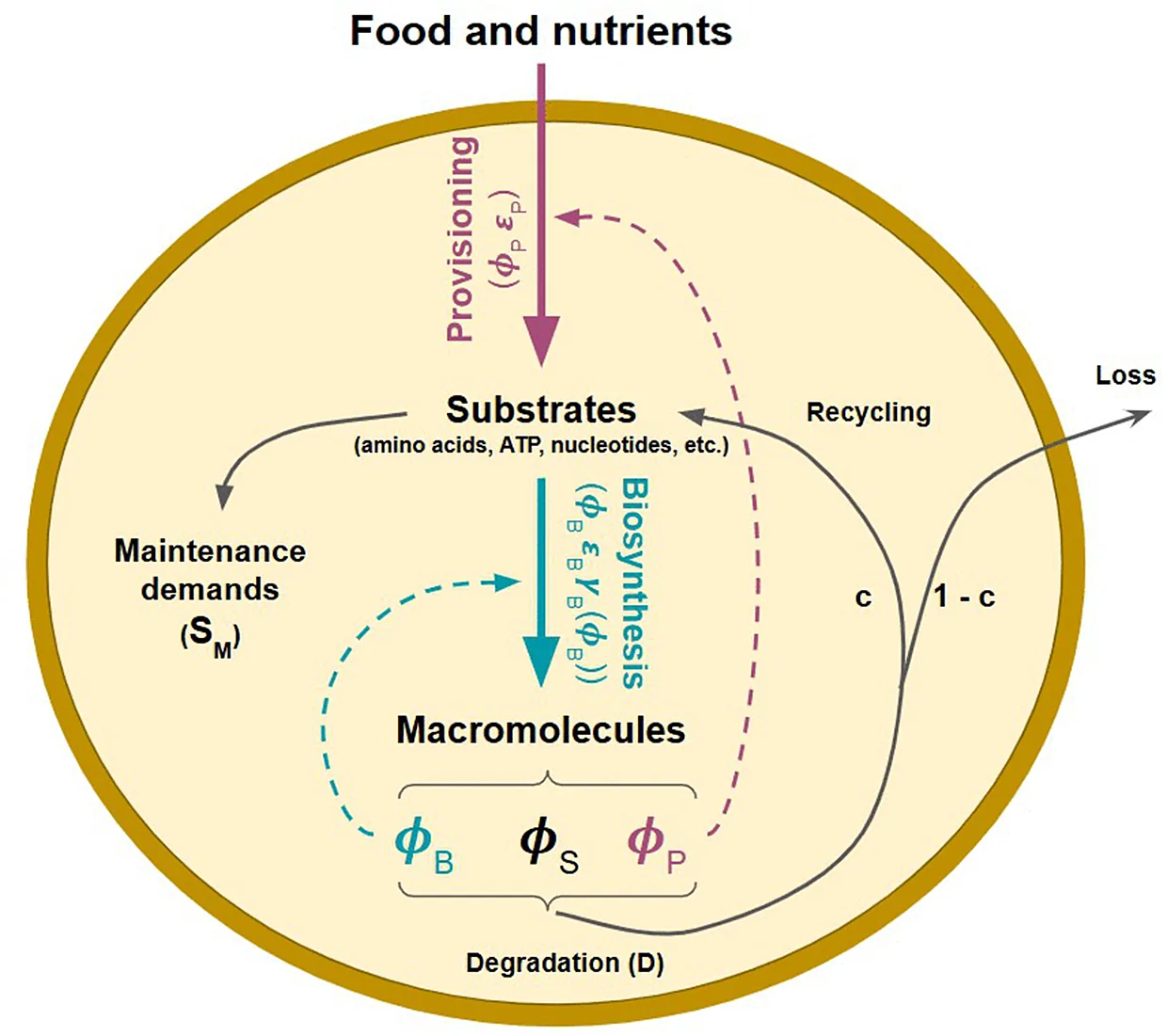
Cell diagram. We divide the macromolecules into three fractions: The provisioning fraction ($\phi_{P}$), the biosynthesis fraction ($\phi_{B}$), and the structural fraction ($\phi_{S}$). Specifically, the provisioning fraction ($\phi_{P}$) converts food and nutrients into substrates (i.e., energy stores like ATP, amino acids, nucleotides, monosaccharides, etc.) and consists of enzymes that break down food, produce various substrates, and the uptake pumps and channels. Substrates are then used for both maintenance tasks ($S_{M}$) and macromolecule production by the biosynthesis fraction ($\phi_{B}$), which consists of ribosomes, DNA and RNA polymerases, membrane and cell wall synthesis enzymes, and other macromolecules involved in polymerization. In addition, macromolecules tightly correlated in abundance with ribosomes or other biosynthesis enzymes, e.g., tRNAs and mRNAs, are part of this fraction [30,34]. The structural fraction ($\phi_{S}$) involves all the other macromolecules not directly involved in facilitating provisioning or biosynthesis, including DNA, membrane lipids, cell wall, storage macromolecules, half-made macromolecules, and proteins that help with structure, degradation, and maintenance. Degradation (*D*) breaks down macromolecules and partially recycles constituent substrates. Solid lines represent biomass flux, and dashed lines represent the enzymatic facilitation of biomass flux. The mass-standardized flux (flux in a cell divided by the mass of cell macromolecules) of different pathways is written inside parentheses.

**Fig 3:**
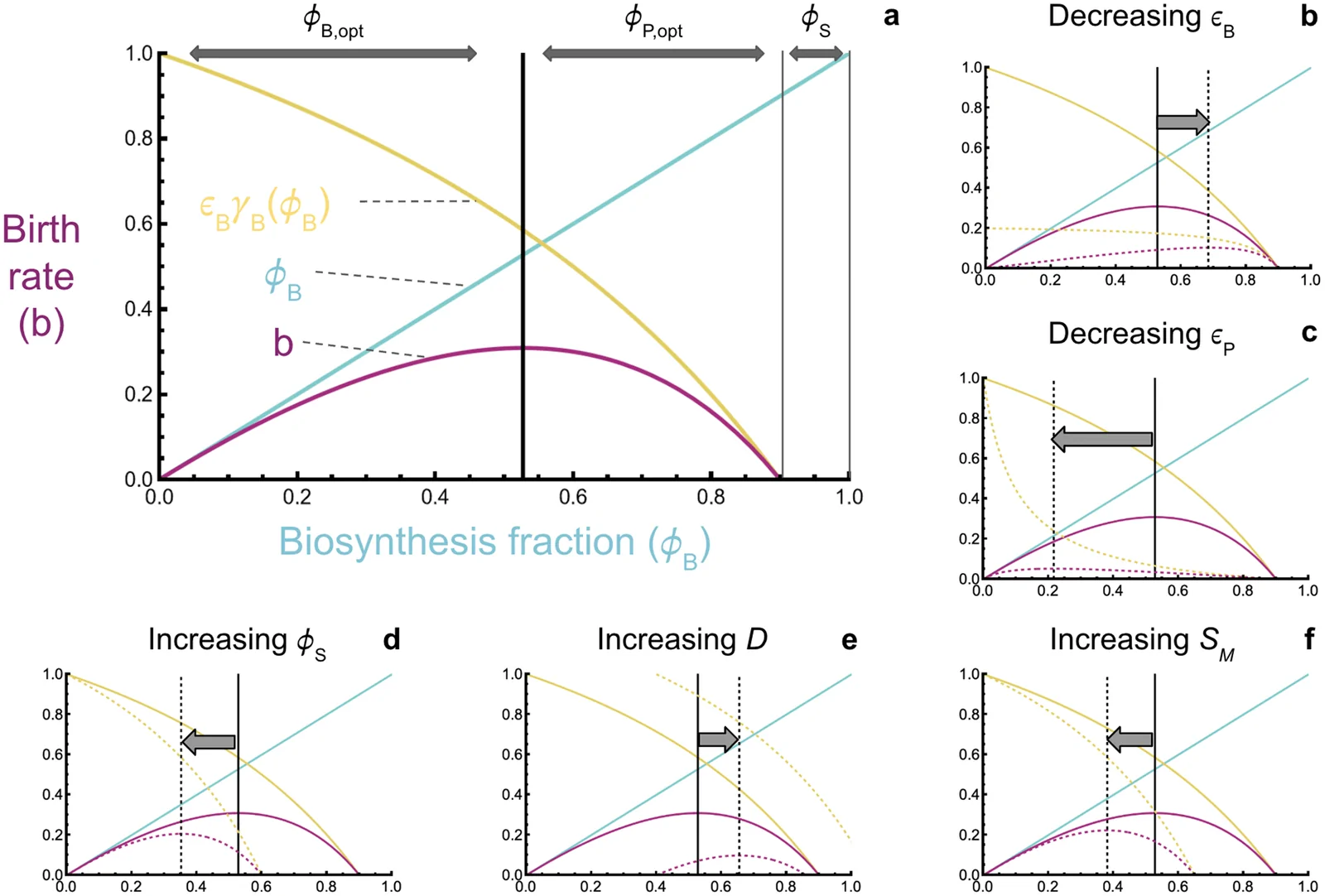
The trade-off between size and speed of the biosynthesis fraction. (**a**) As modeled in equation (1), the birth rate (*b*, purple humped curve) is the product of the biosynthesis fraction ($\phi_{B}$ along the x-axis, blue line) and the speed of biosynthetic machinery ($\epsilon_{B}\;\gamma_{B}(\phi_{B})$, yellow declining curve) minus the degradation rate. When the provisioning fraction is maximal (biosynthesis fraction near zero), the speed of biosynthetic machinery is maximal ($\epsilon_{B}$), but decreases as macromolecules are shifted from provisioning ($\phi_{P}$) to biosynthesis ($\phi_{B}$). This shift leads to the birth rate rising, however, until an optimal allocation is reached (vertical black bars). In panels b-f, dashed lines show the trade-off after varying one of the parameters, while solid lines represent the baseline (shown in panel **a**). Decreasing the biosynthesis efficiency ($\epsilon_{B}$ in panel **b**) increases the optimal biosynthesis fraction, however, decreasing the provisioning efficiency ($\epsilon_{P}$ in panel **c**) has the opposite effect. Increasing the structural fraction ($\phi_{S}$ in panel **d**) decreases the provisioning and biosynthesis fractions. Increasing the degradation rate (*D* in panel **e**) increases the optimal biosynthesis fraction (given that recycling is efficient), however, increasing maintenance demands ($S_{M}$ in panel **f**) decreases the optimal biosynthesis fraction. The following parameter values were used to obtain the solid lines: $\epsilon_{B}=1$, $\epsilon_{P}=2$, $\phi_{S}=0.1$, *D* = 0, $S_{M}=0$, and *c* = 0.8. Dashed lines in panels b-f differ in the following parameter values: panel b: $\epsilon_{B}=0.2$; panel c: $\epsilon_{P}=0.1$; panel d: $\phi_{S}=0.4$; panel e: *D* = 0.4; panel f: $S_{M}=0.5$. The code underlying this figure can be found at https://doi.org/10.5281/zenodo.22756291.

To proceed further, we must specify the nature of the trade-off, $\gamma_{B}(\phi_{B})$. In S1 Appendix, we add substrate dynamics to the model, which allows us to explicitly model the trade-off between generating substrates via provisioning or using them via biosynthesis. In the main text, however, we approximate the trade-off function, which allows us to derive interpretable analytical expressions for the optimal allocation. Specifically, we assume that the biosynthesis saturation level $\gamma_{B}(\phi_{B})$ rises linearly with the substrate supply rate ($M_{Total}(\phi_{P}\;\epsilon_{P}-S_{M}+c\;D)$) when the supply rate is small compared to the maximum rate of macromolecule production ($M_{Total}(\phi_{B}\;\epsilon_{B}-D)$), but approaches an asymptote of one as substrate supply rate increases:


$$\begin{array}{c} \gamma_{B}(\phi_{B})=\frac{1}{1+\frac{\phi_{B}\;\epsilon_{B}-D}{\phi_{P}\;\epsilon_{P}-S_{M}+c\;D}} \end{array}$$ (2)

(notice that $M_{Total}$ cancels out). When substrates are supplied into the substrate pool at a rate much faster than the maximum rate of macromolecule production ($\phi_{P}\;\epsilon_{P}-S_{M}+c\;D>>\phi_{B}\;\epsilon_{B}-D$), the ratio of substrates to macromolecules in the cell is high, and the biosynthetic enzymes become saturated ($\gamma_{B}(\phi_{B})=1$). On the other hand, when provisioning is limiting and the supply of substrates (including energy) is exhausted ($\phi_{P}\;\epsilon_{P}-S_{M}+c\;D<<\phi_{B}\;\epsilon_{B}-D$), the saturation level of biosynthetic enzymes approaches zero, and biosynthesis halts. Equation (2) is one of the main differences between our model and Scott and colleagues (2010), which assumes the saturation level of ribosomes is constant.

Under some parameter combinations, equation (2) can result in an unrealistically high rate of substrate production by the provisioning fraction, relative to the rate of substrate use by the biosynthesis fraction, which leads to a high concentration of substrates in the cytoplasm. This is, however, an artifact of the approximation used in equation (2). Explicitly tracking substrate concentrations leads to more realistic substrate concentrations (S1 Appendix). Nevertheless, the results obtained are qualitatively similar, except where noted.

By construction, this model has greatly simplified the features of a cell (Fig 2) in order to model growth rate under different conditions. Nevertheless, we highlight several limitations of this simplified model that could profitably be relaxed in future work. First, we hold the structural fraction constant, which may not be accurate, e.g., if altering the allocation between provisioning and biosynthesis alters cell size or shape in a manner that affects the surface area to volume ratio [35–37]. Second, we treat the stoichiometric composition of the provisioning, biosynthesis, and structural fractions as constant and similar, whereas their stoichiometric composition may vary in a manner that affects the optimal allocation. For example, if an element that is disproportionately found in the biosynthesis fraction, such as phosphorous, is scarce, the cell benefits less from allocating macromolecules to the biosynthesis fraction because the scarcity of that element ultimately limits the return on this investment [38] (discussed in greater detail in S1 Appendix). Finally, we assume that the cells are exponentially growing under constant environmental conditions. As environmental fluctuations can affect the optimal allocation [39–41], our results should be treated with caution in changing environments.

## Results

### Optimal allocation

Shifting the allocation of macromolecules between provisioning ($\phi_{P}$) and biosynthesis ($\phi_{B}$), while holding the structural fraction constant ($\phi_{P}+\phi_{B}=1-\phi_{S}$), cell birth rate is maximized at a single optimal allocation (Fig 3a), with selection favoring genetic or plastic changes that bring the cell closer to the optimum. To determine the optimal allocation, we find the biosynthesis fraction ($\phi_{B}$) where the derivative of the birth rate equation (1) with respect to $\phi_{B}$ equals 0. The optimal biosynthesis fraction thus equals:


$$\begin{array}{c} \phi_{B,opt}=\frac{\alpha -D}{\epsilon_{P}(1-\frac{\epsilon_{B}}{\epsilon_{P}})}\left(1-\sqrt{\frac{\frac{\epsilon_{B}}{\epsilon_{P}}\;\alpha -D}{\alpha -D}}\right) \end{array}$$ (3)

where $\alpha =\epsilon_{P}(1-\phi_{S})-S_{M}+c\;D$ is the maximum substrate supply rate (when the provisioning fraction is maximal), which must be positive if the birth rate is positive. To better understand this result, let us first focus on when degradation and maintenance demands are absent ($D=S_{M}=0$). Under this condition, the optimal biosynthesis fraction simplifies to $\left(1-\phi_{S})/(1+\sqrt{\frac{\epsilon_{B}}{\epsilon_{P}}}\right)$, and the optimal provisioning fraction (which equals $1-\phi_{S}-\phi_{B,opt}$) simplifies to $\sqrt{\frac{\epsilon_{B}}{\epsilon_{P}}}(1-\phi_{S})/(1+\sqrt{\frac{\epsilon_{B}}{\epsilon_{P}}})$. The ratio of the provisioning fraction over the biosynthesis fraction is thus inversely related to the ratio of provisioning over biosynthesis efficiency and equals:


$$\begin{array}{c} \frac{\phi_{P,opt}}{\phi_{B,opt}}=\sqrt{\frac{\epsilon_{B}}{\epsilon_{P}}} \end{array}$$ (4)

This shows that if, for example, the biosynthesis fraction is two times more efficient than the provisioning fraction ($\frac{\epsilon_{B}}{\epsilon_{P}}=2$), the provisioning fraction will be larger than the biosynthesis fraction but not large enough to completely compensate for the lower efficiency ($\phi_{P,opt}/\phi_{B,opt}=\sqrt{2}$), therefore the less efficient pathway remains more limiting. This highlights that optimal allocation is determined by a balance between the production of more efficient macromolecules and reducing the degree of limitation imposed by the less efficient pathway on macromolecule production.

When the degradation rate is low, the optimal allocation can be approximated by equations (5) and (6) to leading order in *D* (using first-order Taylor series expansion), which we use to guide our understanding of the impacts of maintenance demands ($S_{M}$), degradation (*D*), and recycling efficiency (*c*) (see Fig 3e and 3f).


$$\begin{array}{c} \phi_{B,opt}=\frac{(1-\phi_{S})+\frac{c\;D-S_{M}}{\epsilon_{P}}+\frac{\sqrt{\frac{\epsilon_{P}}{\epsilon_{B}}}-1}{2\;\epsilon_{P}}D}{1+\sqrt{\frac{\epsilon_{B}}{\epsilon_{P}}}} \end{array}$$ (5)

$$\begin{array}{c} \phi_{P,opt}=\frac{\sqrt{\frac{\epsilon_{B}}{\epsilon_{P}}}(1-\phi_{S})-\frac{c\;D-S_{M}}{\epsilon_{P}}-\frac{\sqrt{\frac{\epsilon_{P}}{\epsilon_{B}}}-1}{2\;\epsilon_{P}}D}{1+\sqrt{\frac{\epsilon_{B}}{\epsilon_{P}}}} \end{array}$$ (6)

The optimal provisioning fraction increases with increasing maintenance demands ($S_{M}$) or decreasing recycling (*c D*) to compensate for the lower substrate supply rate (Fig 3e and 3f). Notice that the larger the provisioning efficiency, the smaller the effect of recycling and maintenance demands on the optimal provisioning and biosynthesis fractions (see $\frac{c\;D-S_{M}}{\epsilon_{P}}$ in the numerator of equations (5) and (6)). This is because when provisioning machinery is fast, small changes in the provisioning fraction can compensate for the effect of recycling and maintenance demands on substrate supply.

Degradation affects the optimal allocation even when there is no recycling (see $\left(\sqrt{\frac{\epsilon_{P}}{\epsilon_{B}}}-1)D/(2\;\epsilon_{P}\right)$ in the numerator of equations (5) and (6)). This effect depends on $\sqrt{\frac{\epsilon_{B}}{\epsilon_{P}}}$, which is proportional to the steady-state substrate concentration when degradation rate is low (equation S5 in S1 Appendix). Increasing the degradation rate increases the optimal biosynthesis fraction when biosynthesis is more limiting ($\sqrt{\frac{\epsilon_{P}}{\epsilon_{B}}}>1$) and has the opposite effect when provisioning is more limiting ($\sqrt{\frac{\epsilon_{P}}{\epsilon_{B}}}<1$).

The saturation level of biosynthesis under optimal allocation can be calculated by plugging in the optimal biosynthesis fraction (equation (3)) into equation (2):


$$\begin{array}{c} \gamma_{B}(\phi_{B,opt})=\frac{1}{\left(1-\frac{\epsilon_{B}}{\epsilon_{P}}\right)}(1-\sqrt{\frac{\frac{\epsilon_{B}}{\epsilon_{P}}\;\alpha -D}{\alpha -D}}) \end{array}$$ (7)

Under optimal allocation and in the absence of degradation (*D* = 0), biosynthesis saturation level simplifies to $1/(1+\sqrt{\frac{\epsilon_{B}}{\epsilon_{P}}})$, which is maximal ($\gamma_{B}(\phi_{B,opt})=1$) when biosynthesis is limiting ($\epsilon_{P}>>\epsilon_{B}$), and approaches zero when provisioning is limiting ($\epsilon_{B}>>\epsilon_{P}$).

To determine the effect of degradation on the biosynthesis saturation level, we investigate its derivative with respect to *D* which equals $\frac{d\;\gamma_{B}(\phi_{B,opt})}{d\;D}=(\epsilon_{P}(1-\phi_{S})-S_{M})/(2(\alpha -D{)}^{2}\sqrt{\left(\frac{\epsilon_{B}}{\epsilon_{P}}\;\alpha -D)/(\alpha -D\right)})$. The denominator of this fraction must be positive, and the numerator must also be positive if the birth rate is positive (otherwise substrate loss is larger than the maximum substrate provisioning); therefore, degradation always increases biosynthesis saturation level. This is because the degradation rate reduces the reproduction rate and, consequently, the substrate supply to new cells, which in turn increases the concentration of substrates in the cytoplasm and the biosynthesis saturation level. The effect of maintenance demands on biosynthesis saturation level depends on the degradation rate ($\frac{d\;\gamma_{B}(\phi_{B,opt})}{d\;S_{M}}=D/(2(\alpha -D{)}^{2}\sqrt{\left(\frac{\epsilon_{B}}{\epsilon_{P}}\;\alpha -D)/(\alpha -D\right)})$). Interestingly, in the absence of degradation, maintenance demands do not affect the biosynthesis saturation level. This is because the effect of maintenance demands on the biosynthesis and provisioning fractions compensates for its impact on substrate supply.

To calculate the birth rate under optimal allocation ($b_{opt}$), we plug in $\phi_{B,opt}$ (equation (3)) and $\gamma_{B}(\phi_{B,opt})$ (equation (7)) into equation (1):


$$\begin{array}{c} b_{opt}=\frac{-D\left((\frac{\epsilon_{B}}{\epsilon_{P}}{)}^{2}+1\right)+\frac{\epsilon_{B}}{\epsilon_{P}}\left(\alpha (1+\frac{\epsilon_{B}}{\epsilon_{P}})-2\sqrt{\left(D-\alpha )(D-\alpha \frac{\epsilon_{B}}{\epsilon_{P}}\right)}\right)}{(\frac{\epsilon_{B}}{\epsilon_{P}}-1{)}^{2}} \end{array}$$ (8)

To facilitate interpretation, this expression can be approximated using a first-order Taylor series expansion with respect to the degradation rate, assuming the degradation rate is low:


$$\begin{array}{c} b_{opt}=R(1-\phi_{S})-S_{M}A-D\left(1-A(c+\sqrt{\frac{\epsilon_{P}}{\epsilon_{B}}})\right) \end{array}$$ (9)

where *R* (the return on investment into growth) equals $\epsilon_{B}/(1+\sqrt{\frac{\epsilon_{B}}{\epsilon_{P}}}{)}^{2}$, and *A* (measuring the extent to which provisioning is limiting) equals $\frac{\epsilon_{B}}{\epsilon_{P}}/(1+\sqrt{\frac{\epsilon_{B}}{\epsilon_{P}}}{)}^{2}$. Equation (9) indicates that the birth rate equals the fraction of macromolecules allocated to provisioning and biosynthesis ($1-\phi_{S}=\phi_{B}+\phi_{P}$) times the return on investment (*R*) minus the loss due to maintenance demands ($S_{M}\;A$) and degradation ($D(1-A(c+\sqrt{\frac{\epsilon_{P}}{\epsilon_{B}}}))$). The return on investment (*R*) is related to the harmonic mean of the provisioning and biosynthesis efficiencies ($\frac{1}{\sqrt{R}}=\frac{1}{\sqrt{\epsilon_{B}}}+\frac{1}{\sqrt{\epsilon_{P}}}$) and is closer to the efficiency of the less efficient process.

The effect of degradation and maintenance demands on the birth rate depends on which pathway is limiting. When biosynthesis is limiting ($\epsilon_{P}>>\epsilon_{B}$, A≈0), the reduction in the birth rate due to degradation is maximal and maintenance demands have almost no effect on the birth rate. On the other hand, when provisioning is limiting growth ($\epsilon_{P}<<\epsilon_{B},A\approx 1$), the effect of maintenance demands on the birth rate is maximal, while degradation’s effect depends on recycling efficiency. When the substrates are fully recycled after degradation (c≈1), the cost of degradation is minimal, however, when the substrates are not recycled (c≈0), the cost of degradation is high (Fig C in S1 Appendix).

### Cost of traits

For a trait conferring a given benefit (e.g., a given increase in survival), we provide a framework to investigate the cost of the trait. We also explore how this cost will be affected by provisioning and biosynthesis efficiency, which are determined by environmental quality and genetic background (Fig 4).

**Fig 4:**
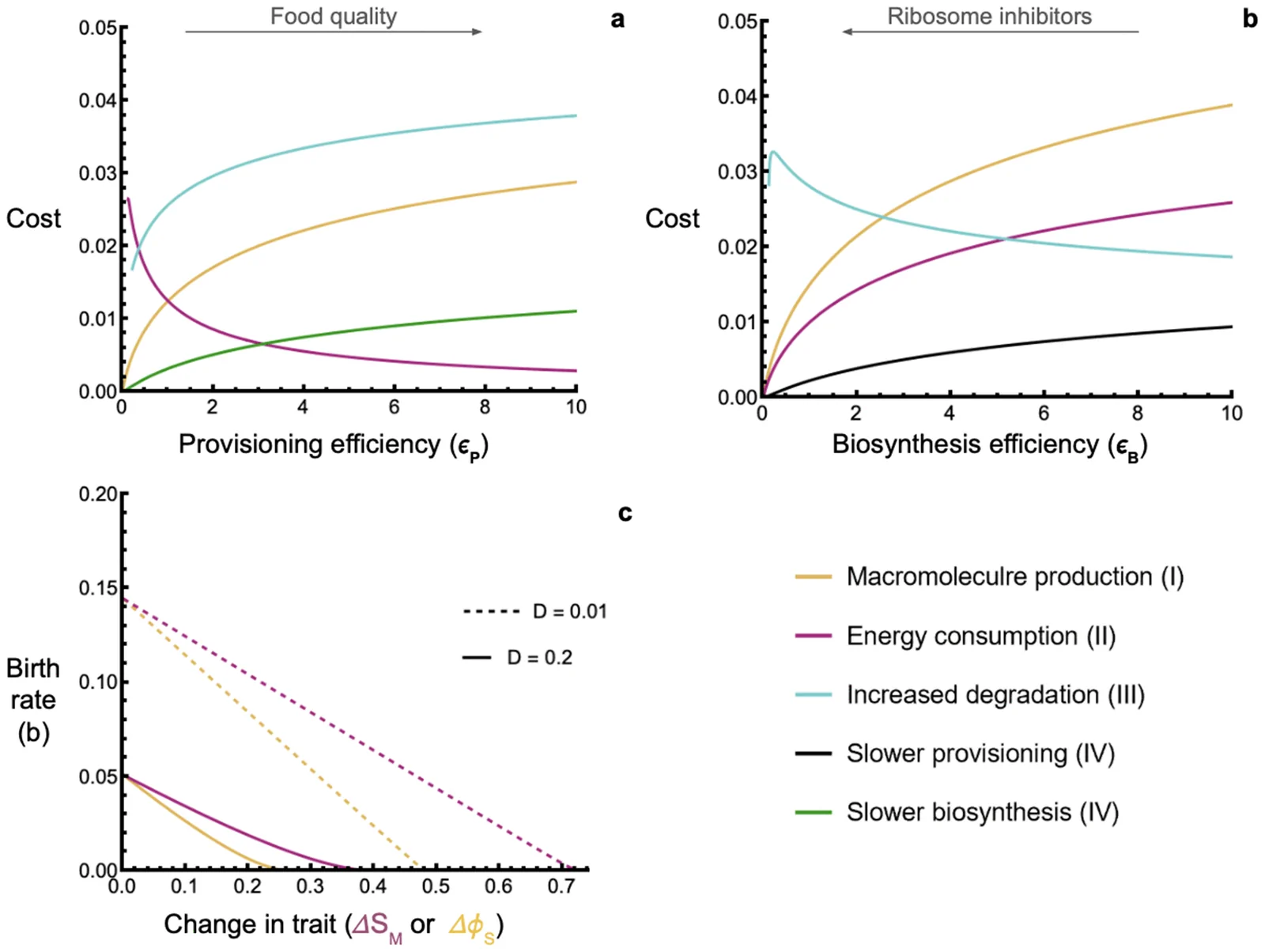
The effect of environmental quality on the cost of traits. The effect of altering provisioning efficiency (e.g., by food quality, panel **a**) and biosynthesis efficiency (e.g., by antibiotics that inhibit ribosomes, panel **b**) on the cost of different categories of traits (points I–IV). The cost is calculated as the absolute difference between the birth rate of a reference cell and a cell possessing the trait. The possession of the trait alters one of the parameter values: macromolecule production increases $\phi_{S}$, energy consumption increases $S_{M}$, increased degradation increases *D*, slower provisioning decreases $\epsilon_{P}$, and slower biosynthesis decreases $\epsilon_{B}$, all by 0.05. Panel c shows the relationship between the birth rate and the increase in allocation of macromolecules or energy to a trait. The parameter values for the reference cell are $\phi_{S}=0.5$, *c* = 0.8, *D* = 0.01 (panels a and **b)**, $S_{M}=0.005$, $\epsilon_{B}=1$ (panels a and **c)**, and $\epsilon_{P}=1.5$ (panels b and **c)**. Equation (8) was used to calculate the birth rate (not the Taylor series approximation). The code underlying this figure can be found at https://doi.org/10.5281/zenodo.22756291.

We consider the absolute reduction in birth rate as the evolutionarily relevant measure of cost (using equation (9)). For exponentially growing populations, the difference in birth rates ($b_{A}-b_{a}$) corresponds to the relative reduction in fitness within a single generation (*t* = 1), defining the relative fitness of cell *t*ype *A* to *a* as $e^{b_{A}t}/e^{b_{a}t}=e^{(b_{A}-b_{a})t}$, measured on a log scale. Below, we investigate the cost of four different examples of traits conferring stress-tolerance: (I) producing a macromolecule that mitigates stress (e.g., an enzyme degrading a toxin or a chelator neutralizing it). (II) Increasing energy consumption for maintenance (e.g., energy allocation for higher activity of preexisting pumps). (III) Increasing macromolecule degradation (e.g., to reduce the accumulation of damage in the cell). (IV) Using a slower metabolic pathway that reduces the cell’s vulnerability to stress.

- *I. Structural macromolecule allocation*: Allocating macromolecules for stress tolerance increases the structural fraction, therefore the change in birth rate equals $-\Delta \phi_{S}\;R$, where delta (Δ) symbolizes the change in a parameter. The lower the return on investment, the less costly the trait. In other words, the cost of allocating macromolecules to a trait depends on the growth that could have been achieved if those macromolecules were allocated to provisioning and biosynthesis. When the return on investment is higher, there is more to lose.
- *II. Higher energy allocation to maintenance*: Increasing the activity of preexisting pumps requires energy allocation and increases the maintenance demands, thus the change in birth rate equals $-\Delta S_{M}\;A$. The increase in maintenance demand is more costly when provisioning is limiting (A≈1). Any cost involving the allocation of energy or substrates (e.g., activating efflux pumps, feeding symbionts, and secreting metabolites) follows the same pattern.
- *III. Increased degradation rate*: The change in the birth rate due to an increase in the degradation rate depends on the recycling efficiency and the extent to which provisioning is limiting and equals $-\Delta D(1-A(c+\sqrt{\frac{\epsilon_{P}}{\epsilon_{B}}}))$. If recycling is efficient (c≈1), the cost of degradation is maximal when biosynthesis is limiting (A≈0). However, if recycling is inefficient (c≈0), the cost of degradation is overall high, and is relatively independent of provisioning and biosynthesis efficiencies (Fig C in S1 Appendix).
- *IV. Switching to slower pathways*: Switching to a slower metabolic pathway reduces the efficiency of provisioning, biosynthesis, or both, thereby lowering the return on investment (*R*) and altering the extent to which provisioning is limiting (*A*). The change in birth rate will thus be $\Delta R*(1-\phi_{S})-\Delta A\;(S_{M}-c\;D)+\Delta \left(A\sqrt{\frac{\epsilon_{P}}{\epsilon_{B}}}\right)\;D$. The more limiting a fraction is, the more detrimental reducing its efficiency would be on *R*. Therefore, as biosynthesis efficiency increases, the cost of slowing provisioning increases, and vice versa (Fig 4). Increasing the structural fraction reduces the provisioning and biosynthesis fractions, thus decreasing the cost of slowing down provisioning or biosynthesis. The cost of slowing down provisioning (ΔA>0) increases with maintenance demands and decreases with recycling. The opposite is true for the cost of slowing down biosynthesis (ΔA<0). Degradation has an additional effect on the cost of slowing down provisioning or biosynthesis, which depends on which pathway is more limiting ($\Delta \left(A\sqrt{\frac{\epsilon_{P}}{\epsilon_{B}}}\right)\;D$).

Another prediction of the energy allocation models is that the cost of a trait has a linear relationship with its energy requirement [2,6,7]. In Fig 4c, we show that as the allocation of energy or macromolecules to a trait increases, birth rate decreases linearly if the degradation rate is low (dashed curves), in line with this prediction (and equation (9)). However, when the degradation rate is relatively large (solid curves), the relationship becomes non-linear, particularly when growth rates are slow.

To summarize, this framework helps predict the relative costs of different types of traits, in addition to how these costs depend on environmental conditions and genetic background, a point to which we return in the Discussion.

### Bacterial growth laws

We next examine the correspondence between this model and the bacterial growth laws (Fig 1a). We assume cells have reached optimal allocation and focus on the relationship between birth rate ($b_{opt}$), biosynthesis fraction ($\phi_{B,opt}$), and biosynthesis saturation ($\gamma_{B}(\phi_{B,opt})$) (Fig 5). As the death rate is small during exponential growth (0.001–0.05 *h*^–1^ depending on the age of the cells [25]), the birth rate can be used as a proxy for the growth rate under the laboratory conditions used to study growth laws. Moreover, biosynthesis fraction and biosynthesis saturation level are correlated with the ribosome fraction in the proteome and the average protein production rate of ribosomes (which equals elongation rate times the fraction of active ribosomes), respectively, which we use as proxies. We consider manipulating provisioning efficiency here (e.g., via foods with different quality) and consider manipulating biosynthesis efficiency in S1 Appendix (e.g., via ribosome inhibitors).

**Fig 5:**
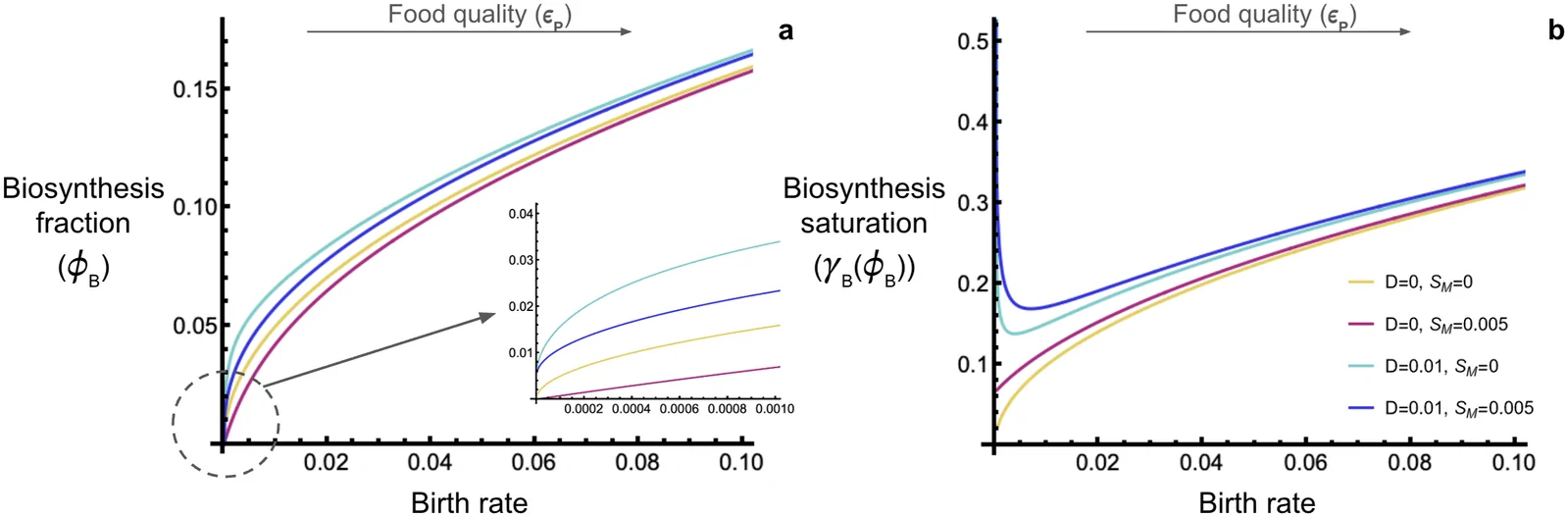
The effect of degradation, recycling, and maintenance demands on the shape of bacterial growth laws. Degradation, recycling, and maintenance demands affect the shape of bacterial growth laws, particularly during slow growth. To compare the model predictions with the growth laws, we used birth rate as a proxy for growth rate, the biosynthesis fraction (panel a) as a proxy for the ribosome fraction (Fig 1b), and the biosynthesis saturation level (panel b) as a proxy for the average protein production rate of ribosomes (which is correlated with elongation rate, Fig 1c). The exact equations (3) and (7) were used to calculate the birth rate, optimal biosynthesis fraction, and biosynthesis saturation (not the Taylor series approximation). Notice that during fast growth, the slope of all lines in panel a is similar and approximately equals $\frac{1}{2\;\epsilon_{B}}$. Other parameter values: $\phi_{S}=0.5$, $\epsilon_{B}=2$, and *c* = 0.8. The code underlying this figure can be found at https://doi.org/10.5281/zenodo.22756291.

In the absence of degradation and maintenance demands ($D=S_{M}=0$), as provisioning efficiency is increased, the birth rate, biosynthesis fraction, and the biosynthesis saturation level all increase (Fig 5a and 5b, yellow lines). Because the biosynthesis saturation level is variable, the relationship between birth rate and biosynthesis fraction is nonlinear, particularly during slow growth (Fig 5a, yellow line, whose slope is given by $\frac{d\;\phi_{B,opt}}{d\;b_{opt}}=\frac{\partial \phi_{B,opt}}{\partial \epsilon_{P}}/\frac{\partial b_{opt}}{\partial \epsilon_{P}}=(1+\sqrt{\frac{\epsilon_{B}}{\epsilon_{P}}})/(2\;\epsilon_{B})$). During fast growth, the biosynthesis fraction is saturated (because $\epsilon_{P}>>\epsilon_{B}$), therefore the slope approaches a constant value of $\frac{1}{2\epsilon_{B}}$. During slow growth, however, the biosynthesis fraction and saturation level approach zero, contradicting empirical observations (Fig 1a).

Allowing for degradation and maintenance demands leads to a broad range of predictions as growth slows. With only maintenance demands ($S_{M}>0$, *D* = 0), as the birth rate approaches zero, the biosynthesis saturation level approaches a non-zero value ($1/(1+\sqrt{\frac{\epsilon_{B}(1-\phi_{S})}{S_{M}}})$), in keeping with observed patterns (Fig 1c); the biosynthesis fraction continues to approach zero, however (Fig 5a and 5b, red lines).

In the presence of degradation (*D* > 0, regardless of $S_{M}$), the biosynthesis fraction and the biosynthesis saturation level approach non-zero values as growth rate slows to zero (with intercepts of $\frac{D}{\epsilon_{B}}$ and 1, respectively). The shape of these curves at low birth rates does not, however, match observations (compare Fig 5a and 5b, dark and light blue curves, to Fig 1a and 1c, respectively), with the predicted biosynthesis fraction being concave-down at low birth rates, and the predicted biosynthesis saturation level being concave-up. More empirical measurements at low growth rates would help determine whether these discrepancies are real, given that previous studies have not investigated very low growth rates (e.g., the smallest growth rate investigated by Dai and colleagues (2016) is 0.03 *h*^–1^ [19]). We return to the behavior of the growth laws at low growth in the Discussion. In the supplementary material, we motivate our choice of parameters (S1 Appendix), explore the parameter space more thoroughly (Figs E and F in S1 Appendix), and investigate an alternative model that explicitly tracks substrate concentration and yields qualitatively similar results (S1 Appendix).

## Discussion

The trade-off between allocation to biosynthesis and provisioning is a universal optimization problem all organisms face. By modeling this trade-off generally, the model we explore transitions smoothly between different factors limiting growth. This model can predict the fitness surface, i.e., the birth rate under different allocations (Fig 3), and the growth of an organism not at the optimum, as well as predicting optimal allocation patterns (Figs 4 and 5). Using this model, we predict the effect of environmental change and genetic background on the cost of various traits (Fig 4). Notably, we predict that the cost of traits that involve the production of a new macromolecule depends on the overall speed of provisioning and biosynthesis (point I). On the other hand, the cost of traits that require energy allocation (point II) or increased degradation (point III) depends on how limiting provisioning and biosynthesis are. Furthermore, we showed that the energetic demands of maintenance, degradation, and recycling affect the shape of bacterial growth laws, particularly during slow growth (Fig 5). Overall, we argue that combining provisioning and biosynthetic limitations provides a broader framework to think about the costs of traits than just focusing on energy or proteome allocation.

We expand proteome allocation models to incorporate all macromolecules rather than just proteins, and suggest that the relationship between the ribosome fraction and growth rate is a specific case of the more general relationship between the biosynthesis fraction and growth rate. This agrees with previous findings that other biosynthetic enzymes, such as RNA polymerases and cell envelope biosynthesis enzymes, increase as growth accelerates [17,21,22,34,36,42]). We thus demonstrate that the bacterial growth laws can be explained without assuming that protein production or any other specific process is the limiting step in growth, suggesting such patterns could be prevalent across a wide range of biological systems. Our work thus complements previous studies that investigated the limitations imposed by transcription, cell envelope synthesis, and other processes on growth [17,36,43], without making the assumption that protein production is the main limiting step in growth [30,10,13–15,30]. Overall, by incorporating all macromolecules into the proteome allocation theory, our work facilitates further synthesis between this theory and other theories of growth that are not limited to proteins, such as the metabolic theory of ecology (which is related to the energy allocation framework) [7,44–47]. Moreover, incorporating explicitly how the optimal allocation depends on the availability of particular nutrients (e.g., nitrogen and phosphorous) might allow further synthesis with ecological stoichiometry theory, which is a promising direction for future studies [48–50].

Our model shows that a framework focusing only on energy allocation will overestimate the cost of traits unless growth is limited primarily by resource provisioning (A≈1). Although some energy allocation models account for the opportunity cost of metabolites used for biomass production, they assume the total energy (or carbon) budget is limited [5], which is equivalent to assuming provisioning is limiting. If, instead, the rate of biosynthesis limits cell reproduction (A≈0), then the energetic cost of a trait does not proportionately affect the rate of reproduction. For instance, in *E.coli* and yeast, ribosome-associated proteins comprise between 10% (slow growth) to 40% (fast growth) of the proteome, while the allocation to glycolysis and energy production varies between 10% (fast growth) to 30% (slow growth) [22,23]. Notable allocation to ribosomal proteins suggests a high level of biosynthesis limitation, particularly during fast growth (or biosynthesis inhibition). To clarify this point, consider a biosynthesis-limited cell (A≈0) that allocates half of its substrates (or energy) to a trait. According to the energy allocation framework, this trait should halve the cell growth rate [2,5,6], however, our model predicts a smaller cost. This is because the cell can increase its smaller provisioning fraction to supply the trait, with minimal impact on the biosynthesis fraction, which limits growth rate. Therefore, it is necessary to determine to what extent provisioning limits growth (e.g., using the RNA-to-Protein ratio or the fraction of ribosomes) when using the energy-allocation framework to determine the cost of traits. Nevertheless, our model predicts that when the degradation rate is low, the cost of traits scales linearly with their energy or macromolecule requirements (i.e., doubling the energy requirement doubles the cost), in agreement with energy allocation models [2,6,7].

While our model provides a unified framework to understand the costs of different traits and patterns observed (the bacterial growth laws) when growing rapidly, our model does not fully reproduce the shape of growth laws during slow growth (compare curvatures of Figs 1a and 5a). This discrepancy suggests that some of our assumptions might be less accurate during slow growth (e.g., constant structural fraction and similar stoichiometric composition of different fractions) or that additional processes are playing an important role. For example, it has been suggested that the degradation rate increases during slow growth, which could explain the concave-up shape of the growth laws during slow growth [29](but a more recent study disagrees [51]). Alternatively, limitations in cell surface area could play a role [52,53], or cells may regulate the ribosome fractions in a manner that is non-optimal [33]. It has also been suggested that cells have evolved to grow in fluctuating environments and maintain more ribosomes than needed during starvation, which is beneficial upon increased food availability [19,23,39,43]. Our model supports the last explanation by showing that when food quality is very low, having a higher than optimal biosynthesis fraction results in a relatively small reduction in birth rate (i.e., the fitness surface is asymmetric; Fig 3c). Moreover, the regulation of gene expression is noisy in bacteria, and there is considerable heterogeneity in the allocation among individual cells [54]. As the fitness surface is assymetric (Fig 3c), selection acting on this heterogeneity over time or among cells may have resulted in a higher-than-optimal average biosynthesis fraction in the population [55,56]. Nevertheless, a recent study has argued that previous empirical studies might have overestimated the ribosome fraction during slow growth by not giving cells enough time to reach steady state before measurement [14], casting doubt on whether growth laws are concave-up during slow growth.

Our model can predict the effect of environmental quality on the type of traits more likely to evolve (points I–IV, Fig 4). All else being equal, a trait is more likely to evolve under conditions where it is less costly. Our model predicts that the cost of structural traits that require macromolecule allocation is the lowest in low-quality environments, where growth is already slow due to low food quality or slow biosynthesis (point I). On the other hand, the cost of energy allocation (which increases maintenance demands) is lowest when biosynthesis is limiting (due to high food quality or slow biosynthesis, point II). Alternatively, the cost of degradation is minimal when recycling is efficient and provisioning is limiting (due to low food quality or fast biosynthesis, point III). Lastly, the cost of slowing down provisioning (or biosynthesis) is the least when it is not limiting and the structural fraction is large (point IV). Furthermore, our model can make qualitative predictions about the likelihood of stress tolerance evolution in a specific environment based on the types of possible mutations. For example, if becoming resistant to a stressor is only possible by traits that require energy allocation, we predict resistance to evolve more readily in environments where food quality is high or biosynthesis is inhibited.

Our work highlights that to understand the effect of environmental quality on the cost of traits, multiple dimensions of environmental quality should be considered simultaneously. For example, although decreasing food quality (i.e., provisioning efficiency) increases the cost of energy-consuming traits, inhibiting biosynthesis (e.g., by antibiotics) has the opposite effect (point II). The same applies to the effect of genetic background on the cost of traits. A recent study did not find any meaningful relationship between the cost of traits and environmental quality, which may be because the environments differed in many factors with confounding effects on provisioning and biosynthesis efficiency [4]. We recommend that future studies determine to what degree provisioning and biosynthesis are limiting growth in different environments (using RNA-to-protein ratio as a potential proxy), and compare the cost of traits in similar environments that only differ in provisioning or biosynthesis efficiency. While these efficiencies are abstract quantities that cannot be directly measured, they can be inferred from the slope of the relationship between the biosynthesis fraction and the birth rate during fast growth (e.g., a slope of $\frac{1}{2\;\epsilon_{B}}$ in Fig 5a).

Biomass production is a fundamental process required for the growth and reproduction of all living organisms. Our model may thus shed light on the trade-off between allocation to provisioning, biosynthesis, and structure at other levels of organization. For example, the prevalence of structural adaptations in desert-living plants (e.g., thorns) might be partially explained by the lower cost of structural traits in low-quality environments (or slow life-histories) for growth (point I). Wherever biomass production is maximized, insights from this model might be applicable.

## Supporting information

S1 Appendix Supporting information containing assumptions and limitations, estimation of parameter values, growth laws when partially inhibiting biosynthesis, and a model with explicit substrate dynamics. (PDF)

S1 File Code for derivations and reproducing graphs (Mathematica notebook and PDF). (ZIP)

## References

[1] Dekel E, Alon U. Optimality and evolutionary tuning of the expression level of a protein. Nature. 2005;436(7050):588–92. doi: 10.1038/nature03842 16049495

[2] Schavemaker PE, Lynch M. Bioenergetics and the evolution of cellular traits. Annu Rev Biophys. 2025;54(1):81–99. doi: 10.1146/annurev-biophys-070524-090334 40327439 PMC12921477

[3] Melnyk AH, Wong A, Kassen R. The fitness costs of antibiotic resistance mutations. Evol Appl. 2015;8(3):273–83. doi: 10.1111/eva.12196 25861385 PMC4380921

[4] Hinz A, Amado A, Kassen R, Bank C, Wong A. Unpredictability of the fitness effects of antimicrobial resistance mutations across environments in *Escherichia coli*. Mol Biol Evol. 2024;41(5):msae086. doi: 10.1093/molbev/msae086 38709811 PMC11110942

[5] Lynch M, Trickovic B. A Theoretical framework for evolutionary cell biology. J Mol Biol. 2020;432(7):1861–79. doi: 10.1016/j.jmb.2020.02.006 32087200 PMC8025685

[6] Lynch M, Marinov GK. The bioenergetic costs of a gene. Proc Natl Acad Sci U S A. 2015;112(51):15690–5. doi: 10.1073/pnas.1514974112 26575626 PMC4697398

[7] Ilker E, Hinczewski M. Modeling the growth of organisms validates a general relation between metabolic costs and natural selection. Phys Rev Lett. 2019;122(23):238101. doi: 10.1103/PhysRevLett.122.238101 31298905

[8] Craig CL, Weber RS. Selection costs of amino acid substitutions in ColE1 and ColIa gene clusters harbored by *Escherichia coli*. Mol Biol Evol. 1998;15(6):774–6. doi: 10.1093/oxfordjournals.molbev.a025981 9615459

[9] Molenaar D, van Berlo R, de Ridder D, Teusink B. Shifts in growth strategies reflect tradeoffs in cellular economics. Mol Syst Biol. 2009;5:323. doi: 10.1038/msb.2009.82 19888218 PMC2795476

[10] Scott M, et al. Interdependence of cell growth and gene expression: origins and consequences. Science. 2010;330(6007):1099–102. 10.1126/science.1192588. 21097934

[11] Zaslaver A, Kaplan S, Bren A, Jinich A, Mayo A, Dekel E, et al. Invariant distribution of promoter activities in *Escherichia coli*. PLoS Comput Biol. 2009;5(10):e1000545. doi: 10.1371/journal.pcbi.1000545 19851443 PMC2758578

[12] Berkhout J, et al. How biochemical constraints of cellular growth shape evolutionary adaptations in metabolism. Genetics. 2013;194(2):505–12. doi: 10.1534/genetics.113.150631 PMC3664859 23535382

[13] Kafri M, Metzl-Raz E, Jonas F, Barkai N. Rethinking cell growth models. FEMS Yeast Res. 2016;16(7):fow081. doi: 10.1093/femsyr/fow081 27650704

[14] Chure G, Cremer J. An optimal regulation of fluxes dictates microbial growth in and out of steady state. eLife. 2023;12:e84878. doi: 10.7554/eLife.84878 PMC10110240 36896805

[15] Weiße AY, Oyarzún DA, Danos V, Swain PS. Mechanistic links between cellular trade-offs, gene expression, and growth. Proc Natl Acad Sci U S A. 2015;112(9):E1038-47. doi: 10.1073/pnas.1416533112 25695966 PMC4352769

[16] Basan M, Hui S, Okano H, Zhang Z, Shen Y, Williamson JR, et al. Overflow metabolism in *Escherichia coli* results from efficient proteome allocation. Nature. 2015;528(7580):99–104. doi: 10.1038/nature15765 26632588 PMC4843128

[17] Belliveau NM, Chure G, Hueschen CL, Garcia HG, Kondev J, Fisher DS, et al. Fundamental limits on the rate of bacterial growth and their influence on proteomic composition. Cell Syst. 2021;12(9):924-944.e2. doi: 10.1016/j.cels.2021.06.002 34214468 PMC8460600

[18] rchaechte R M, Maaloe O, Kjeldgaard N O. Dependency on medium and temperature of cell size and chemical composition during balanced grown of Salmonella typhimurium. J Gen Microbiol. 1958;19(3):592–606. doi: 10.1099/00221287-19-3-592 13611202

[19] Dai X, Zhu M, Warren M, Balakrishnan R, Patsalo V, Okano H, et al. Reduction of translating ribosomes enables Escherichia coli to maintain elongation rates during slow growth. Nat Microbiol. 2016;2:16231. doi: 10.1038/nmicrobiol.2016.231 27941827 PMC5346290

[20] Hui S, Silverman JM, Chen SS, Erickson DW, Basan M, Wang J, et al. Quantitative proteomic analysis reveals a simple strategy of global resource allocation in bacteria. Mol Syst Biol. 2015;11(1):784. doi: 10.15252/msb.20145697 25678603 PMC4358657

[21] Peebo K, Valgepea K, Maser A, Nahku R, Adamberg K, Vilu R. Proteome reallocation in *Escherichia coli* with increasing specific growth rate. Mol Biosyst. 2015;11(4):1184–93. doi: 10.1039/c4mb00721b 25712329

[22] Schmidt A, Kochanowski K, Vedelaar S, Ahrné E, Volkmer B, Callipo L, et al. The quantitative and condition-dependent *Escherichia coli* proteome. Nat Biotechnol. 2016;34(1):104–10. doi: 10.1038/nbt.3418 26641532 PMC4888949

[23] Metzl-Raz E, et al. Principles of cellular resource allocation revealed by condition-dependent proteome profiling. eLife. 2017;6:e28034. doi: 10.7554/eLife.28034 PMC5578734 28857745

[24] Mori M, Zhang Z, Banaei-Esfahani A, Lalanne J-B, Okano H, Collins BC, et al. From coarse to fine: the absolute *Escherichia coli* proteome under diverse growth conditions. Mol Syst Biol. 2021;17(5):e9536. doi: 10.15252/msb.20209536 34032011 PMC8144880

[25] Wang P, Robert L, Pelletier J, Dang WL, Taddei F, Wright A, et al. Robust growth of *Escherichia coli*. Curr Biol. 2010;20(12):1099–103. doi: 10.1016/j.cub.2010.04.045 20537537 PMC2902570

[26] Dalbow DG, Young R. Synthesis time of beta-galactosidase in *Escherichia coli* B/r as a function of growth rate. Biochem J. 1975;150(1):13–20. doi: 10.1042/bj1500013 1106403 PMC1165698

[27] Young R, Bremer H. Polypeptide-chain-elongation rate in *Escherichia coli* B/r as a function of growth rate. Biochem J. 1976;160(2):185–94. doi: 10.1042/bj1600185 795428 PMC1164221

[28] Pedersen S. Escherichia coli ribosomes translate in vivo with variable rate. EMBO J. 1984;3(12):2895–8. doi: 10.1002/j.1460-2075.1984.tb02227.x 6396082 PMC557784

[29] Calabrese L, Grilli J, Osella M, Kempes CP, Lagomarsino MC, Ciandrini L. Protein degradation sets the fraction of active ribosomes at vanishing growth. PLoS Comput Biol. 2022;18(5):e1010059. doi: 10.1371/journal.pcbi.1010059 35500024 PMC9098079

[30] Klumpp S, Scott M, Pedersen S, Hwa T. Molecular crowding limits translation and cell growth. Proc Natl Acad Sci U S A. 2013;110(42):16754–9. doi: 10.1073/pnas.1310377110 24082144 PMC3801028

[31] Pandey PP, Jain S. Analytic derivation of bacterial growth laws from a simple model of intracellular chemical dynamics. Theory Biosci. 2016;135(3):121–30. doi: 10.1007/s12064-016-0227-9 27167220 PMC5018264

[32] You C, Okano H, Hui S, Zhang Z, Kim M, Gunderson CW, et al. Coordination of bacterial proteome with metabolism by cyclic AMP signalling. Nature. 2013;500(7462):301–6. doi: 10.1038/nature12446 23925119 PMC4038431

[33] Wu C, Balakrishnan R, Braniff N, Mori M, Manzanarez G, Zhang Z, et al. Cellular perception of growth rate and the mechanistic origin of bacterial growth law. Proc Natl Acad Sci U S A. 2022;119(20):e2201585119. doi: 10.1073/pnas.2201585119 35544692 PMC9171811

[34] Balakrishnan R, Mori M, Segota I, Zhang Z, Aebersold R, Ludwig C, et al. Principles of gene regulation quantitatively connect DNA to RNA and proteins in bacteria. Science. 2022;378(6624):eabk2066. doi: 10.1126/science.abk2066 36480614 PMC9804519

[35] Bertaux F, von Kügelgen J, Marguerat S, Shahrezaei V. A bacterial size law revealed by a coarse-grained model of cell physiology. PLoS Comput Biol. 2020;16(9):e1008245. doi: 10.1371/journal.pcbi.1008245 32986690 PMC7553314

[36] Trickovic B, Lynch M. Resource allocation to cell envelopes and the scaling of bacterial growth rate. Phys Biol. 2025;22(4):10.1088/1478-3975/adea04. doi: 10.1088/1478-3975/adea04 40588006 PMC13014474

[37] Chure G, de Silva RT, Sharma R, Lanz MC, Cremer J. Maintenance of cytoplasmic and membrane densities shapes cellular geometry in *Escherichia coli*. Nat Commun. 2025;16(1):11557. doi: 10.1038/s41467-025-67553-3 41429793 PMC12749242

[38] Li SH-J, Li Z, Park JO, King CG, Rabinowitz JD, Wingreen NS, et al. Escherichia coli translation strategies differ across carbon, nitrogen and phosphorus limitation conditions. Nat Microbiol. 2018;3(8):939–47. doi: 10.1038/s41564-018-0199-2 30038306 PMC6278830

[39] Mori M, Schink S, Erickson DW, Gerland U, Hwa T. Quantifying the benefit of a proteome reserve in fluctuating environments. Nat Commun. 2017;8(1):1225. doi: 10.1038/s41467-017-01242-8 29089487 PMC5663898

[40] Cylke A, Serbanescu D, Banerjee S. Energy allocation theory for bacterial growth control in and out of steady state. Proc R Soc A. 2024;480(2300). doi: 10.1098/rspa.2024.0219

[41] Perez L, Cremer J. A mismatch between slow protein synthesis and fast environmental fluctuations determines tradeoffs in bacterial proteome allocation strategies. bioRxiv. 2025;:2025.07.22.666192. doi: 10.1101/2025.07.22.666192 42124636 PMC13160198

[42] Bremer H, Dennis PP. Modulation of Chemical Composition and Other Parameters of the Cell at Different Exponential Growth Rates. EcoSal Plus. 2008 Sep;3(1). 10.1128/ecosal.5.2.3. 26443740

[43] Kafri M, et al. The cost of protein production. Cell Reports. 2016 Jan;14(1):22–31. doi: 10.1016/j.celrep.2015.12.015 PMC4709330 26725116

[44] Kempes CP, Dutkiewicz S, Follows MJ. Growth, metabolic partitioning, and the size of microorganisms. Proc Natl Acad Sci U S A. 2012;109(2):495–500. doi: 10.1073/pnas.1115585109 22203990 PMC3258615

[45] Kempes CP, Wang L, Amend JP, Doyle J, Hoehler T. Evolutionary tradeoffs in cellular composition across diverse bacteria. ISME J. 2016;10(9):2145–57. doi: 10.1038/ismej.2016.21 27046336 PMC4989312

[46] West GB, Brown JH, Enquist BJ. A general model for the origin of allometric scaling laws in biology. Science. 1997;276(5309):122–6. doi: 10.1126/science.276.5309.122 9082983

[47] West GB, Brown JH, Enquist BJ. A general model for ontogenetic growth. Nature. 2001;413(6856):628–31. doi: 10.1038/35098076 11675785

[48] Allen AP, Gillooly JF. Towards an integration of ecological stoichiometry and the metabolic theory of ecology to better understand nutrient cycling. Ecol Lett. 2009;12(5):369–84. doi: 10.1111/j.1461-0248.2009.01302.x 19379132

[49] Klausmeier CA, Litchman E, Daufresne T, Levin SA. Optimal nitrogen-to-phosphorus stoichiometry of phytoplankton. Nature. 2004;429(6988):171–4. doi: 10.1038/nature02454 15141209

[50] Moreno AR, Hagstrom GI, Primeau FW, Levin SA, Martiny AC. Marine phytoplankton stoichiometry mediates nonlinear interactions between nutrient supply, temperature, and atmospheric CO 2. Biogeosciences. 2018;15(9):2761–79. doi: 10.5194/bg-15-2761-2018

[51] Gupta M, Johnson ANT, Cruz ER, Costa EJ, Guest RL, Li SH-J, et al. Global protein turnover quantification in Escherichia coli reveals cytoplasmic recycling under nitrogen limitation. Nat Commun. 2024;15(1):5890. doi: 10.1038/s41467-024-49920-8 39003262 PMC11246515

[52] Szenk M, Dill KA, De Graff AMR. Why do fast-growing bacteria enter overflow metabolism? Testing the membrane real estate hypothesis. Cell Systems. 2017;5(2):95–104. doi: 10.1016/j.cels.2017.06.005 28755958

[53] Schavemaker PE, Lynch M. Quantifying the evolutionary paths to endomembranes. Cell Rep. 2025;44(4):115533. doi: 10.1016/j.celrep.2025.115533 40198222 PMC12124869

[54] Ackermann M. A functional perspective on phenotypic heterogeneity in microorganisms. Nat Rev Microbiol. 2015;13(8):497–508. doi: 10.1038/nrmicro3491 26145732

[55] Choi HJ, Lo TW, Cutler KJ, Huang D, Will WR, Wiggins PA. Protein overabundance is driven by growth robustness. Sci Adv. 2026;12(12):eadz9623. doi: 10.1126/sciadv.adz9623 41861001 PMC13004021

[56] Lo TW, Choi HJ, Huang D, Wiggins PA. Noise robustness and metabolic load determine the principles of central dogma regulation. Sci Adv. 2024;10(34):eado3095. doi: 10.1126/sciadv.ado3095 39178264 PMC11343026

