# Supplementary material for: Bridging energy and proteome allocation models allows prediction of the resource cost of traits across environments
Source: PLoS Biol. 2026 Oct 13;24(10):e3003995. doi: 10.1371/journal.pbio.3003995 (PMC13660896; doi:10.1371/journal.pbio.3003995)
Supplement: Supplementary file 2 [file pbio.3003995.s003.pdf]

1 To the Editorial team

Dear Editors,

We appreciate all the efforts by you and reviewers to improve our work. Both reviewers provided insightful feedback, which helped us strengthen our work. Below, you can find the reviewer comments (in black), in addition to our response to the comments and a brief summary of changes we have made to address the issues or improve clarity (in red). Moreover, we have made some minor changes throughout the manuscript to improve its clarity and flow. Finally, we cited Schavemaker and Lynch (2025b)[44] and Szenk et al. (2017)[47] as suggested by the reviewers, and included the following citations to better connect our work to the existing and recent literature: Bertaux et al. (2020)[7], Choi et al. (2026)[10], Chure et al. (2025)[12], Cylke et al. (2025)[14], Lo et al. (2024)[29], Mori et al. (2017)[37], Perez and Cremer (2025) [41].

2 REVIEWS

2.1 Reviewer #1

To understand how specific cellular traits evolve and why they persist, it is important to account for both benefits and costs, to obtain a net benefit. The introduction of a new trait is almost always a drain on one or more cellular resources. This could be the cell energy budget or biosynthesis capacity, but also the cell volume, surface area, or the availability of specific limiting elements like N, P, or Fe. It has remained a puzzle how drains on a combination of these various resources collectively affect the growth rate (and/or survival) of a cell, and therefore it is unclear what cost is associated with the introduction of a new cellular trait.

In the present work, the authors tackle this important problem and present a theoretical framework that accounts for both the cell energy budget and the biosynthesis capacity. They build on the proteome allocation model approach that has been used in the past by various other authors. Such models typically subdivide the proteome in

a fraction that produces proteins, a fraction that produces substrates, and an "other" fraction that houses all other processes. The authors present a similar model but include (1) a drain on the substrate pool that represents maintenance costs, (2) a drain on the macromolecule pool that represents degradation (with an adjustable degree of recycling substrates), and (3) a degree of saturation of the biosynthesis machinery that depends (directly or indirectly) on the pool of substrates. Addition (1) and (2) introduce an important aspect of energy allocation models into the proteome allocation model.

The resulting model can be used to predict the optimal partitioning between the two nonstructural fractions (biosynthesis and provisioning) as well as the birthrate at this optimum. The model can also be used to examine and the consequences of various alterations to the parameters to this optimal partitioning and the birthrate. The structural fraction (ϕ_S), degradation rate (D), and maintenance demands (SM) are parameters of particular importance in analyzing the fitness consequences of adding a new cellular trait, and it is revealed in this study that these have rather different consequences. It is also revealed that the impact of various cellular alterations depends on the starting size of the fractions that are affected by these alterations, a finding that is intuitive after reading through the analysis presented in this work.

I recommend publishing this paper, with minor adjustments.

Issues that need to be addressed:

1) It is stated in line 92 that "we include all macromolecules in our model rather than just proteins". This is mentioned as one of the key differences between the present model and the previous proteome allocation models. However, it is not clear how the previous models specifically include proteins and how this model includes also other macromolecules. Is this related to the parameter values? Or, is there a structural difference in the model that accounts for this difference? Since this is one of the three key differences between this and previous work, this needs to be clarified.

We appreciated this suggestion and clarified how we defined macromolecules (and why), specifically on lines 106-108.

2) There appears to be a mismatch by the rate of substrates produced by the

provisioning fraction and the rate of substrates utilized by the biosynthesis fraction. I calculate the substrate production rate from the equation on line 118 and the biosynthesis rate from the equation on line 120, finding that substrate production is $\tilde{2}$.4-fold higher than its use in biosynthesis at the optimal biosynthesis fraction (using eq 1, parameters as in Fig 3a). This means that either 60% of all substrates that are produced are lost, or that these form a really large pool of substrates in the cell, or some combination of the two. Whether this is really a problem depends on what the physiological ranges of the other parameters are, as the quantity of the excess substrate production is affected by the parameter choices. Perhaps the authors can explain this discrepancy and offer a reason for why this is not a problem. If no such reason can be given there should perhaps be an additional constraint in the model that includes the size of the substrate pool, for example by making substrates a separate biomass fraction.

We appreciate the point that the reviewer has raised. It is true that the model in the main text can imply very high concentration of substrates relative to macromolecules in cytoplasm under some parameter combinations, but this is an artifact of the approximation used in the main text ($K_B/\rho = 1$). The model explored in S4 text, however, accounts for substrate dynamics more realistically and is more robust to this issue. The results are largely the same, as discussed in S4 Text. We now explicitly point out that one of the issues of the simplified model in the text is that it can lead to unreasonably high substrate concentrations and point the reader to the sup mat in these cases on lines 169-175.

3) The description of the energy allocation model is incomplete as it contains only the maintenance aspect. It is stated that "Two different frameworks have been proposed to quantify the resource costs of traits, focusing on either energy allocation or proteome allocation. These frameworks implicitly assume energy provisioning or protein production limits growth, respectively". The energy allocation framework used by Lynch and Marinov (2015) contains both a maintenance cost component and a growth (or construction) cost component. The maintenance cost component functions as the authors present. However, the growth cost component probably functions more like a proteome

allocation model. This is because the growth costs are mostly opportunity costs, i.e. food molecules diverted to making carbon backbones for amino acids in proteins, etc. The difference between the proteome allocation model and the energy allocation model being that in proteome allocation models, biosynthesis is modeled explicitly whereas in the energy allocation models biosynthesis is implicit. Despite this quibble, I think the authors made a novel and important contribution in uniting the two perspectives.

The reviewer is right that the energy allocation models consider the opportunity cost of allocating metabolites to growth, however, they still assume that cells have a limited energy or carbon budget, which is equivalent to assuming provisioning is limiting. To emphasize this point and avoid confusion, we have provided more explanation in the introduction (lines 54-62) and discussion (lines 409-411)

Minor issues/comments (it is up to the authors if they want to address these):

1) An assumption in the energetic cost models is that the relative energetic cost scales linearly with the fitness penalty (equal to the relative depression in growth rate if differences in survival are not relevant). A proteome allocation model as presented here could be used to test this assumption. This is already mentioned in the text in the discussion, e.g. lines 354-373. However, this only examines maintenance costs. To examine the effect of what in the energy allocation models is called growth or construction cost (mostly opportunity costs) on fitness one would have to examine the effect of a change in the structural fraction (from a starting point of ϕ_S=0). When I examine this using Eq 8 it appears that there is a linear relation between ϕ_S and the birth rate under optimal allocation. But this is for the Taylor expansion (Eq 8) and I'm curious to see if the full model yields the same linear result. It would be interesting to see a plot of birth rate as a function of the structural fraction. Examining the effect of SM in the same way reveals that its relationship with ϕ_S is more complicated (as was already mentioned by the authors in the discussion).

The reviewer raises an important question regarding whether the costs are linear or not. To address this point, we now have included the expression for birth rate before the Taylor expansion (after line 255). In addition, we have added a panel to Figure 4

(panel c, explained on lines 322-327), which explicitly describes conditions under which the costs are linear or nonlinear, which clarifies this matter.

2) For the claim in the abstract that "increasing food quality increases the cost of traits that require the production of new structures", would it still be true if a relative birth rate is examined rather than an absolute one.

The reviewer is right that exploring relative cost is more relevant in models of selection in discrete time (e.g., W_A/W_a). However, the models in continuous time focus on differences in growth rates, because these do correspond to relative fitness in discrete-time models. That is, $e^{(b_A-b_a)t}$ is equivalent to W_A/W_a if t is taken as a cell generation. We have clarified this point on lines 280-284.

3) On lines 138 and 139 it is stated that "As the biosynthesis fraction increases, there are fewer provisioning enzymes to supply each biosynthetic enzyme, and the biosynthesis saturation level decreases". Can't a small provisioning fraction with a large efficiency not supply enough substrates to saturate biosynthesis?

We appreciate the reviewer's point and agree that when the biosynthesis fraction is saturated, the reduction in saturation level upon decreasing the provisioning fraction will be small. Nonetheless, there will be some reduction. To increase clarity, we have changed the end of sentence to "and the biosynthesis saturation level decreases at an accelerating rate" on line 148

4) One wonders if we should expect a hyperbolic function relating the substrate concentration to the biosynthesis rate since there are multiple steps between an amino acid just having been produced and protein chain elongation (i.e. tRNA charging, elongation factor binding, and ternary complex binding to ribosomes). My suspicion is that the assumption of a hyperbolic relation is fine because even whole cell growth rate is related to external nutrient concentration by a hyperbolic function.

We agree with the reviewer that hyperbolic relation is a reasonable first-order approximation for a wide variety of biological systems. Substituting a more specific relationship can be done based on the specific system of interest, such as considered in the appendix by explicitly tracking substrate concentrations.

5) The level of activity of the biosynthesis fraction is affected by the substrate pool, and thereby by the provisioning fraction. However, the reverse could also be true that removal of substrates by the biosynthesis fraction could increase the flux through the provisioning fraction and thereby make the provisioning fraction more efficient. This does not need to be addressed here.

We agree with the reviewer that this can be a direction for future studies.

6) I want to draw attention to some literature that deals with similar questions of multiple or other resource types (or constraints). It is up to the authors whether they want to discuss any of this in their paper, but it does present some other directions that the approach of the authors could be extended into in the future. John Raven has written many articles on cost-benefit analyses in which both energy and specific elemental resources are examined though not combined into a single fitness metric (Raven (1987) New Phytol; Raven (2011) Physiol Plant; Raven and Richardson (1984) New Phytol). There is also work on the effects of cell surface area occupation: (Szenk, Dill, and de Graff (2017) Cell Syst; Zhuang, Vemuri, and Mahadevan (2011) Mol Syst Biol) and on the combination of cell surface area and the energetic approach in one fitness calculation (Schavemaker and Lynch (2025) Cell Reports).

We thank the reviewer for providing this literature, particularly the work by Raven which is new to us. We now discuss the membrane surface area limitation as one of the limitations of our model on lines 437-438.

7) The ratio kB/ has a significant impact on the birth rate and, by using the approximation, is essentially set to 1 in the model in the main text, even though the discussion in the supplement suggests the real value of kB/ to be on the order of 0.1. This makes the model not directly applicable to fitness calculations, which is unfortunate but doesn't negate the advances made in this work. This can be addressed in subsequent work by people interested in applying the model to specific biological questions.

We agree with the reviewer that this ratio can affect the quantitative predictions. As this ratio can be different for various systems, future studies can use the model provided in S4 Text to tailor the predictions to their system of interest.

8) It may be useful to plot the findings of Fig 4 not just as an absolute change in birth rate but also as a relative change in birth rate, as that is more relevant to evolutionary questions.

We agree with the reviewer that it would be interesting to include plots of relative change, but as the absolute difference in birth rate in continuous time models correspond to relative fitness in discrete time models, we decided to not include that graph.

9) The terms ”environmental quality” and ”genetic background” could use some elaboration with biological examples in the introduction.

We appreciated the reviewer's comment and provided examples in the introduction on line 42.

10) Shouldn't ”predicted” in line 313 be ”observed”?

We realized that sentence is written in a confusing way and edited it to improve clarity (lines 363-364).

11) In the text it is stated that some of the lines in Fig 5a have a non-zero intercept (lines 308-310). This is not obvious from Fig 5a, so it may be helpful to include a zoomed version of Fig 5a at the low birthrate region of the plot that shows the intercepts.

We appreciated the reviewer's comment and now include a zoomed-in version at low birth rates in Fig 5a.

12) The tick labels on some of the axes could be bigger to increase legibility (Fig 3b-f, Fig 4, and Fig 5).

We appreciated the reviewer's comment and made that adjustment in Figures 4 and 5. However, we did not change Figs 3b-3f as these have the same scale as Fig 3a (as discussed in Figure caption) and are only meant to emphasize qualitative changes.

13) In the equation that gives Sm in the ”Maintenance demands” section of the supplement there is a typo in Avogadro's constant. (Note also that the line numbering is absent in this section.)

We appreciated the reviewer's attention to this issue and fixed it.

14) In the supplement, line 83 has a typo in the equation. It says ”vollume” instead of ”volume”.

We appreciated the reviewer's attention to this issue and fixed it.

15) In the supplement, line 124 states "the effect" twice.

We appreciated the reviewer's attention to this issue and fixed it.

2.2 Reviewer #2

This manuscript proposes a unified macromolecule allocation framework that simultaneously considers energy provisioning and biosynthesis limitations. Addressing this problem is highly relevant and could represent an important contribution to ecological theory. The text is well written and generally clear.

The following are my comments to the authors:

I appreciate the authors' effort to produce a unified framework to understand the cost of diverse traits and to explain observed patterns of bacterial growth. In my view, quantifying cost as an absolute reduction in the birth rate of cells is appropriate, as growth rate is one of the most broadly used measures of Darwinian fitness. However, a crucial assumption in the optimal allocation analysis (fig 3), cost of traits analysis (fig 4), and the bacterial growth laws (fig 5) is that the structural fraction is constant. The manuscript does not present evidence that this assumption holds across environmental conditions or genotypes. Because deviations from this assumption could bias optimal allocations, trait cost predictions, and the correspondence with growth laws, the authors should either provide empirical support for this assumption or explicitly acknowledge this limitation in the discussion.

We appreciated the reviewer's comment and discussed this limitation both in the model section (lines 176-181) and discussion (lines 432-434).

Another concern is that the framework appears to be formulated primarily for high-quality environments in which food has a stoichiometric composition similar to that of the cells. However, in low-quality environments, for example, those with high carbon availability (e.g., sugars) but limited phosphorus or nitrogen, stoichiometric imbalances could influence how macromolecules are allocated between energy provisioning and biosynthesis. The authors partially mention this limitation in the supplementary material when

discussing assumptions of parameter independence. However, I consider this a central limitation of the model that should be discussed in the main text. For this reason, I also find the claim that the model facilitates a synthesis between ecological stoichiometry theory and the metabolic theory of ecology (L349-353) somewhat overstated. Such a synthesis may be better framed as a direction for future work rather than a conclusion of the present study.

We appreciated the reviewer's comment and discussed this limitation in the model section (lines 181-190) and discussion (lines 433-434). Moreover, we framed the synthesis with ecological stoichiometry as a direction for future studies, as suggested by the reviewer (lines 403-406).

Despite these limitations, I value the contribution of this framework. Scientific progress often occurs incrementally, and clearly acknowledging model limitations can help guide future work to address them. In this sense, the present study advances the field by building upon the framework of Scott et al. (2010).
