# Supplementary material for: Bridging energy and proteome allocation models allows prediction of the resource cost of traits across environments
Source: PLoS Biol. 2026 Oct 13;24(10):e3003995. doi: 10.1371/journal.pbio.3003995 (PMC13660896; doi:10.1371/journal.pbio.3003995)

# Bridging energy and proteome allocation models allows prediction of the resource cost of traits across environments

In[∘]:= assumptions =

$$\{\kappa_B \geq 0,\ \rho \geq 0,\ 0 \leq \phi_S \leq 1,\ 0 \leq \phi_B \leq 1,\ 0 \leq \phi_P \leq 1,\ \phi_S + \phi_B + \phi_P == 1,\ S_M \geq 0,\ D \geq 0,\ 0 \leq c \leq 1,$$
$$\epsilon_B > 0,\ \epsilon_P > 0,\ M_{Total} \geq 0,\ \epsilon_P\,(1 - \phi_S) - S_M + c\,D \geq 0,\ \epsilon_P\,(1 - \phi_S) - S_M \geq 0,\ \alpha \geq 0,\ \delta \geq 0\};$$

## Results in the main text

### Derivation of equations 3 to 9

According to equations 1 and 2, the birth rate equals:

In[∘]:= saturation =

$$\frac{1}{1 + \frac{\epsilon_B\,\phi_B - D}{\epsilon_P\,\phi_P - S_M + c\,D}}\ /.\ \phi_P \to 1 - \phi_B - \phi_S;$$

In[∘]:= birth =

$$\phi_B\,\epsilon_B\,\frac{1}{1 + \frac{\epsilon_B\,\phi_B - D}{\epsilon_P\,\phi_P - S_M + c\,D}} - D\ /.\ \phi_P \to 1 - \phi_B - \phi_S;$$

The optimal size of the biosynthesis fraction equals:

In[∘]:= solutions = Simplify[Solve[D[birth, $\phi_B$] == 0, $\phi_B$], assumptions]

Out[∘]=

$$\Big\{\Big\{\phi_B \to \frac{1}{(\epsilon_B - \epsilon_P)\,\epsilon_P}\Big((D - c\,D + S_M)\,\epsilon_P + \sqrt{\epsilon_P\,(D\,\epsilon_P + \epsilon_B\,(-c\,D + S_M + \epsilon_P\,(-1 + \phi_S)))\,(D - c\,D + S_M + \epsilon_P\,(-1 + \phi_S))} + \epsilon_P^2\,(-1 + \phi_S)\Big)\Big\},\ \Big\{\phi_B \to \frac{1}{(\epsilon_B - \epsilon_P)\,\epsilon_P}\Big((D - c\,D + S_M)\,\epsilon_P - \sqrt{\epsilon_P\,(D\,\epsilon_P + \epsilon_B\,(-c\,D + S_M + \epsilon_P\,(-1 + \phi_S)))\,(D - c\,D + S_M + \epsilon_P\,(-1 + \phi_S))} + \epsilon_P^2\,(-1 + \phi_S)\Big)\Big\}\Big\}$$

In[∘]:= solutions /. $\{\epsilon_P \to 1.1,\ \epsilon_B \to 10,\ \phi_S \to 0.1,\ c \to 1,\ D \to 2,\ S_M \to 0.1\}$

Out[∘]=

$$\{\{\phi_B \to 0.422233\},\ \{\phi_B \to -0.622233\}\}$$

We need the larger root that is positive:

$$\phi_B \to \frac{(D-c\,D+S_M)\,\epsilon_P+\sqrt{\epsilon_P\,(D\,\epsilon_P+\epsilon_B\,(-c\,D+S_M+\epsilon_P\,(-1+\phi_S)))\,(D-c\,D+S_M+\epsilon_P\,(-1+\phi_S))}+\epsilon_P^2\,(-1+\phi_S)}{(\epsilon_B-\epsilon_P)\,\epsilon_P}$$

## Optimal biosynthesis fraction

We define $\delta = \frac{\epsilon_B}{\epsilon_P}$ and $\alpha = \epsilon_P\,(1-\phi_S) + c\,D - S_M$.

In[ ]:=
```
FullSimplify[solutions[[1]] /. {ϵB → δ ϵP, SM → ϵP (1 - ϕS) + c D - α}, assumptions]
```

Out[ ]=

$$\left\{\phi_B \to \frac{D-\alpha+\sqrt{(D-\alpha)\,(D-\alpha\,\delta)}}{(-1+\delta)\,\epsilon_P}\right\}$$

In[ ]:=
```
biosynthesisOptimal = Simplify[% /. {α → ϵP (1 - ϕS) - SM + c D}, assumptions]
```

Out[ ]=

$$\left\{\phi_B \to \frac{D-c\,D+S_M-\epsilon_P+\sqrt{(D-c\,D+S_M+\epsilon_P\,(-1+\phi_S))\,(D-c\,D\,\delta+\delta\,S_M+\delta\,\epsilon_P\,(-1+\phi_S))}+\epsilon_P\,\phi_S}{(-1+\delta)\,\epsilon_P}\right\}$$

Taylor series expansion:

In[ ]:=
```
biosynthesisOptimalTaylor1 = Simplify[Series[ϕB /. %, {D, 0, 2}], assumptions]
```

Out[ ]=

$$-\frac{S_M+\epsilon_P\,(-1+\phi_S)}{\left(1+\sqrt{\delta}\right)\epsilon_P}-\frac{\left(1-2\,c-\frac{1}{\sqrt{\delta}}\right)D}{2\left(\left(1+\sqrt{\delta}\right)\epsilon_P\right)}+\frac{(-1+\delta)\,D^2}{8\,\delta^{3/2}\,\epsilon_P\,(S_M+\epsilon_P\,(-1+\phi_S))}+O[D]^3$$

## Saturation under optimal allocation

In[ ]:=
```
FullSimplify[saturation /. {biosynthesisOptimal} /.
  {ϵB → δ ϵP, SM → ϵP (1 - ϕS) + c D - α }, assumptions]
```

Out[ ]=

$$\left\{\frac{\alpha}{-D+\alpha+\sqrt{(D-\alpha)\,(D-\alpha\,\delta)}}\right\}$$

In[ ]:=
```
saturationOptimal = Simplify[% /. {α → ϵP (1 - ϕS) - SM + c D}, assumptions]
```

Out[ ]=

$$\left\{-\frac{c\,D-S_M-\epsilon_P\,(-1+\phi_S)}{D-c\,D+S_M-\sqrt{(D-c\,D+S_M+\epsilon_P\,(-1+\phi_S))\,(D-c\,D\,\delta+\delta\,S_M+\delta\,\epsilon_P\,(-1+\phi_S))}+\epsilon_P\,(-1+\phi_S)}\right\}$$

Taylor series expansion:

In[ ]:=
```
saturationOptimalTaylor1 = Simplify[Series[%, {D, 0, 1}], assumptions]
```

Out[ ]=

$$\left\{\frac{1}{1+\sqrt{\delta}}+\frac{D}{2\,\sqrt{\delta}\,(S_M+\epsilon_P\,(-1+\phi_S))^2}+O[D]^2\right\}$$

## Birth rate under optimal allocation

In[•]:=
```
FullSimplify[
 birth /. {biosynthesisOptimal[[1]]} /. {ϵB → δ ϵP, SM → ϵP (1 - ϕS) + c D - α }, assumptions]
```

Out[•]=

$$\frac{-D\left(1+\delta^2\right)+\delta\left(\alpha+\alpha\,\delta-2\sqrt{(D-\alpha)\,(D-\alpha\,\delta)}\right)}{(-1+\delta)^2}$$

In[•]:=
```
birthOptimal = Simplify[% /. {α → ϵP (1 - ϕS) - SM + c D}, assumptions]
```

Out[•]=

$$-\frac{1}{(-1+\delta)^2}\Big(D-c\,D\,\delta+D\,\delta^2-c\,D\,\delta^2+\delta\,(1+\delta)\,S_M+\delta\,(1+\delta)\,\epsilon_P\,(-1+\phi_S)+2\,\delta\sqrt{((-1+c)\,D-S_M-\epsilon_P\,(-1+\phi_S))\,(D\,(-1+c\,\delta)-\delta\,S_M+\epsilon_P\,(\delta-\delta\,\phi_S))}\Big)$$

If degradation rate is small, we can use Taylor series to simplify this

In[•]:=
```
birthOptimalTaylor1 = Simplify[Series[%, {D, 0, 1}],  assumptions] // Normal
```

Out[•]=

$$-\frac{D\left(1+\sqrt{\delta}+\delta-c\,\delta\right)}{\left(1+\sqrt{\delta}\right)^2}-\frac{\delta\,(S_M+\epsilon_P\,(-1+\phi_S))}{\left(1+\sqrt{\delta}\right)^2}$$

We then rearrange as follows:

$$\frac{\delta\,(\epsilon_P\,(1-\phi_S))}{\left(1+\sqrt{\delta}\right)^2}-\frac{\delta\,S_M}{\left(1+\sqrt{\delta}\right)^2}-\frac{D\left(1+\sqrt{\delta}+(1-c)\,\delta\right)}{\left(1+\sqrt{\delta}\right)^2}$$

$$=\frac{\delta\,(\epsilon_P\,(1-\phi_S))}{\left(1+\sqrt{\delta}\right)^2}-\frac{\delta\,S_M}{\left(1+\sqrt{\delta}\right)^2}-\frac{D\left(1+2\sqrt{\delta}-\sqrt{\delta}+(1-c)\,\delta\right)}{\left(1+\sqrt{\delta}\right)^2}$$

$$=\frac{\delta\,(\epsilon_P\,(1-\phi_S))}{\left(1+\sqrt{\delta}\right)^2}-\frac{\delta\,S_M}{\left(1+\sqrt{\delta}\right)^2}-\frac{D\left(1+2\sqrt{\delta}+\delta\right)-D\left(\sqrt{\delta}+-c\,\delta\right)}{\left(1+\sqrt{\delta}\right)^2}$$

$$=\frac{\delta\,(\epsilon_P\,(1-\phi_S))}{\left(1+\sqrt{\delta}\right)^2}-\frac{\delta\,S_M}{\left(1+\sqrt{\delta}\right)^2}-D\left(1-\frac{\sqrt{\delta}+c\,\delta}{\left(1+\sqrt{\delta}\right)^2}\right)$$

## Derivation of the intercept of growth laws (the biosynthesis fraction and saturation level when the growth rate approaches zero)

The provisioning efficiency that results in a 0 birth rate equals

In[ ]:= 
```
Solve[birthOptimal == 0 /. δ → ϵB/ϵP, ϵP]
```

Solve: There may be values of the parameters for which some or all solutions are not valid.

Out[ ]=

$$\left\{\left\{\epsilon_P \to \frac{(-D + c\,D - S_M)\,\epsilon_B}{D - \epsilon_B + \epsilon_B\,\phi_S}\right\}\right\}$$

At that provisioning efficiency, the biosynthesis fraction equals $\frac{D}{\epsilon_B}$ and the biosynthesis saturation level is maximal:

In[ ]:= 
```
Simplify[biosynthesisOptimal /. δ → ϵB/ϵP /. ϵP → (-D + c D - SM) ϵB/(D - ϵB + ϵB ϕS), assumptions]
```

Out[ ]=

$$\left\{\phi_B \to \frac{D}{\epsilon_B}\right\}$$

In[ ]:= 
```
Simplify[saturationOptimal /. δ → ϵB/ϵP /. ϵP → (-D + c D - SM) ϵB/(D - ϵB + ϵB ϕS), assumptions]
```

Out[ ]=

{1}

However if degradation is absent, the saturation approaches $\left\{\frac{1}{1+\sqrt{-\frac{\epsilon_B\,(-1+\phi_S)}{S_M}}}\right\}$ at the provisioning efficiency that leads to zero growth

In[ ]:= 
```
Solve[birthOptimal == 0 /. δ → ϵB/ϵP /. D → 0, ϵP]
```

Out[ ]=

$$\left\{\left\{\epsilon_P \to -\frac{S_M}{-1 + \phi_S}\right\}\right\}$$

In[ ]:= 
```
FullSimplify[
 saturation /. {biosynthesisOptimal} /. {ϵB → δ ϵP, SM → ϵP (1 - ϕS) + c D - α} /. D → 0,
 assumptions]
```

Out[ ]=

$$\left\{\frac{1}{1 + \sqrt{\delta}}\right\}$$

In[ ]:= 
```
Simplify[% /. δ → ϵB/ϵP /. ϵP → -SM/(-1 + ϕS), assumptions]
```

Out[ ]=

$$\left\{\frac{1}{1 + \sqrt{-\frac{\epsilon_B\,(-1+\phi_S)}{S_M}}}\right\}$$

The biosynthesis efficiency that results in a zero growth rate equals

In[ ]:= 
```
Solve[birthOptimal == 0 /. δ → ϵB/ϵP, ϵB]
```

Solve: There may be values of the parameters for which some or all solutions are not valid.

Out[ ]=

$$\left\{\left\{\epsilon_B \to \frac{D\,\epsilon_P}{-D + c\,D - S_M + \epsilon_P - \epsilon_P\,\phi_S}\right\}\right\}$$

At that provisioning efficiency, the biosynthesis fraction and the biosynthesis saturation level equal:

In[ ]:= 
```
Simplify[biosynthesisOptimal /. δ → ϵB/ϵP /. ϵB → D ϵP/(-D + c D - SM + ϵP - ϵP ϕS), assumptions]
```

Out[ ]=

$$\left\{\phi_B \to -\frac{D - c\,D + S_M + \epsilon_P\,(-1 + \phi_S)}{\epsilon_P}\right\}$$

In[ ]:= 
```
Simplify[saturationOptimal /. δ → ϵB/ϵP /. ϵB → D ϵP/(-D + c D - SM + ϵP - ϵP ϕS), assumptions]
```

Out[ ]=

$$\{1\}$$

## Deriving the slope of the growth laws

In the absence of degradation and maintenance the slope of growth laws when calculating provisioning efficiency ($\epsilon_P$) is calculated as follows

In[ ]:= 
```
Simplify[
 Simplify[D[ϕB /. biosynthesisOptimal /. δ → ϵB/ϵP /. {D → 0, SM → 0}, ϵP], assumptions]
 / Simplify[D[birthOptimal /. δ → ϵB/ϵP /. {D → 0, SM → 0}, ϵP], assumptions],
 assumptions]
```

Out[ ]=

$$\frac{1}{2}\left(\frac{1}{\epsilon_B} + \frac{1}{\sqrt{\epsilon_B\,\epsilon_P}}\right)$$

In[ ]:= 
```
Simplify[% /. ϵB → δ ϵP, {δ > 0, ϵP > 0, ϵB > 0}] /. δ → ϵB/ϵP
```

Out[ ]=

$$\frac{1 + \sqrt{\frac{\epsilon_B}{\epsilon_P}}}{2\,\epsilon_B}$$

In the absence of degradation and maintenance the slope of growth laws when calculating biosynthesis efficiency ($\epsilon_B$) is calculated as follows

In[ ]:=
```
Simplify[
  Simplify[D[ϕB /. biosynthesisOptimal /. δ → ϵB/ϵP /. {D → 0, SM → 0}, ϵB], assumptions] /
  Simplify[D[birthOptimal /. δ → ϵB/ϵP /. {D → 0, SM → 0}, ϵB], assumptions],
  {ϵP > 0, ϵB > 0, ϵP (1 - ϕS) - SM + c D ≥ 0 ,
   ϵP (1 - ϕS) - SM ≥ 0 , ϵP (1 - ϕS) + c D ≥ SM + D, 0 < ϕS < 1}]
```

Out[ ]=

$$-\frac{(\epsilon_B-\epsilon_P)^3}{2\left(\sqrt{\epsilon_B}+\sqrt{\epsilon_P}\right)^2\sqrt{\epsilon_B\,\epsilon_P}\left(-3\,\epsilon_B\,\epsilon_P-\epsilon_P^2+\sqrt{\epsilon_B^3\,\epsilon_P}+3\sqrt{\epsilon_B\,\epsilon_P^3}\right)}$$

In[ ]:=
```
Simplify[% /. ϵB → δ ϵP, {δ > 0, ϵP > 0, ϵB > 0}] /. δ → ϵB/ϵP
```

Out[ ]=

$$-\frac{1+\frac{1}{\sqrt{\frac{\epsilon_B}{\epsilon_P}}}}{2\,\epsilon_P}$$

# Derivation of results in the supplementary material

## Explicitly modelling substrate dynamics (S1 Appendix)

Solving equation S1 for for steady state substrate concentration:

In[ ]:=
```
SubstrateSS = Solve[(ϕP ϵP + c D - SM - (ϕB ϵB)/(1 + κB/S)) - ((ϕB ϵB)/(1 + κB/S) - D) S == 0, S]
```

Solve: This system cannot be solved with the methods available to Solve. Try Reduce or FindInstance instead.

Out[ ]=

$$\text{Solve}\left[c\,D-S_M-\frac{\epsilon_B\,\phi_B}{1+\frac{\kappa_B}{S}}-S\left(-D+\frac{\epsilon_B\,\phi_B}{1+\frac{\kappa_B}{S}}\right)+\epsilon_P\,\phi_P==0,\ S\right]$$

As the equation cannot be directly solved, we define Supply = $\phi_P\ \epsilon_P + c\ D - S_M$ and ConsumptionMax = $\phi_B\ \epsilon_B$, which correspond to $\theta$ and $\beta_{Max}$ in the main text, respectively.

In[ ]:= Simplify[Solve[(((Supply - ConsumptionMax/(1 + κB/Ŝ)) - b̂ Ŝ /. b̂ → ϕB ϵB/(1 + κB/Ŝ) - D) == 0, Ŝ], {ConsumptionMax > D}]

Out[ ]=

$$\left\{\left\{\hat{S} \to -\frac{1}{2\,(D - \epsilon_B\,\phi_B)}\left(-\text{ConsumptionMax} + \text{Supply} + D\,\kappa_B + \sqrt{(-\text{ConsumptionMax} + \text{Supply} + D\,\kappa_B)^2 - 4\,\text{Supply}\,\kappa_B\,(D - \epsilon_B\,\phi_B)}\right)\right\}, \left\{\hat{S} \to \frac{1}{2\,(D - \epsilon_B\,\phi_B)}\left(\text{ConsumptionMax} - \text{Supply} - D\,\kappa_B + \sqrt{(-\text{ConsumptionMax} + \text{Supply} + D\,\kappa_B)^2 - 4\,\text{Supply}\,\kappa_B\,(D - \epsilon_B\,\phi_B)}\right)\right\}\right\}$$

In[ ]:= % /. Simplify[ConsumptionMax → Supply - temp + D κB, assumptions]

Out[ ]=

$$\left\{\left\{\hat{S} \to -\frac{\text{temp} + \sqrt{\text{temp}^2 - 4\,\text{Supply}\,\kappa_B\,(D - \epsilon_B\,\phi_B)}}{2\,(D - \epsilon_B\,\phi_B)}\right\}, \left\{\hat{S} \to \frac{-\text{temp} + \sqrt{\text{temp}^2 - 4\,\text{Supply}\,\kappa_B\,(D - \epsilon_B\,\phi_B)}}{2\,(D - \epsilon_B\,\phi_B)}\right\}\right\}$$

We need the larger root that is positive: $\frac{\text{temp}+\sqrt{\text{temp}^2-4\,\text{Supply}\,\kappa_B\,(D-\epsilon_B\,\varphi_B)}}{2\,(\epsilon_B\,\varphi_B-D)}$

In[ ]:= Clear[SubstrateSS];
SubstrateSS =
(temp + √(temp² - 4 Supply κB (D - ϵB ϕB)))/(2 (ϵB ϕB - D)) /. temp → Supply - ConsumptionMax + D κB /.
{ConsumptionMax → ϕB ϵB ρ, Supply → (ϕP ϵP + c D - SM) ρ} /. ϕP → 1 - ϕS - ϕB

Out[ ]=

$$\frac{1}{2\,(-D + \epsilon_B\,\phi_B)}\left(D\,\kappa_B - \rho\,\epsilon_B\,\phi_B + \sqrt{(D\,\kappa_B - \rho\,\epsilon_B\,\phi_B + \rho\,(c\,D - S_M + \epsilon_P\,(1 - \phi_B - \phi_S)))^2 - 4\,\rho\,\kappa_B\,(D - \epsilon_B\,\phi_B)\,(c\,D - S_M + \epsilon_P\,(1 - \phi_B - \phi_S))} + \rho\,(c\,D - S_M + \epsilon_P\,(1 - \phi_B - \phi_S))\right)$$

The following plot shows the steady state substrate concentration as a function of the biosynthesis fraction;

In[∘]:=
$$\text{Plot}[\text{SubstrateSS} \;/.\; \{\text{ConsumptionMax} \to \phi_B\,\epsilon_B,\ \text{Supply} \to \epsilon_P\,\phi_P + c\,D - S_M\} \;/.\; \phi_P \to 1 - \phi_S - \phi_B \;/.\; \kappa_B \to 0.9 \;/.\; \phi_S \to 0.1 \;/.\; S_M \to 0.1 \;/.\; D \to 0.1 \;/.\; c \to 1 \;/.\; \epsilon_B \to 3 \;/.\; \epsilon_P \to 2 \;/.\; \rho \to 2,\ \{\phi_B, 0, 1\}]$$

Out[∘]=

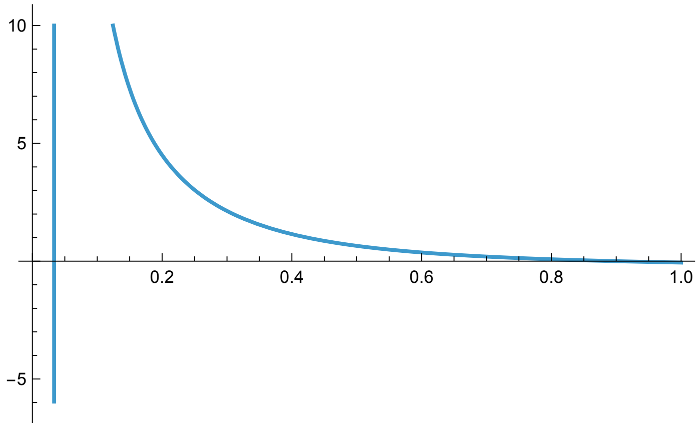


The biosynthesis saturation level equals:

In[∘]:=
$$\text{Simplify}\left[\frac{1}{1+\frac{\kappa_B}{\text{SubstrateSS}}}\right]$$

Out[∘]=
$$1\Big/\left(1-(2\,\kappa_B\,(D-\epsilon_B\,\phi_B))\Big/\left(c\,D\,\rho-\rho\,S_M+\rho\,\epsilon_P+D\,\kappa_B-\rho\,\epsilon_B\,\phi_B-\rho\,\epsilon_P\,\phi_B-\rho\,\epsilon_P\,\phi_S+\sqrt{\left(-4\,\rho\,\kappa_B\,(D-\epsilon_B\,\phi_B)\,(c\,D-S_M-\epsilon_P\,(-1+\phi_B+\phi_S))+(-c\,D\,\rho+\rho\,S_M-D\,\kappa_B+\rho\,\epsilon_B\,\phi_B+\rho\,\epsilon_P\,(-1+\phi_B+\phi_S))^2\right)}\right)\right)$$

Assuming $\kappa_B = \rho$, this simplifies to:

In[∘]:=
$$\text{Simplify}[\%\;/.\;\kappa_B \to \rho,\ \text{assumptions}]$$

Out[∘]=
$$\begin{cases} \text{ComplexInfinity} & c\,D+\epsilon_P\,\phi_P \le D+S_M+\epsilon_B\,(-1+\phi_P+\phi_S) \\ -\frac{c\,D-S_M+\epsilon_P\,\phi_P}{D-c\,D+S_M-\epsilon_P\,\phi_P+\epsilon_B\,(-1+\phi_P+\phi_S)} & \text{True} \end{cases}$$

$c\,D + \epsilon_B\,\phi_B \le D + S_M + \epsilon_P\,(-1 + \phi_B + \phi_S)$ can be rearranged to $c\,D + \epsilon_B\,\phi_B + \epsilon_P\,(1 - \phi_B - \phi_S) \le D + S_M$, which is always false under realistic conditions. Therefore biosynthesis saturation equals $-\frac{c\,D-S_M+\epsilon_P\,\phi_P}{D-c\,D+S_M-\epsilon_P\,\phi_P+\epsilon_B\,(-1+\phi_P+\phi_S)}$, which can be rearranged to: $\frac{1}{1+\frac{-D+\epsilon_B\,\phi_B}{c\,D-S_M+\epsilon_P\,(1-\phi_B-\phi_S)}}$

We next turn into calculating the birth rate, optimal biosynthesis fraction, and birth rate under the optimal allocation:

In[∘]:=
$$\text{Simplify}\left[\frac{\hat{S}}{\hat{S}+\kappa_B}\,\phi_B\,\epsilon_B - D \;/.\; \hat{S} \to \frac{\text{temp}+\sqrt{\text{temp}^2-4\,\text{Supply}\,\kappa_B\,(D-\epsilon_B\,\phi_B)}}{2\,(\epsilon_B\,\phi_B-D)},\ \text{assumptions}\right]$$

Out[∘]=
$$-\frac{(D-\epsilon_B\,\phi_B)\left(\text{temp}-2\,D\,\kappa_B+\sqrt{\text{temp}^2-4\,\text{Supply}\,\kappa_B\,(D-\epsilon_B\,\phi_B)}\right)}{\text{temp}-2\,\kappa_B\,(D-\epsilon_B\,\phi_B)+\sqrt{\text{temp}^2-4\,\text{Supply}\,\kappa_B\,(D-\epsilon_B\,\phi_B)}}$$

In[∘]:=

$$\text{Clear[birthExact];}$$

$$\text{birthExact} = -\frac{(D - \epsilon_B\,\phi_B)\left(\text{temp} - 2\,D\,\kappa_B + \sqrt{\text{temp}^2 - 4\,\text{Supply}\,\kappa_B\,(D - \epsilon_B\,\phi_B)}\right)}{\text{temp} - 2\,\kappa_B\,(D - \epsilon_B\,\phi_B) + \sqrt{\text{temp}^2 - 4\,\text{Supply}\,\kappa_B\,(D - \epsilon_B\,\phi_B)}}\ /.$$

$$\text{temp} \to \text{Supply} - \text{ConsumptionMax} + D\,\kappa_B\ /.$$

$$\{\text{ConsumptionMax} \to \phi_B\,\epsilon_B\,\rho,\ \text{Supply} \to (\phi_P\,\epsilon_P + c\,D - S_M)\,\rho\}\ /.\ \phi_P \to 1 - \phi_S - \phi_B$$

Out[∘]=

$$-\left(\left((D - \epsilon_B\,\phi_B)\left(-D\,\kappa_B - \rho\,\epsilon_B\,\phi_B + \sqrt{\left(D\,\kappa_B - \rho\,\epsilon_B\,\phi_B + \rho\,(c\,D - S_M + \epsilon_P\,(1 - \phi_B - \phi_S))\right)^2 - 4\,\rho\,\kappa_B\,(D - \epsilon_B\,\phi_B)\,(c\,D - S_M + \epsilon_P\,(1 - \phi_B - \phi_S))} + \rho\,(c\,D - S_M + \epsilon_P\,(1 - \phi_B - \phi_S))\right)\right) \Big/ \left(D\,\kappa_B - \rho\,\epsilon_B\,\phi_B - 2\,\kappa_B\,(D - \epsilon_B\,\phi_B) + \sqrt{\left(D\,\kappa_B - \rho\,\epsilon_B\,\phi_B + \rho\,(c\,D - S_M + \epsilon_P\,(1 - \phi_B - \phi_S))\right)^2 - 4\,\rho\,\kappa_B\,(D - \epsilon_B\,\phi_B)\,(c\,D - S_M + \epsilon_P\,(1 - \phi_B - \phi_S))} + \rho\,(c\,D - S_M + \epsilon_P\,(1 - \phi_B - \phi_S))\right)\right)$$

In[∘]:=

$$\text{BiosynthesisOptimalExact} = \text{Simplify[Solve[}$$

$$\text{D[birthExact /. temp} \to \text{Supply} - \text{ConsumptionMax} + D\,\kappa_B\ /.\ \{\text{ConsumptionMax} \to \phi_B\,\epsilon_B\,\rho,$$

$$\text{Supply} \to (\phi_P\,\epsilon_P + c\,D - S_M)\,\rho\}\ /.\ \phi_P \to 1 - \phi_S - \phi_B\ ,\ \phi_B] == 0,\ \phi_B],\ \text{assumptions]}$$

••• Solve: There may be values of the parameters for which some or all solutions are not valid.

Out[∘]=

$$\Big\{\Big\{\phi_B \to \Big(c\,D\,\rho\,\epsilon_P^2 - \rho\,S_M\,\epsilon_P^2 + \rho\,\epsilon_P^3 - D\,\epsilon_P^2\,\kappa_B + \text{Abs}[\epsilon_B - \epsilon_P]\,\sqrt{\left(\epsilon_P\,\kappa_B\left(D\,\epsilon_P\,(\rho\,S_M + D\,(-c\,\rho + \kappa_B) + \rho\,\epsilon_P\,(-1 + \phi_S)) + \rho\,\epsilon_B\left((-1 + c)\,c\,D^2 + S_M^2 + S_M\,(D - 2\,c\,D + 2\,\epsilon_P\,(-1 + \phi_S)) - (-1 + 2\,c)\,D\,\epsilon_P\,(-1 + \phi_S) + \epsilon_P^2\,(-1 + \phi_S)^2\right)\right)\right)} + \epsilon_B\,\epsilon_P\,(-S_M\,(\rho - 2\,\kappa_B) + D\,(c\,\rho + \kappa_B - 2\,c\,\kappa_B) - \epsilon_P\,(\rho - 2\,\kappa_B)\,(-1 + \phi_S)) - \rho\,\epsilon_P^3\,\phi_S\Big) \Big/ \left(\epsilon_P\left(\rho\,\epsilon_B^2 + \rho\,\epsilon_P^2 + 2\,\epsilon_B\,\epsilon_P\,(\rho - 2\,\kappa_B)\right)\right)\Big\},$$

$$\Big\{\phi_B \to \Big(c\,D\,\rho\,\epsilon_P^2 - \rho\,S_M\,\epsilon_P^2 + \rho\,\epsilon_P^3 - D\,\epsilon_P^2\,\kappa_B - \text{Abs}[\epsilon_B - \epsilon_P]\,\sqrt{\left(\epsilon_P\,\kappa_B\left(D\,\epsilon_P\,(\rho\,S_M + D\,(-c\,\rho + \kappa_B) + \rho\,\epsilon_P\,(-1 + \phi_S)) + \rho\,\epsilon_B\left((-1 + c)\,c\,D^2 + S_M^2 + S_M\,(D - 2\,c\,D + 2\,\epsilon_P\,(-1 + \phi_S)) - (-1 + 2\,c)\,D\,\epsilon_P\,(-1 + \phi_S) + \epsilon_P^2\,(-1 + \phi_S)^2\right)\right)\right)} + \epsilon_B\,\epsilon_P\,(-S_M\,(\rho - 2\,\kappa_B) + D\,(c\,\rho + \kappa_B - 2\,c\,\kappa_B) - \epsilon_P\,(\rho - 2\,\kappa_B)\,(-1 + \phi_S)) - \rho\,\epsilon_P^3\,\phi_S\Big) \Big/ \left(\epsilon_P\left(\rho\,\epsilon_B^2 + \rho\,\epsilon_P^2 + 2\,\epsilon_B\,\epsilon_P\,(\rho - 2\,\kappa_B)\right)\right)\Big\}\Big\}$$

In[ ]:=
```
useparameters = {ϵB → 2, ϵP → 1, c → 0.8, ϕS → 0.5, D → 0.01, SM → 0.005, ϰB → 1, ρ → 10};
Show[
 Plot[birthExact /. useparameters, {ϕB, 0, 1}],
 ListPlot[
  {{ϕB /. BiosynthesisOptimalExact[[1]], 0}, {ϕB /. BiosynthesisOptimalExact[[1]], 1}} /.
   useparameters, PlotStyle → ■, Joined → True],
 ListPlot[
  {{ϕB /. BiosynthesisOptimalExact[[2]], 0}, {ϕB /. BiosynthesisOptimalExact[[2]], 1}} /.
   useparameters, PlotStyle → ■, Joined → True],
 PlotRange → {{0, 1}, {0, 1}}, AxesLabel → {"ϕB", "birth rate"}]
```

Out[ ]=

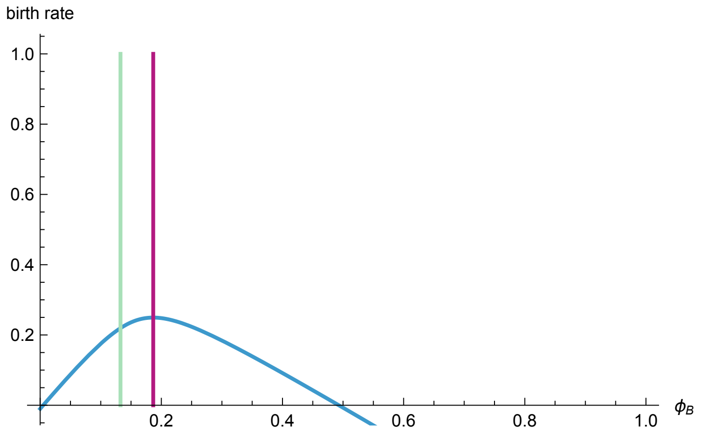

In[ ]:=
```
useparameters = {ϵB → 2, ϵP → 4, c → 0.8, ϕS → 0.5, D → 0.01, SM → 0.005, κB → 1, ρ → 10};
Show[
 Plot[birthExact /. useparameters, {ϕB, 0, 1}],
 ListPlot[
  {{ϕB /. BiosynthesisOptimalExact〚1〛, 0}, {ϕB /. BiosynthesisOptimalExact〚1〛, 1}} /.
   useparameters, PlotStyle → ■, Joined → True],
 ListPlot[
  {{ϕB /. BiosynthesisOptimalExact〚2〛, 0}, {ϕB /. BiosynthesisOptimalExact〚2〛, 1}} /.
   useparameters, PlotStyle → ■, Joined → True],
 PlotRange → {{0, 1}, {0, 1}}, AxesLabel → {"ϕB", "birth rate"}]
```

Out[ ]=

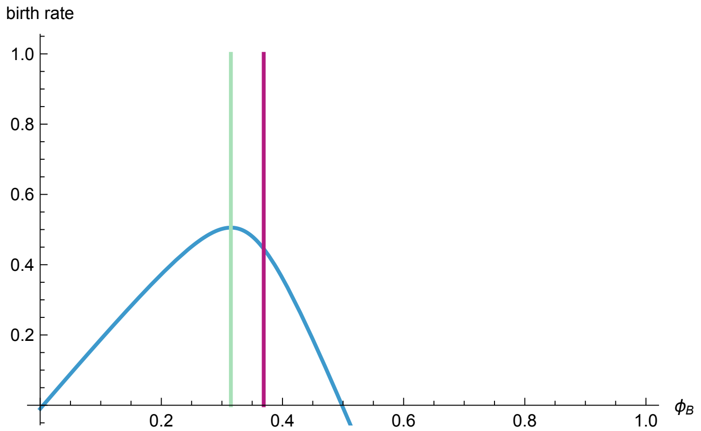


By exploring the parameter space, we find that when $\epsilon_B > \epsilon_P$, the first solution is relevant and when $\epsilon_B < \epsilon_P$, the second solutions is relevant. Therefore the optimal allocation always equals (notice the sign of Abs[$\epsilon_B - \epsilon_P$] in previous solutions):

$$\Big\{\phi_B \to \frac{1}{\epsilon_P\left(\rho\,\epsilon_B^2+\rho\,\epsilon_P^2+2\,\epsilon_B\,\epsilon_P\,(\rho-2\,\kappa_B)\right)}\Big(c\,D\,\rho\,\epsilon_P^2-\rho\,S_M\,\epsilon_P^2+\rho\,\epsilon_P^3-D\,\epsilon_P^2\,\kappa_B+(\epsilon_B-\epsilon_P)\sqrt{\Big(\epsilon_P\,\kappa_B\,\big(D\,\epsilon_P\,(\rho\,S_M+D\,(-c\,\rho+\kappa_B)+\rho\,\epsilon_P\,(-1+\phi_S))+\rho\,\epsilon_B\,\big((-1+c)\,c\,D^2+S_M^2+S_M\,(D-2\,c\,D+2\,\epsilon_P\,(-1+\phi_S))-(-1+2\,c)\,D\,\epsilon_P\,(-1+\phi_S)+\epsilon_P^2\,(-1+\phi_S)^2\big)\big)\Big)}+\epsilon_B\,\epsilon_P\,(-S_M\,(\rho-2\,\kappa_B)+D\,(c\,\rho+\kappa_B-2\,c\,\kappa_B)-\epsilon_P\,(\rho-2\,\kappa_B)\,(-1+\phi_S))-\rho\,\epsilon_P^3\,\phi_S\Big)\Big\}$$

In[•]:=
```
Clear[BiosynthesisOptimalExactRelevant];
BiosynthesisOptimalExactRelevant[ϵP_, ϵB_, ϕS_, c_, D_, SM_, κB_, ρ_] :=
 BiosynthesisOptimalExactRelevant[ϵP, ϵB, ϕS, c, D, SM, κB, ρ] =
```

$$\frac{1}{\epsilon_P\left(\rho\,\epsilon_B^2+\rho\,\epsilon_P^2+2\,\epsilon_B\,\epsilon_P\,(\rho-2\,\kappa_B)\right)}\Big(c\,D\,\rho\,\epsilon_P^2-\rho\,S_M\,\epsilon_P^2+\rho\,\epsilon_P^3-D\,\epsilon_P^2\,\kappa_B+(\epsilon_B-\epsilon_P)\sqrt{\Big(\epsilon_P\,\kappa_B\,\big(D\,\epsilon_P\,(\rho\,S_M+D\,(-c\,\rho+\kappa_B)+\rho\,\epsilon_P\,(-1+\phi_S))+\rho\,\epsilon_B\,\big((-1+c)\,c\,D^2+S_M^2+S_M\,(D-2\,c\,D+2\,\epsilon_P\,(-1+\phi_S))-(-1+2\,c)\,D\,\epsilon_P\,(-1+\phi_S)+\epsilon_P^2\,(-1+\phi_S)^2\big)\big)\Big)}+\epsilon_B\,\epsilon_P\,(-S_M\,(\rho-2\,\kappa_B)+D\,(c\,\rho+\kappa_B-2\,c\,\kappa_B)-\epsilon_P\,(\rho-2\,\kappa_B)\,(-1+\phi_S))-\rho\,\epsilon_P^3\,\phi_S\Big)\ /.$$

```
{ϵB → ϵB , ϵP → ϵP, ϕS → ϕS , SM → SM, κB → κB}
```

In[•]:=
```
BiosynthesisOptimalExactRelevant[1, 2, 0.5, 0.85, 0.01, 0.05, 1, 1]
```

Out[•]=
```
0.189301
```

In[•]:=
```
Clear[SaturationOptimalExact];
SaturationOptimalExact[ϵP_, ϵB_, ϕS_, c_, D_, SM_, κB_, ρ_] :=
 SaturationOptimalExact[ϵP, ϵB, ϕS, c, D, SM, κB, ρ] =
```

$$\frac{1}{1+\frac{\kappa_B}{\hat{S}}}\ /.\ \hat{S}\to-\frac{1}{2\,(D-\epsilon_B\,\phi_B)}\Big(D\,\kappa_B-\rho\,\epsilon_B\,\phi_B+\sqrt{\big((D\,\kappa_B-\rho\,\epsilon_B\,\phi_B+\rho\,(c\,D-S_M+\epsilon_P\,(1-\phi_B-\phi_S)))^2-4\,\rho\,\kappa_B\,(D-\epsilon_B\,\phi_B)\,(c\,D-S_M+\epsilon_P\,(1-\phi_B-\phi_S))\big)}+\rho\,(c\,D-S_M+\epsilon_P\,(1-\phi_B-\phi_S))\Big)\ /.$$

```
{ϵB → ϵB , ϵP → ϵP, ϕS → ϕS , SM → SM, κB → κB} /. ϕB →
 BiosynthesisOptimalExactRelevant[ϵP, ϵB, ϕS, c, D, SM, κB, ρ]
```

In[•]:=
```
SaturationOptimalExact[1, 2, 0.5, 0.85, 0.01, 0.05, 1, 1]
```

Out[•]=
```
0.422075
```

In[•]:=
```
birthExact
```

Out[•]=

$$-\Big(\Big((D-\epsilon_B\,\phi_B)\,\Big(-D\,\kappa_B-\rho\,\epsilon_B\,\phi_B+\sqrt{\big((D\,\kappa_B-\rho\,\epsilon_B\,\phi_B+\rho\,(c\,D-S_M+\epsilon_P\,(1-\phi_B-\phi_S)))^2-4\,\rho\,\kappa_B\,(D-\epsilon_B\,\phi_B)\,(c\,D-S_M+\epsilon_P\,(1-\phi_B-\phi_S))\big)}+\rho\,(c\,D-S_M+\epsilon_P\,(1-\phi_B-\phi_S))\Big)\Big)\Big/\Big(D\,\kappa_B-\rho\,\epsilon_B\,\phi_B-2\,\kappa_B\,(D-\epsilon_B\,\phi_B)+\sqrt{\big((D\,\kappa_B-\rho\,\epsilon_B\,\phi_B+\rho\,(c\,D-S_M+\epsilon_P\,(1-\phi_B-\phi_S)))^2-4\,\rho\,\kappa_B\,(D-\epsilon_B\,\phi_B)\,(c\,D-S_M+\epsilon_P\,(1-\phi_B-\phi_S))\big)}+\rho\,(c\,D-S_M+\epsilon_P\,(1-\phi_B-\phi_S))\Big)\Big)$$

In[•]:=
```
Clear[BirthOptimalExact];
BirthOptimalExact[ϵP_, ϵB_, ϕS_, c_, D_, SM_, κB_, ρ_] :=
  BirthOptimalExact[ϵP, ϵB, ϕS, c, D, SM, κB, ρ] =
```

$$-\Big(\Big((D-\epsilon_B\,\phi_B)\,\Big(-D\,\kappa_B-\rho\,\epsilon_B\,\phi_B+\sqrt{\big((D\,\kappa_B-\rho\,\epsilon_B\,\phi_B+\rho\,(c\,D-S_M+\epsilon_P\,(1-\phi_B-\phi_S)))^2-4\,\rho\,\kappa_B\,(D-\epsilon_B\,\phi_B)\,(c\,D-S_M+\epsilon_P\,(1-\phi_B-\phi_S))\big)}+\rho\,(c\,D-S_M+\epsilon_P\,(1-\phi_B-\phi_S))\Big)\Big)\Big/\Big(D\,\kappa_B-\rho\,\epsilon_B\,\phi_B-2\,\kappa_B\,(D-\epsilon_B\,\phi_B)+\sqrt{\big((D\,\kappa_B-\rho\,\epsilon_B\,\phi_B+\rho\,(c\,D-S_M+\epsilon_P\,(1-\phi_B-\phi_S)))^2-4\,\rho\,\kappa_B\,(D-\epsilon_B\,\phi_B)\,(c\,D-S_M+\epsilon_P\,(1-\phi_B-\phi_S))\big)}+\rho\,(c\,D-S_M+\epsilon_P\,(1-\phi_B-\phi_S))\Big)\Big)\ /.$$

```
  ϕB → BiosynthesisOptimalExactRelevant[ϵP, ϵB, ϕS, c, D, SM, κB, ρ] /.
 {ϵB → ϵB , ϵP → ϵP, ϕS → ϕS , SM → SM, κB → κB};
```

In[∘]:=
```
BirthOptimalExact[1, 2, 0.5, 0.85, 0.01, 0.05, 1, 1]
```

Out[∘]=

0.149798

Under optimal allocation the steady state substrate concentration equals

In[∘]:=
```
Simplify[Simplify[SubstrateSS /. κB → ρ, assumptions] /.
  Abs[D - c D + SM - ϵP ϕP + ϵB (-1 + ϕP + ϕS)] → ϵP ϕP + ϵB (1 - ϕP - ϕS) + c D - SM - D, assumptions]
```

Out[∘]=

$$-\frac{\rho\,(c\,D - S_M + \epsilon_P\,\phi_P)}{D - \epsilon_B\,\phi_B}$$

We then plug in optimal biosynthesis fraction

In[∘]:=

$$\text{optimalBiosynthesis} = \frac{1}{\epsilon_P\left(\rho\,\epsilon_B^2 + \rho\,\epsilon_P^2 + 2\,\epsilon_B\,\epsilon_P\,(\rho - 2\,\kappa_B)\right)}\Big(c\,D\,\rho\,\epsilon_P^2 - \rho\,S_M\,\epsilon_P^2 + \rho\,\epsilon_P^3 - D\,\epsilon_P^2\,\kappa_B + (\epsilon_B - \epsilon_P)\sqrt{\left(\epsilon_P\,\kappa_B\left(D\,\epsilon_P\,(\rho\,S_M + D\,(-c\,\rho + \kappa_B) + \rho\,\epsilon_P\,(-1 + \phi_S)) + \rho\,\epsilon_B\left((-1 + c)\,c\,D^2 + S_M^2 + S_M\,(D - 2\,c\,D + 2\,\epsilon_P\,(-1 + \phi_S)) - (-1 + 2\,c)\,D\,\epsilon_P\,(-1 + \phi_S) + \epsilon_P^2\,(-1 + \phi_S)^2\right)\right)\right)} + \epsilon_B\,\epsilon_P\,(-S_M\,(\rho - 2\,\kappa_B) + D\,(c\,\rho + \kappa_B - 2\,c\,\kappa_B) - \epsilon_P\,(\rho - 2\,\kappa_B)\,(-1 + \phi_S)) - \rho\,\epsilon_P^3\,\phi_S\Big);$$

$$\text{Simplify}\left[-\frac{\rho\,(c\,D - S_M + \epsilon_P\,\phi_P)}{D - \epsilon_B\,\phi_B}\ /.\ \phi_P \to 1 - \phi_B - \phi_S\ /.\ \phi_B \to \text{optimalBiosynthesis}\ /.\ \kappa_B \to \rho\ /.\ \epsilon_B \to \delta\,\epsilon_P,\ \text{assumptions}\right]$$

Out[∘]=

$$-\frac{\rho\left(D - c\,D\,\delta + \delta\,S_M - \delta\,\epsilon_P + \sqrt{(D - c\,D + S_M + \epsilon_P\,(-1 + \phi_S))\,(D - c\,D\,\delta + \delta\,S_M + \delta\,\epsilon_P\,(-1 + \phi_S))} + \delta\,\epsilon_P\,\phi_S\right)}{D - c\,D\,\delta + \delta\,S_M - \delta\,\epsilon_P + \delta\sqrt{(D - c\,D + S_M + \epsilon_P\,(-1 + \phi_S))\,(D - c\,D\,\delta + \delta\,S_M + \delta\,\epsilon_P\,(-1 + \phi_S))} + \delta\,\epsilon_P\,\phi_S}$$

In[∘]:=
```
Simplify[Normal[Series[%, {D, 0, 1}]], assumptions]
```

Out[∘]=

$$\frac{\rho\left(-D\left(1 + \sqrt{\delta}\right)^2 + 2\,\delta\,S_M + 2\,\delta\,\epsilon_P\,(-1 + \phi_S)\right)}{2\,\delta^{3/2}\,(S_M + \epsilon_P\,(-1 + \phi_S))}$$

Taylor Series Expansion:

In[∘]:=

$$\text{Series}[\%, \{D, 0, 1\}]\ /.\ \delta \to \frac{\epsilon_B}{\epsilon_P}\ //\ \text{Normal}$$

Out[∘]=

$$\frac{\rho}{\sqrt{\frac{\epsilon_B}{\epsilon_P}}} - \frac{D\,\rho\left(1 + \sqrt{\frac{\epsilon_B}{\epsilon_P}}\right)^2}{2\left(\frac{\epsilon_B}{\epsilon_P}\right)^{3/2}(S_M + \epsilon_P\,(-1 + \phi_S))}$$

# Figures

Exact model refers to the versions of the model that explicitly accounts for the substrate dynamics

(described in S4 Text).

## Fig 3: Trade-off

In[∘]:=
```
(*panel a: useparameters = {ϵP→2,ϵB→1, ϕS→0.1, c→0.8, D→0, SM→0};*)
useparameters = {ϵP → 2, ϵB → 1, ϕS → 0.1, c → 0.8, D → 0, SM → 0};
Show[
 Plot[{ϕB} /. useparameters, {ϕB, 0, 1},
  PlotRange → {{0, 1}, {0, 1}}, PlotStyle → {RGBColor[.4, .8, .8]}],
 Plot[{ϵB saturation} /. useparameters, {ϕB, 0, 1},
  PlotRange → {{0, 1}, {0, 1}}, PlotStyle → {RGBColor[.9, .8, .3]}],
 Plot[{birth} /. useparameters, {ϕB, 0, 1},
  PlotRange → {{0, 1}, {0, 1}}, PlotStyle → {RGBColor[.7, .1, .5]}],
 ListPlot[
  {{ϕB /. biosynthesisOptimal⟦1⟧, 0}, {ϕB /. biosynthesisOptimal⟦1⟧, 1}} /. δ → ϵB/ϵP /.
   useparameters, Joined → True, PlotStyle → {Black}]
]
```

Out[∘]=

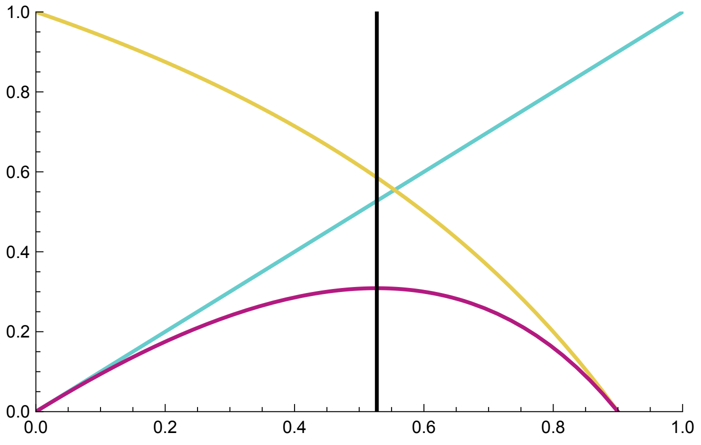

```
(*panels b-g:;
panel b: useparametersalt = {ϵP→2,ϵB→.2, ϕS→0.1, c→0.8, D→0, SM→0};
panel c: useparametersalt = {ϵP→.1,ϵB→1, ϕS→0.1, c→0.8, D→0, SM→0};
panel d: useparametersalt = {ϵP→2,ϵB→1, ϕS→0.4, c→0.8, D→0, SM→0};
panel e: useparametersalt = {ϵP→2,ϵB→1, ϕS→0.1, c→0.8, D→0.4, SM→0};
panel f: useparametersalt = {ϵP→2,ϵB→1, ϕS→0.1, c→0.8, D→0, SM→0.5};
*)

useparameters = {ϵP → 2, ϵB → 1, ϕS → 0.1, c → 0.8, D → 0, SM → 0};
useparametersalt = {ϵP → 2, ϵB → 1, ϕS → 0.1, c → 0.8, D → 0, SM → 0.5};
Show[
 Plot[{ϕB} /. useparameters, {ϕB, 0, 1},
  PlotRange → {{0, 1}, {0, 1}}, PlotStyle → RGBColor[.4, .8, .8],
  AxesStyle → Directive[Black, Thick], TicksStyle → 15],
 Plot[{ϵB saturation } /. useparameters, {ϕB, 0, 1},
  PlotRange → {{0, 1}, {0, 1}}, PlotStyle → RGBColor[.9, .8, .3]],
 Plot[{birth } /. useparameters, {ϕB, 0, 1},
  PlotRange → {{0, 1}, {0, 1}}, PlotStyle → RGBColor[.7, .1, .5]],
 ListPlot[
  {{ϕB /. biosynthesisOptimal⟦1⟧, 0}, {ϕB /. biosynthesisOptimal⟦1⟧, 1}} /. δ → ϵB/ϵP /.
   useparameters, Joined → True, PlotStyle → {Black}],
 Plot[{ϵB saturation } /. useparametersalt, {ϕB, 0, 1},
  PlotRange → {{0, 1}, {0, 1}}, PlotStyle → {RGBColor[.9, .8, .3], Dashing[0.01]}],
 Plot[{birth } /. useparametersalt, {ϕB, 0, 1}, PlotRange → {{0, 1}, {0, 1}},
  PlotStyle → {RGBColor[.7, .1, .5], Dashing[0.01]}],
 ListPlot[
  {{ϕB /. biosynthesisOptimal⟦1⟧, 0}, {ϕB /. biosynthesisOptimal⟦1⟧, 1}} /. δ → ϵB/ϵP /.
   useparametersalt, Joined → True, PlotStyle → {Black, Dashing[0.01]}]
]
```

Out[ ]=

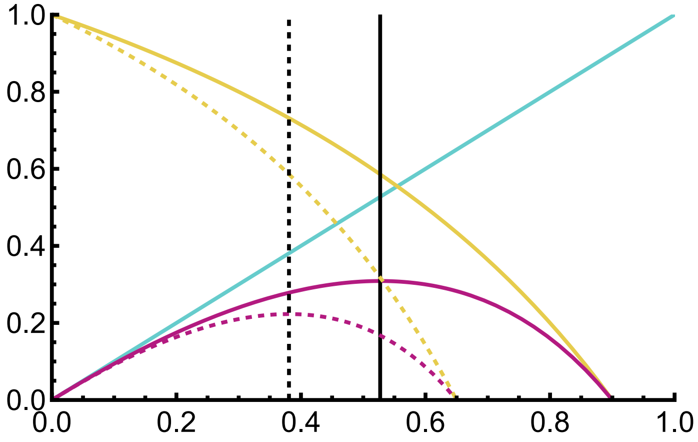

## Fig B: Trade-off_exact

In[ ]:=
```
useparameters = {ϵP → 2, ϵB → 1, ϕS → 0.1, c → 0.8, D → 0, SM → 0};
Show[

 Plot[{birth} /. useparameters, {ϕB, 0, 1},
  PlotRange → {{0, 1}, {0, 1}}, PlotStyle → ■, PlotLegends → {"Equation (2)"},
  AxesStyle → Directive[Black, Thick], TicksStyle → 13],
 Plot[{birthExact} /. useparameters /. {κB → 1, ρ → 10}, {ϕB, 0, 1},
  PlotRange → {{0, 1}, {0, 1}}, PlotStyle → ■, PlotLegends → {"κB/ρ=0.1"}],
 Plot[{birthExact} /. temp → Supply - ConsumptionMax + D κB /.
      {ConsumptionMax → ϕB ϵB ρ, Supply → (ϕP ϵP + c D - SM) ρ} /.
     ϕP → 1 - ϕS - ϕB /. useparameters /. {κB → 10, ρ → 1}, {ϕB, 0, 1},
  PlotRange → {{0, 1}, {0, 1}}, PlotStyle → ■, PlotLegends → {"κB/ρ=10"}],
 Plot[{birthExact} /. temp → Supply - ConsumptionMax + D κB /.
      {ConsumptionMax → ϕB ϵB ρ, Supply → (ϕP ϵP + c D - SM) ρ} /. ϕP → 1 - ϕS - ϕB /.
    useparameters /. {κB → 1, ρ → 1}, {ϕB, 0, 1}, PlotRange → {{0, 1}, {0, 1}},
  PlotStyle → {■, Dashing[0.02]}, PlotLegends → {"κB/ρ=1"}](*,
 ListPlot[
  {{ϕB/.BiosynthesisOptimalExact⟦1⟧,0},{ϕB/.BiosynthesisOptimalExact⟦1⟧,1}}/.
    useparameters/.{ κB→1,ρ→10}, Joined→True],
 ListPlot[
  {{ϕB/.BiosynthesisOptimalExact⟦2⟧,0},{ϕB/.BiosynthesisOptimalExact⟦2⟧,1}}/.
    useparameters/.{ κB→1,ρ→10}, Joined→True, PlotStyle→■]*)
]
```

Out[ ]=

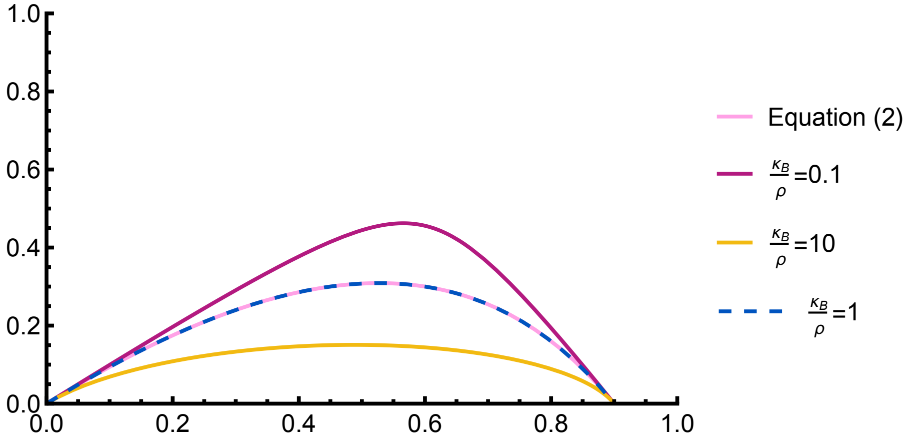

## Fig 4: Cost of traits

In[ ]:=
```
useparametersref = {ϵB → 1, ϕS → 0.5, c → 0.8, D → 0.01, SM → 0.005};
useparameterstraitMacroMol = {ϵB → 1, ϕS → 0.55, c → .8, D → 0.01, SM → 0.005};
useparameterstraitEnergy = {ϵB → 1, ϕS → 0.5, c → 0.8, D → 0.01, SM → 0.055};
useparameterstraitDeg = {ϵB → 1, ϕS → 0.5, c → 0.8, D → 0.06, SM → 0.005};
useparameterstraitslowbios = {ϵB → 0.95, ϕS → 0.5, c → 0.8, D → 0.01, SM → 0.005};
Show[
 Plot[{(birthOptimal /. δ → ϵB/ϵP /. useparametersref) -
     (birthOptimal /. δ → ϵB/ϵP /. useparameterstraitMacroMol)},
  {ϵP, 0, 10}, PlotStyle → ■, PlotRange → {{0., 10}, {0, .05}},
  AxesStyle → Directive[Black, Thick], AspectRatio → .8, TicksStyle → 14],
 Plot[{(birthOptimal /. δ → ϵB/ϵP /. useparametersref) -
     (birthOptimal /. δ → ϵB/ϵP /. useparameterstraitEnergy)},
  {ϵP, 0.12, 10}, PlotStyle → ■, PlotRange → All],
 Plot[{(birthOptimal /. δ → ϵB/ϵP /. useparametersref) -
     (birthOptimal /. δ → ϵB/ϵP /. useparameterstraitDeg)}, {ϵP, 0, 10}, PlotStyle → ■],
 Plot[{(birthOptimal /. δ → ϵB/ϵP /. useparametersref) -
     (birthOptimal /. δ → ϵB/ϵP /. useparameterstraitslowbios)}, {ϵP, 0, 10},
  PlotStyle → ■](*ListPlot[{{((-1+c) D-SM) ϵB/(D+ϵB (-1+ϕS)),0},{((-1+c) D-SM) ϵB/(D+ϵB (-1+ϕS)),1}}/.
   useparameterstraitEnergy, Joined→True]*)
]
```

Out[ ]=

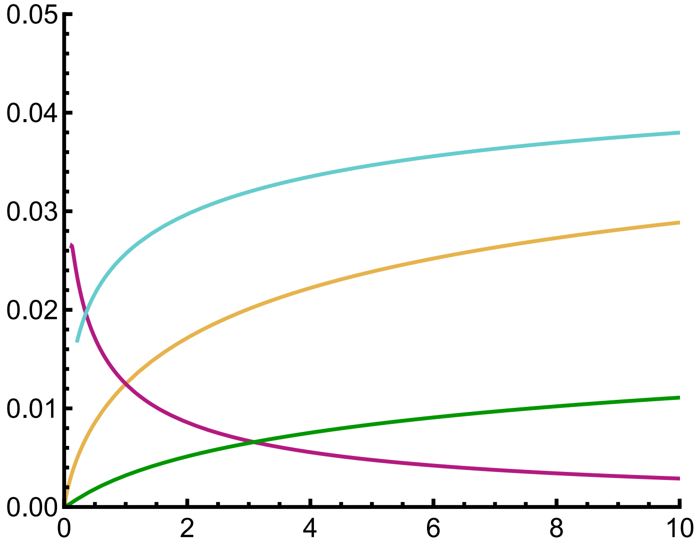

```
In[]:= useparametersref = {ϵP → 1.5, ϕS → 0.5, c → 0.8, D → 0.01, SM → 0.005};
useparameterstraitMacroMol = {ϵP → 1.5, ϕS → 0.55, c → .8, D → 0.01, SM → 0.005};
useparameterstraitEnergy = {ϵP → 1.5, ϕS → 0.5, c → .8, D → 0.01, SM → 0.055};
useparameterstraitDeg = {ϵP → 1.5, ϕS → 0.5, c → .8, D → 0.06, SM → 0.005};
useparameterstraitslowprov = {ϵP → 1.45, ϕS → 0.5, c → 0.8, D → 0.01, SM → 0.005};
Show[
 Plot[{(birthOptimal /. δ → ϵB/ϵP /. useparametersref) -
     (birthOptimal /. δ → ϵB/ϵP /. useparameterstraitMacroMol)},
  {ϵB, 0, 10}, PlotStyle → ■, PlotRange → {{0., 10}, {0, .05}},
  AxesStyle → Directive[Black, Thick], AspectRatio → .8,
  PlotLegends → {"Macromoleculre production (I)"}, TicksStyle → 14],
 Plot[{(birthOptimal /. δ → ϵB/ϵP /. useparametersref) -
     (birthOptimal /. δ → ϵB/ϵP /. useparameterstraitEnergy)},
  {ϵB, 0, 10}, PlotStyle → ■, PlotLegends → {"Energy consumption (II)"}],
 Plot[{(birthOptimal /. δ → ϵB/ϵP /. useparametersref) -
     (birthOptimal /. δ → ϵB/ϵP /. useparameterstraitDeg)}, {ϵB, 0.12, 10},
  PlotStyle → ■, PlotLegends → {"Increased degradation (III)"}],
 Plot[{(birthOptimal /. δ → ϵB/ϵP /. useparametersref) -
     (birthOptimal /. δ → ϵB/ϵP /. useparameterstraitslowprov)}, {ϵB, 0, 10},
  PlotStyle → ■, PlotLegends → {"Slower provisioning (IV)"}],
 Plot[{-0.01}, {ϵB, 0, 10}, PlotStyle → ■,
  PlotLegends → {"Slower biosynthesis (IV)"}]
 (*ListPlot[{{{D ϵP/(-D+c D-SM+ϵP-ϵP ϕS),0},{D ϵP/(-D+c D-SM+ϵP-ϵP ϕS),1}}/.useparameterstraitDeg,
  Joined→True, PlotStyle→Black]*)
]
```

Out[ ]=

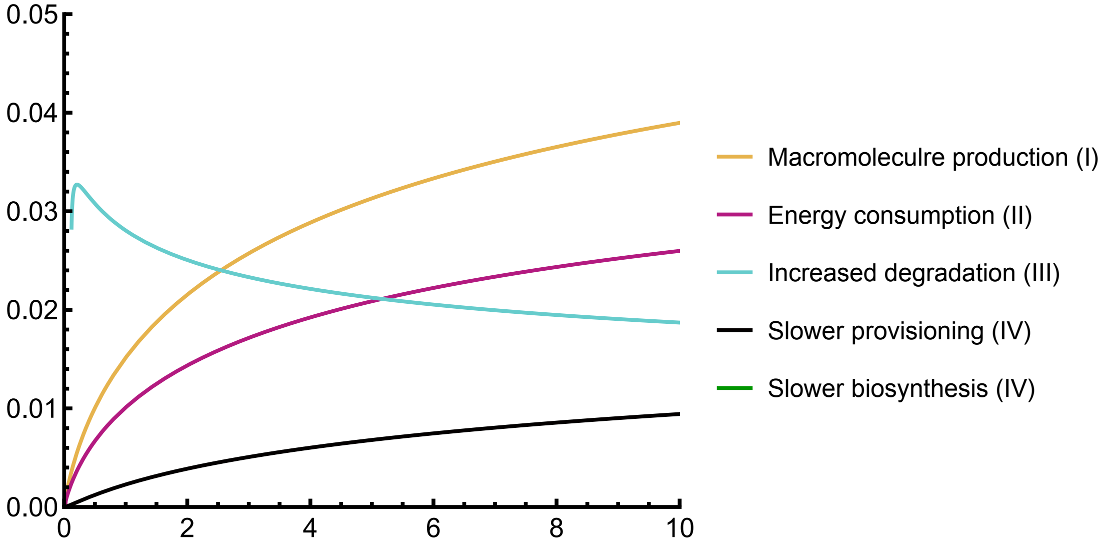
0.05
0.04
0.03
0.02
0.01
0.00
0
2
4
6
8
10
Macromoleculre production (I)
Energy consumption (II)
Increased degradation (III)
Slower provisioning (IV)
Slower biosynthesis (IV)

In[ ]:=

```
useparameterstraitMacroMolHighDeg =
  {ϵP → 1.5, ϵB → 1, ϕS → 0.5 + z, c → .8, D → 0.2, SM → 0.005};
useparameterstraitEnergyHighDeg =
  {ϵP → 1.5, ϵB → 1, ϕS → 0.5, c → .8, D → 0.2, SM → 0.005 + z};
useparameterstraitMacroMolLowDeg =
  {ϵP → 1.5, ϵB → 1, ϕS → 0.5 + z, c → .8, D → 0.01, SM → 0.005};
useparameterstraitEnergyLowDeg =
  {ϵP → 1.5, ϵB → 1, ϕS → 0.5, c → .8, D → 0.01, SM → 0.005 + z};
Show[
 Plot[{(birthOptimal /. δ → ϵB/ϵP /. useparameterstraitMacroMolLowDeg)}, {z, 0, 0.485},
  PlotStyle → Directive[■, Dashed], PlotRange → {{0, 0.75}, {0, 0.2}},
  AxesStyle → Directive[Black, Thick], AspectRatio → .8, TicksStyle → 14],
 Plot[{(birthOptimal /. δ → ϵB/ϵP /. useparameterstraitEnergyLowDeg)},
  {z, 0, 0.73}, PlotStyle → Directive[■, Dashed], PlotLegends → {"D= 0.01"}],
 Plot[{(birthOptimal /. δ → ϵB/ϵP /. useparameterstraitMacroMolHighDeg)},
  {z, 0, 0.26}, PlotStyle → ■, PlotRange → {{0, 0.73}, {0, 0.2}},
  AxesStyle → Directive[Black, Thick], AspectRatio → .8, TicksStyle → 14],
 Plot[{(birthOptimal /. δ → ϵB/ϵP /. useparameterstraitEnergyHighDeg)},
  {z, 0, 0.39}, PlotStyle → ■, PlotLegends → {" D = 0.2"}]

 (*ListPlot[{{(D ϵP)/(-D+c D-SM+ϵP-ϵP ϕS),0},{(D ϵP)/(-D+c D-SM+ϵP-ϵP ϕS),1}}/.useparameterstraitDeg,
  Joined→True, PlotStyle→Black]*)
]
```

Out[ ]=

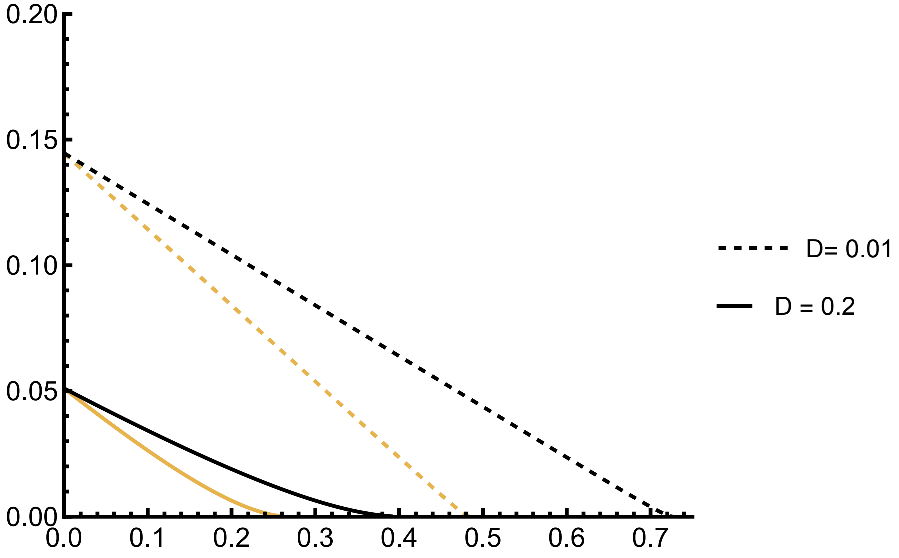

## Comparing cost of degradation using Taylor approximation and the full expression (not in the paper or S1 Appendix)

In[ ]:=
```
useparametersref = {ϵB → 2, ϕS → 0.1, c → 0.1, D → 0, SM → 0};
useparameterstraitMacroMol = {ϵB → 2, ϕS → 0.3, c → 0.1, D → 0, SM → 0};
useparameterstraitEnergy = {ϵB → 2, ϕS → 0.1, c → 0.1, D → 0., SM → 0.3};
useparameterstraitDeg = {ϵB → 2, ϕS → 0.1, c → 0.1, D → 0.3, SM → 0};
useparameterstraitslowbios = {ϵB → 1.5, ϕS → 0.1, c → 0.1, D → 0, SM → 0};
Show[
 Plot[{(birthOptimal /. δ → ϵB/ϵP /. useparametersref) -
    (birthOptimal /. δ → ϵB/ϵP /. useparameterstraitMacroMol)}, {ϵP, 0, 10},
  PlotStyle → RGBColor[.9, .8, .3], PlotRange → {{0., 10}, {0, .3}},
  AxesStyle → Directive[Black, Thick], AspectRatio → .8, TicksStyle → 14],
 Plot[{(birthOptimal /. δ → ϵB/ϵP /. useparametersref) -
    (birthOptimal /. δ → ϵB/ϵP /. useparameterstraitEnergy)},
  {ϵP, 0, 10}, PlotStyle → RGBColor[.7, .1, .5]],
 Plot[{(birthOptimal /. δ → ϵB/ϵP /. useparametersref) -
    (birthOptimal /. δ → ϵB/ϵP /. useparameterstraitDeg)},
  {ϵP, 0, 10}, PlotStyle → RGBColor[.4, .8, .8]],
 Plot[{(birthOptimalTaylor1 /. δ → ϵB/ϵP /. useparametersref) -
    (birthOptimalTaylor1 /. δ → ϵB/ϵP /. useparameterstraitDeg)},
  {ϵP, 0, 10}, PlotStyle → {RGBColor[.4, .8, .8], Dashing[0.01]}],
 Plot[{(birthOptimal /. δ → ϵB/ϵP /. useparametersref) -
    (birthOptimal /. δ → ϵB/ϵP /. useparameterstraitslowbios)},
  {ϵP, 0, 10}, PlotStyle → {Green}],
 ListPlot[{{((-1 + c) D - SM) ϵB/(D + ϵB (-1 + ϕS)), 0}, {((-1 + c) D - SM) ϵB/(D + ϵB (-1 + ϕS)), 1}} /.
   useparameterstraitEnergy, Joined → True]
]
```

Out[ ]=

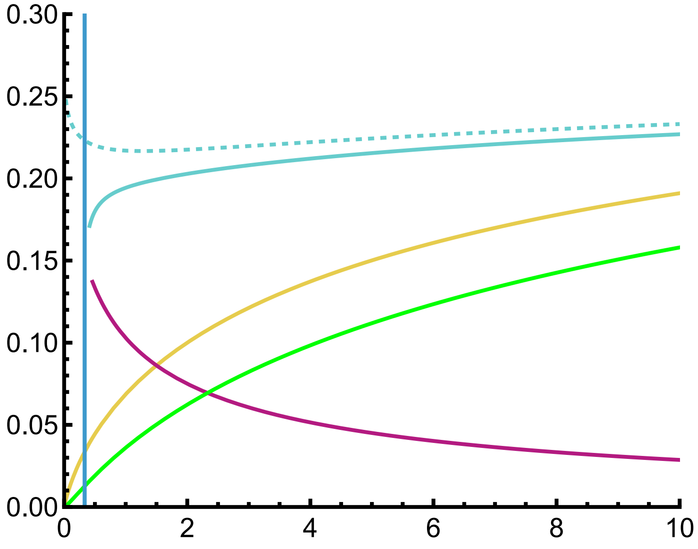

```
In[•]:= useparametersref = {ϵP → 2, ϕS → 0.1, c → 0, D → 0, SM → 0};
useparameterstraitMacroMol = {ϵP → 2, ϕS → 0.3, c → .0, D → 0, SM → 0};
useparameterstraitEnergy = {ϵP → 2, ϕS → 0.1, c → .0, D → 0., SM → 0.3};
useparameterstraitDeg = {ϵP → 2, ϕS → 0.1, c → .0, D → 0.3, SM → 0};
Show[
 Plot[{(birthOptimal /. δ → ϵB/ϵP /. useparametersref) -
    (birthOptimal /. δ → ϵB/ϵP /. useparameterstraitMacroMol)}, {ϵB, 0, 10},
  PlotStyle → RGBColor[.9, .8, .3], PlotRange → {{0., 10}, {0, .3}},
  AxesStyle → Directive[Black, Thick], AspectRatio → .8,
  PlotLegends → {"Macromoleculre production"}, TicksStyle → 14],
 Plot[{(birthOptimal /. δ → ϵB/ϵP /. useparametersref) -
    (birthOptimal /. δ → ϵB/ϵP /. useparameterstraitEnergy)}, {ϵB, 0, 10},
  PlotStyle → RGBColor[.7, .1, .5], PlotLegends → {"Energy consumption"}],
 Plot[{(birthOptimal /. δ → ϵB/ϵP /. useparametersref) -
    (birthOptimal /. δ → ϵB/ϵP /. useparameterstraitDeg)}, {ϵB, 0, 10},
  PlotStyle → RGBColor[.4, .8, .8], PlotLegends → {"Increased degradation"}],
 Plot[{(birthOptimalTaylor1 /. δ → ϵB/ϵP /. useparametersref) -
    (birthOptimalTaylor1 /. δ → ϵB/ϵP /. useparameterstraitDeg)},
  {ϵB, 0, 10}, PlotStyle → {RGBColor[.4, .8, .8], Dashing[0.01]}],
 ListPlot[{{(D ϵP)/(-D + c D - SM + ϵP - ϵP ϕS), 0}, {(D ϵP)/(-D + c D - SM + ϵP - ϵP ϕS), 1}} /.
   useparameterstraitDeg, Joined → True, PlotStyle → Black]
]
```

Out[ ]=

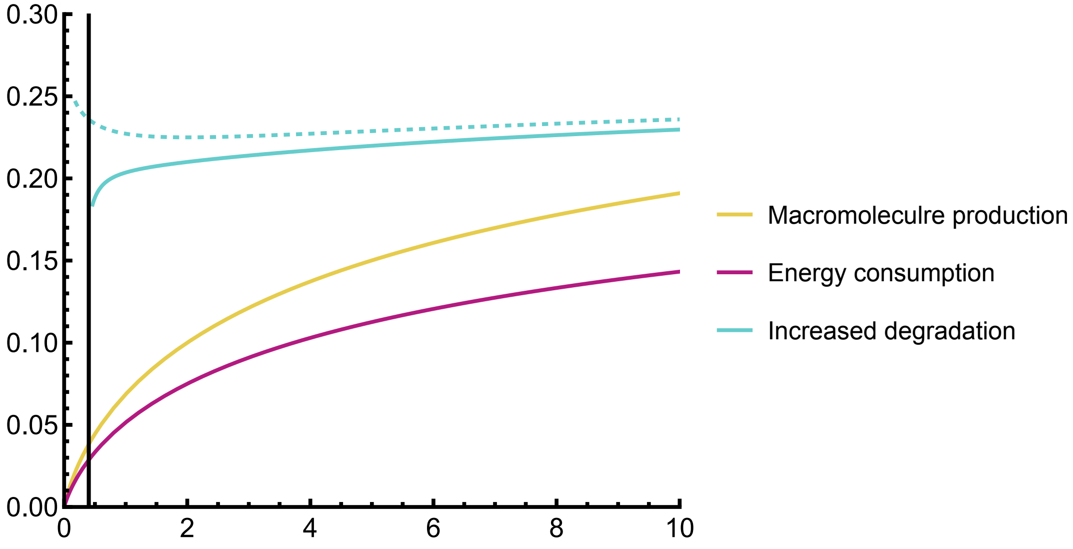
0.30
0.25
0.20
0.15
0.10
0.05
0.00
0
2
4
6
8
10
Macromoleculre production
Energy consumption
Increased degradation

## Fig C: Cost of traits_exact

```
In[ ]:= useparametersref = {ϵB → 2, ϕS → 0.5, c → 0.8, D → 0, SM → 0 , κB → 1, ρ → 10};
useparameterstraitMacroMol = {ϵB → 2, ϕS → 0.55, c → 0.8, D → 0, SM → 0, κB → 1, ρ → 10};
useparameterstraitEnergy =
  {ϵB → 2, ϕS → 0.5, c → 0.8, D → 0., SM → 0.05, κB → 1, ρ → 10};
useparameterstraitDeg = {ϵB → 2, ϕS → 0.5, c → 0.8, D → 0.05, SM → 0, κB → 1, ρ → 10};
useparameterstraitDegnoRecycle =
  {ϵB → 2, ϕS → 0.5, c → 0, D → 0.05, SM → 0, κB → 1, ρ → 10};
useparameterstraitslowbios =
  {ϵB → 1.95, ϕS → 0.5, c → 0.8, D → 0, SM → 0, κB → 1, ρ → 10};
Show[
 Plot[{(birthExact /. ϕB → BiosynthesisOptimalExactRelevant[
          ϵP, ϵB, ϕS, c, D, SM, κB, ρ] /. useparametersref) -
     (birthExact /. ϕB → BiosynthesisOptimalExactRelevant[ϵP, ϵB, ϕS,
          c, D, SM, κB, ρ] /. useparameterstraitMacroMol)},
  {ϵP, 0, 10}, PlotStyle → ■,  PlotRange → {{0., 10}, {0, .07}},
  AxesStyle → Directive[Black, Thick],
  AspectRatio → .8,
  TicksStyle → 14],
 Plot[{(birthExact /. ϕB → BiosynthesisOptimalExactRelevant[
          ϵP, ϵB, ϕS, c, D, SM, κB, ρ] /. useparametersref) -
     (birthExact /. ϕB → BiosynthesisOptimalExactRelevant[ϵP, ϵB, ϕS,
          c, D, SM, κB, ρ] /. useparameterstraitEnergy)},
  {ϵP, 0, 10}, PlotStyle → ■, PlotRange → All],
 Plot[{(birthExact /. ϕB → BiosynthesisOptimalExactRelevant[
          ϵP, ϵB, ϕS, c, D, SM, κB, ρ] /. useparametersref) -
     (birthExact /. ϕB → BiosynthesisOptimalExactRelevant[ϵP, ϵB, ϕS, c,
          D, SM, κB, ρ] /. useparameterstraitDeg)}, {ϵP, 0, 10}, PlotStyle → ■],
 Plot[{(birthExact /. ϕB → BiosynthesisOptimalExactRelevant[ϵP, ϵB,
          ϕS, c, D, SM, κB, ρ] /. useparametersref) - (birthExact /.
       ϕB → BiosynthesisOptimalExactRelevant[ϵP, ϵB, ϕS, c, D, SM, κB, ρ] /.
      useparameterstraitslowbios)}, {ϵP, 0, 10}, PlotStyle → ■],
 Plot[{(birthExact /. ϕB → BiosynthesisOptimalExactRelevant[ϵP,
          ϵB, ϕS, c, D, SM, κB, ρ] /. useparametersref) - (birthExact /.
       ϕB → BiosynthesisOptimalExactRelevant[ϵP, ϵB, ϕS, c, D, SM, κB, ρ] /.
      useparameterstraitDegnoRecycle)}, {ϵP, 0, 10}, PlotStyle → ■]
 (*ListPlot[
  {{((-1+c) D-SM) ϵB/(D+ϵB (-1+ϕS)),0},{((-1+c) D-SM) ϵB/(D+ϵB (-1+ϕS)),1}}/.useparameterstraitEnergy, Joined→True]*)
]
```

Out[ ]=

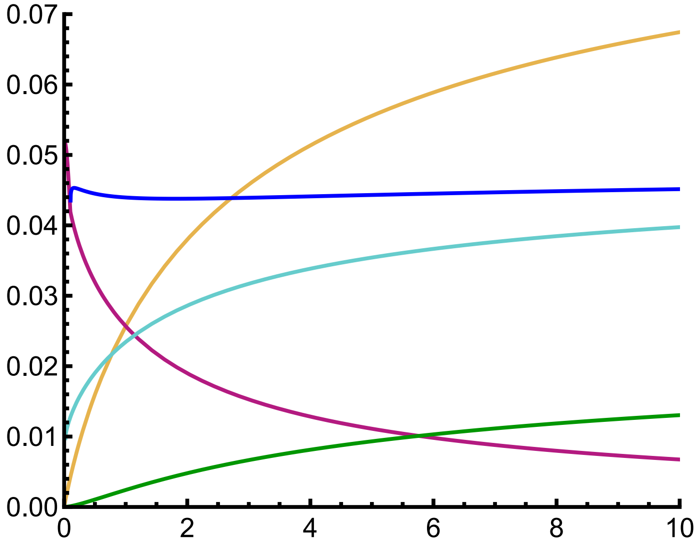


In[ ]:=
```
useparametersref = {ϵP → 2, ϕS → 0.5, c → 0.8, D → 0, SM → 0 , κB → 1, ρ → 10};
useparameterstraitMacroMol = {ϵP → 2, ϕS → 0.55, c → 0.8, D → 0, SM → 0, κB → 1, ρ → 10};
useparameterstraitEnergy =
  {ϵP → 2, ϕS → 0.5, c → 0.8, D → 0., SM → 0.05, κB → 1, ρ → 10};
useparameterstraitDeg = {ϵP → 2, ϕS → 0.5, c → 0.8, D → 0.05, SM → 0, κB → 1, ρ → 10};
useparameterstraitDegnoRecycle =
  {ϵP → 2, ϕS → 0.5, c → 0, D → 0.05, SM → 0, κB → 1, ρ → 10};
useparameterstraitslowprov =
  {ϵP → 1.95, ϕS → 0.5, c → 0.8, D → 0, SM → 0, κB → 1, ρ → 10};
Show[
 Plot[{(birthExact /. ϕB → BiosynthesisOptimalExactRelevant [
          ϵP, ϵB, ϕS, c, D, SM, κB, ρ] /. useparametersref) -
     (birthExact /. ϕB → BiosynthesisOptimalExactRelevant [ϵP, ϵB, ϕS,
          c, D, SM, κB, ρ] /. useparameterstraitMacroMol)},
  {ϵB, 0, 10}, PlotStyle → ■, PlotRange → {{0., 10}, {0, .07}},
  AxesStyle → Directive[Black, Thick],
  AspectRatio → .8,
  PlotLegends → {"Macromolecule production"},
  TicksStyle → 14],
 Plot[{(birthExact /. ϕB → BiosynthesisOptimalExactRelevant [
          ϵP, ϵB, ϕS, c, D, SM, κB, ρ] /. useparametersref) -
     (birthExact /. ϕB → BiosynthesisOptimalExactRelevant [ϵP, ϵB, ϕS,
          c, D, SM, κB, ρ] /. useparameterstraitEnergy)},
  {ϵB, 0, 10}, PlotStyle → ■, PlotRange → All, PlotLegends →
   {"Energy consumption"}],
 Plot[{(birthExact /. ϕB → BiosynthesisOptimalExactRelevant [
          ϵP, ϵB, ϕS, c, D, SM, κB, ρ] /. useparametersref) -
     (birthExact /. ϕB → BiosynthesisOptimalExactRelevant [ϵP, ϵB, ϕS,
```

```
        c, D, S_M, κ_B, ρ] /. useparameterstraitDeg)}, {ϵ_B, 0.06, 10},
  PlotStyle → ■, PlotLegends → {"Increased degradation(c=0.8)"}],
 Plot[{(birthExact /. ϕ_B → BiosynthesisOptimalExactRelevant[
        ϵ_P, ϵ_B, ϕ_S, c, D, S_M, κ_B, ρ] /. useparametersref) -
    (birthExact /. ϕ_B → BiosynthesisOptimalExactRelevant[ϵ_P, ϵ_B, ϕ_S,
        c, D, S_M, κ_B, ρ] /. useparameterstraitslowprov)},
  {ϵ_B, 0, 10}, PlotStyle → ■, PlotLegends → {"Slower provisioning"}],
 Plot[{(birthExact /. ϕ_B → BiosynthesisOptimalExactRelevant[
        ϵ_P, ϵ_B, ϕ_S, c, D, S_M, κ_B, ρ] /. useparametersref) -
    (birthExact /. ϕ_B → BiosynthesisOptimalExactRelevant[ϵ_P, ϵ_B, ϕ_S, c,
        D, S_M, κ_B, ρ] /. useparameterstraitDegnoRecycle)}, {ϵ_B, 0, 10},
  PlotStyle → ■, PlotLegends → {"Increased degradation(c=0)"}],
 Plot[{-0.01}, {ϵ_B, 0, 10}, PlotStyle → ■, PlotLegends → {"Slower biosynthesis"}]
 (*ListPlot[{{D ϵ_P/(-D+c D-S_M+ϵ_P-ϵ_P ϕ_S),0},{D ϵ_P/(-D+c D-S_M+ϵ_P-ϵ_P ϕ_S),1}}/.useparameterstraitDeg,
  Joined→True, PlotStyle→Black]*)
]
```

Out[ ]=

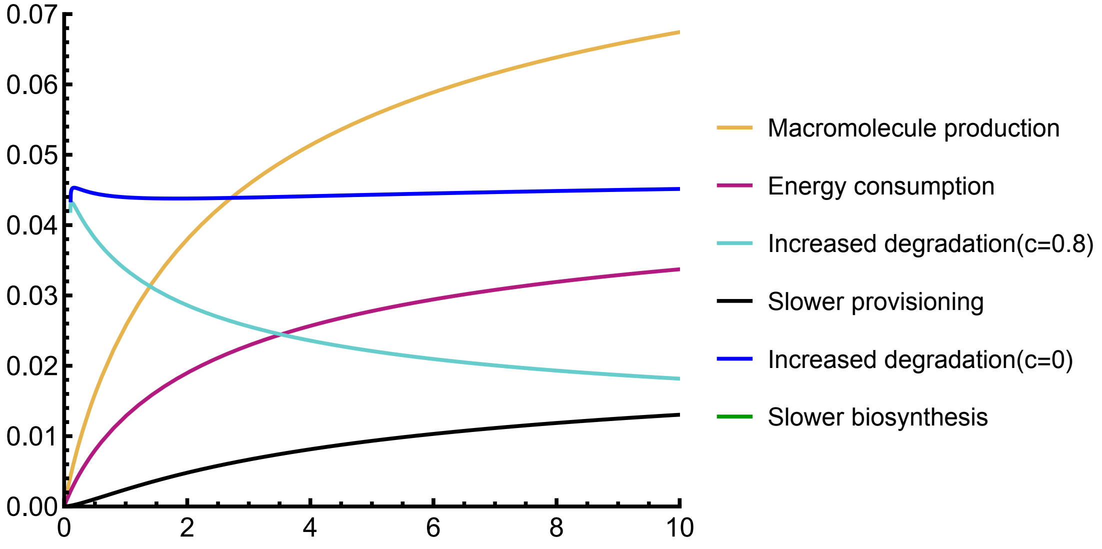

## Fig 5: Growth laws

```
In[∘]:= useparameters = {ϕS → 0.5, c → 0.8, ϵB → 2};
Show[
  ListPlot[Table[{birthOptimal /. δ → ϵB/ϵP, ϕB /. biosynthesisOptimal[[1]] /. δ → ϵB/ϵP} /.
       useparameters /. SM → 0 /. D → 0, {ϵP, 0.000, 5, 0.001}],
   Joined → True, PlotStyle → ■, AxesStyle → Directive[Black, Thick],
   AspectRatio → .8, PlotRange → All, TicksStyle → 14],
  ListPlot[Table[
     {birthOptimal /. δ → ϵB/ϵP, ϕB /. biosynthesisOptimal[[1]] /. δ → ϵB/ϵP} /. useparameters /.
      SM → 0.005 /. D → 0, {ϵP, 0.01, 10, 0.001}],
   Joined → True, PlotStyle → ■, PlotRange → All],
  ListPlot[Table[
     {birthOptimal /. δ → ϵB/ϵP, ϕB /. biosynthesisOptimal[[1]] /. δ → ϵB/ϵP} /. useparameters /.
      SM → 0 /. D → .01, {ϵP, 0.00, 10, 0.001}],
   Joined → True, PlotStyle → ■, PlotRange → All],
  ListPlot[Table[
     {birthOptimal /. δ → ϵB/ϵP, ϕB /. biosynthesisOptimal[[1]] /. δ → ϵB/ϵP} /. useparameters /.
      SM → 0.005 /. D → 0.01, {ϵP, 0.0, 15, 0.001}],
   Joined → True, PlotStyle → ■, PlotRange → All],
  (*ListPlot[Table[
     {birthOptimalTaylor1/.δ →ϵB/ϵP,biosynthesisOptimalTaylor1/.δ →ϵB/ϵP}/.useparameters/.
      SM→0.005/.D→0.01,{ϵP,0.015,10,0.001}],
   Joined→True,PlotStyle→{■,Dashing[0.01]},PlotRange→All],*)
  PlotRange → {{0, 0.1}, {0, 0.17}}
 ](*The Taylor approximation is very close to the exact
  result and including it does not help as it is indistinguishable*)
```

••• Power: Infinite expression $\frac{1}{0.}$ encountered.

••• Infinity: Indeterminate expression 0. + ComplexInfinity + ComplexInfinity encountered.

••• Power: Infinite expression $\frac{1}{0.}$ encountered.

Power: Infinite expression $\frac{1}{0.}$ encountered.

General: Further output of Power::infy will be suppressed during this calculation.

Infinity: Indeterminate expression 0. + ComplexInfinity + ComplexInfinity encountered.

Infinity: Indeterminate expression 0. + ComplexInfinity + ComplexInfinity encountered.

General: Further output of Infinity::indet will be suppressed during this calculation.

Out[ ]=

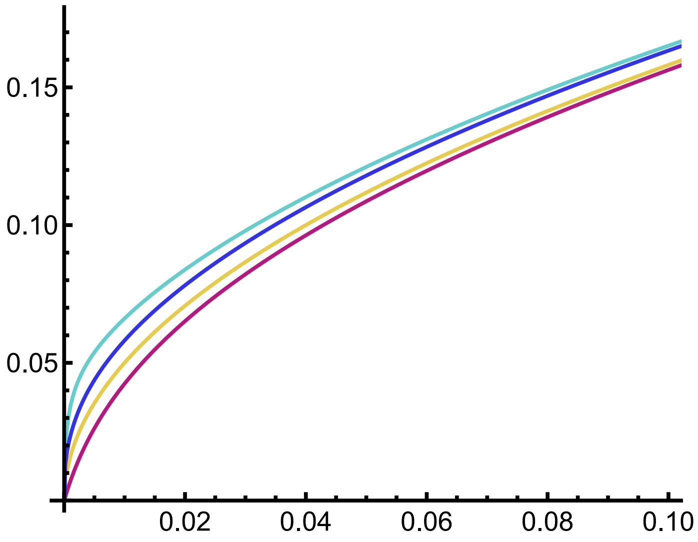


Zooming on the near zero growth regime of the previous graph

```
In[•]:= useparameters = {ϕS → 0.5, c → 0.8, ϵB → 2};
Show[
  ListPlot[Table[{birthOptimal /. δ → ϵB/ϵP, ϕB /. biosynthesisOptimal[[1]] /. δ → ϵB/ϵP} /.
      useparameters /. SM → 0 /. D → 0, {ϵP, 0.00001, 0.01, 0.00001}],
   Joined → True, PlotStyle → ■, AxesStyle → Directive[Black, Thick],
   AspectRatio → .8, PlotRange → All, TicksStyle → 16],
  ListPlot[Table[
     {birthOptimal /. δ → ϵB/ϵP, ϕB /. biosynthesisOptimal[[1]] /. δ → ϵB/ϵP} /. useparameters /.
       SM → 0.005 /. D → 0, {ϵP, 0.005, 0.02, 0.00001}],
   Joined → True, PlotStyle → ■, PlotRange → All],
  ListPlot[Table[
     {birthOptimal /. δ → ϵB/ϵP, ϕB /. biosynthesisOptimal[[1]] /. δ → ϵB/ϵP} /. useparameters /.
       SM → 0 /. D → .01, {ϵP, 0.00408, 0.02, 0.00001}],
   Joined → True, PlotStyle → ■, PlotRange → All],
  ListPlot[Table[
     {birthOptimal /. δ → ϵB/ϵP, ϕB /. biosynthesisOptimal[[1]] /. δ → ϵB/ϵP} /. useparameters /.
       SM → 0.005 /. D → 0.01, {ϵP, 0.0142, 0.02, 0.00001}],
   Joined → True, PlotStyle → ■, PlotRange → All],
  (*ListPlot[Table[
     {birthOptimalTaylor1/.δ →ϵB/ϵP,biosynthesisOptimalTaylor1/.δ →ϵB/ϵP}/.useparameters/.
       SM→0.005/.D→0.01,{ϵP,0.015,10,0.001}],
   Joined→True,PlotStyle→{■,Dashing[0.01]},PlotRange→All],*)
  PlotRange → {{0, 0.001}, {0, 0.04}}
 ](*The Taylor approximation is very close to the exact
  result and including it does not help as it is indistinguishable*)
```

Out[ ]=

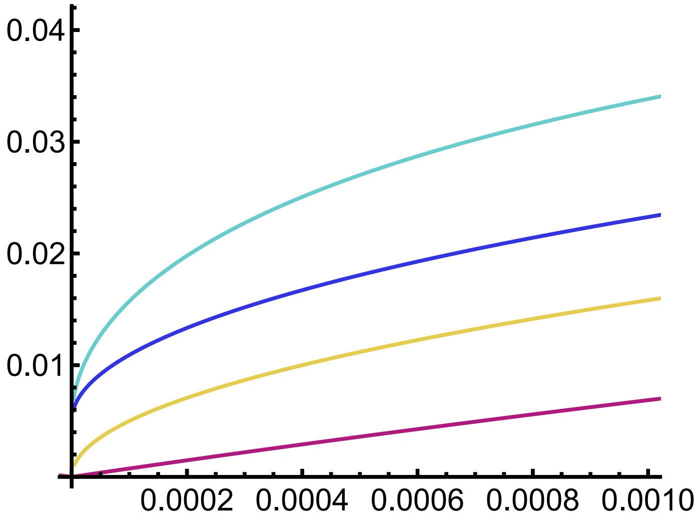


In[ ]:= (*Calculating the $\epsilon_P$ which results in birth rate being equal to zero*)

$$\frac{((-1 + c)\, D - S_M)\, \epsilon_B}{D + \epsilon_B\, (-1 + \phi_S)} \;/.\; \{S_M \to 0.005,\ D \to 0.01,\ \phi_S \to 0.5,\ c \to .8,\ \epsilon_B \to 2\}$$

Out[ ]=

0.0141414

```
In[•]:= useparameters = {ϕS → 0.5, c → 0.8, ϵB → 2};
Show[ListPlot[
   Table[{birthOptimal /. δ → ϵB/ϵP, saturationOptimal[[1]] /. δ → ϵB/ϵP} /. useparameters /.
      SM → 0 /. D → 0, {ϵP, 0.0000, 5, 0.001}], Joined → True,
   PlotStyle → ■, AxesStyle → Directive[Black, Thick], AspectRatio → .8,
   PlotLegends → {"D=0, SM=0"}, PlotRange → All, TicksStyle → 14],
  ListPlot[
   Table[{birthOptimal /. δ → ϵB/ϵP, saturationOptimal[[1]] /. δ → ϵB/ϵP} /. useparameters /.
      SM → 0.005 /. D → 0, {ϵP, 0.0, 5, 0.001}], Joined → True,
   PlotStyle → ■, PlotLegends → {"D=0, SM=0.005"}, PlotRange → All],
  ListPlot[
   Table[{birthOptimal /. δ → ϵB/ϵP, saturationOptimal[[1]] /. δ → ϵB/ϵP} /. useparameters /.
      SM → 0 /. D → 0.01, {ϵP, 0.000, 20, 0.001}], Joined → True,
   PlotStyle → ■, PlotLegends → {"D=0.01, SM=0"}, PlotRange → All],
  ListPlot[
   Table[{birthOptimal /. δ → ϵB/ϵP, saturationOptimal[[1]] /. δ → ϵB/ϵP} /. useparameters /.
      SM → 0.005 /. D → 0.01, {ϵP, 0, 20, 0.001}], Joined → True,
   PlotStyle → ■, PlotLegends → {"D=0.01, SM=0.005"}, PlotRange → All],
  (*ListPlot[Table[
    {birthOptimalTaylor1/.δ →ϵB/ϵP,saturationOptimalTaylor1/.δ →ϵB/ϵP}/.useparameters/.
      SM→0.005/.D→0.01,{ϵP,.015,10,0.001}],
   Joined→True,PlotStyle→{■,Dashing[0.01]},PlotLegends→
    {"D=0.01, SM=0.005; first-order Taylor approx"}, PlotRange→All],
  *) (*Plot[1/(1+Sqrt[-ϵB (-1+ϕS)/SM])/.SM→ 0.5/.useparameters,{ϵP,-0.01,0.01},PlotStyle→Black],*)
  PlotRange → {{0, .1}, {0, 0.5}}
 ]

••• Power: Infinite expression 1/0. encountered. ⓘ

••• Infinity: Indeterminate expression 0. + ComplexInfinity + ComplexInfinity encountered. ⓘ

••• Power: Infinite expression 1/0. encountered. ⓘ

••• Infinity: Indeterminate expression 0. + D + ComplexInfinity + ComplexInfinity encountered. ⓘ
```

Power: Infinite expression $\frac{1}{0.^2}$ encountered.

General: Further output of Power::infy will be suppressed during this calculation.

Infinity: Indeterminate expression 0. + ComplexInfinity + ComplexInfinity encountered.

General: Further output of Infinity::indet will be suppressed during this calculation.

Out[ ]=

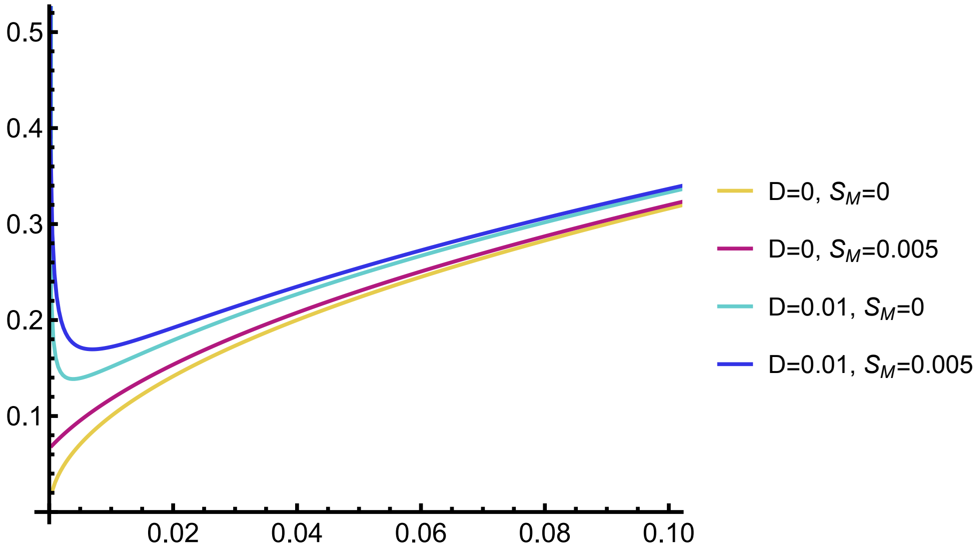


## Fig D: Growth laws_exact

In[ ]:=

```
(*Calculating the ϵP which results in birth rate being equal to zero*)
```

$$\frac{((-1+c)\,D - S_M)\,\epsilon_B}{D + \epsilon_B\,(-1+\phi_S)} \;/.\; \{S_M \to 0.5,\; D \to 0.5,\; \phi_S \to 0,\; c \to .9,\; \epsilon_B \to 2\}$$

Out[ ]=

```
0.733333
```

```
useparameters = {ϕS → 0.5, c → 0.8, ϵB → 2, ϰB → 1, ρ → 10};
Show[ListPlot[Table[
   {birthExact /. ϕB → BiosynthesisOptimalExactRelevant [ϵP, ϵB, ϕS, c, D, SM, ϰB, ρ],
      BiosynthesisOptimalExactRelevant [ϵP, ϵB, ϕS, c, D, SM, ϰB, ρ]} /.
     useparameters /. {D → 0.0, SM → 0.0}, {ϵP, 0., 5, 0.001}], Joined → True,
  PlotStyle → ■, AxesStyle → Directive[Black, Thick], AspectRatio → .8,
  PlotRange → {{0, .1}, {0, 0.17}}, TicksStyle → 14],
 ListPlot[Table[
   {birthExact /. ϕB → BiosynthesisOptimalExactRelevant [ϵP, ϵB, ϕS, c, D, SM, ϰB, ρ],
       BiosynthesisOptimalExactRelevant [ϵP, ϵB, ϕS, c, D, SM, ϰB, ρ]} /.
      useparameters /. SM → 0.005 /. D → 0, {ϵP, 0, 10, 0.001}],
  Joined → True, PlotStyle → ■, PlotRange → All],
 ListPlot[Table[
   {birthExact /. ϕB → BiosynthesisOptimalExactRelevant [ϵP, ϵB, ϕS, c, D, SM, ϰB, ρ],
       BiosynthesisOptimalExactRelevant [ϵP, ϵB, ϕS, c, D, SM, ϰB, ρ]} /.
      useparameters /. SM → 0 /. D → .01, {ϵP, 0.0, 10, 0.001}],
  Joined → True, PlotStyle → ■, PlotRange → All],
 ListPlot[Table[
   {birthExact /. ϕB → BiosynthesisOptimalExactRelevant [ϵP, ϵB, ϕS, c, D, SM, ϰB, ρ],
       BiosynthesisOptimalExactRelevant [ϵP, ϵB, ϕS, c, D, SM, ϰB, ρ]} /.
      useparameters /. SM → 0.005 /. D → 0.01, {ϵP, .0, 30, 0.001}],
  Joined → True, PlotStyle → ■, PlotRange → All]
 (*ListPlot[Table[{birthExact /.BiosynthesisOptimalExactrelevant[ϵP,ϵB/.ϵB→2],
       ϕB/.BiosynthesisOptimalExactrelevant[ϵP,ϵB/.ϵB→2]}/.useparameters/.
     SM→0.5/.D→0.5,{ϵP,.8,30,0.01}],Joined→True,
  PlotStyle→{RGBColor[.2,.2,.1],Dashing[0.01]}],*)

]
```

Power: Infinite expression $\frac{1}{0.}$ encountered.

Infinity: Indeterminate expression 0. ComplexInfinity encountered.

Power: Infinite expression $\frac{1}{0.}$ encountered.

Infinity: Indeterminate expression 0. ComplexInfinity encountered.

Power: Infinite expression $\frac{1}{0.}$ encountered.

General: Further output of Power::infy will be suppressed during this calculation.

Infinity: Indeterminate expression 0. ComplexInfinity encountered.

General: Further output of Infinity::indet will be suppressed during this calculation.

Out[•]=

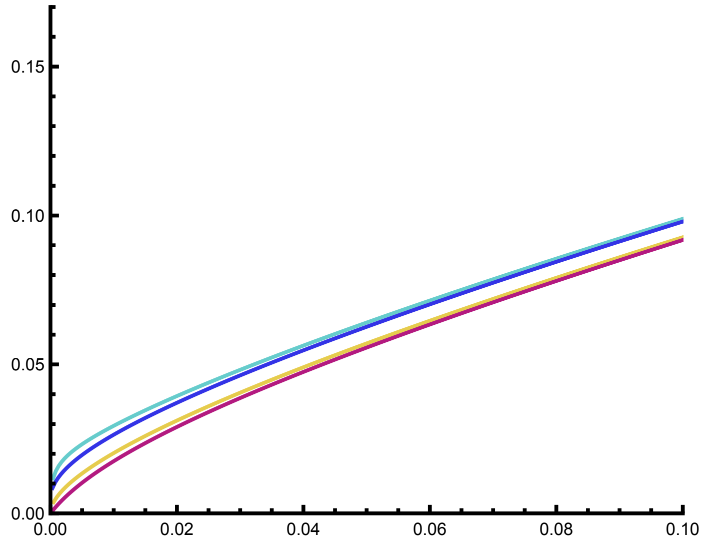


In[•]:=
```
useparameters = {ϕS → 0.5, c → .8, ϵB → 2, ϰB → 1, ρ → 10};
Show[ListPlot[Table[
    {birthExact /. ϕB → BiosynthesisOptimalExactRelevant [ϵP, ϵB, ϕS, c, D, SM, ϰB, ρ],
       SaturationOptimalExact [ϵP, ϵB, ϕS, c, D, SM, ϰB, ρ]} /. useparameters /.
     {D → 0.0, SM → 0.0}, {ϵP, 0., 5, 0.001}], Joined → True, PlotStyle → ■,
  AxesStyle → Directive[Black, Thick], AspectRatio → .8,
  PlotRange → {{0, .2}, {0, 1}}, PlotLegends → {"D=0, SM=0"}],
 ListPlot[Table[
    {birthExact /. ϕB → BiosynthesisOptimalExactRelevant [ϵP, ϵB, ϕS, c, D, SM, ϰB, ρ],
        SaturationOptimalExact [ϵP, ϵB, ϕS, c, D, SM, ϰB, ρ]} /. useparameters /.
      SM → 0.005 /. D → 0, {ϵP, 0, 10, 0.001}], Joined → True,
  PlotStyle → ■, PlotRange → All, PlotLegends → {"D=0, SM=0.005"}],
 ListPlot[Table[
    {birthExact /. ϕB → BiosynthesisOptimalExactRelevant [ϵP, ϵB, ϕS, c, D, SM, ϰB, ρ],
        SaturationOptimalExact [ϵP, ϵB, ϕS, c, D, SM, ϰB, ρ]} /. useparameters /.
      SM → 0 /. D → .01, {ϵP, 0.0, 10, 0.001}], Joined → True,
  PlotStyle → ■, PlotRange → All, PlotLegends → {"D=0.01, SM=0"}],
 ListPlot[Table[
    {birthExact /. ϕB → BiosynthesisOptimalExactRelevant [ϵP, ϵB, ϕS, c, D, SM, ϰB, ρ],
        SaturationOptimalExact [ϵP, ϵB, ϕS, c, D, SM, ϰB, ρ]} /. useparameters /.
      SM → 0.005 /. D → 0.01, {ϵP, .0, 30, 0.001}], Joined → True,
  PlotStyle → ■, PlotRange → All, PlotLegends → {"D=0.01, SM=0.005"}]
 (*ListPlot[Table[{birthExact /.BiosynthesisOptimalExactrelevant[ϵP,ϵB/.ϵB→2],
        ϕB/.BiosynthesisOptimalExactrelevant[ϵP,ϵB/.ϵB→2]}/.useparameters/.
      SM→0.5/.D→0.5,{ϵP,.8,30,0.01}],Joined→True,
  PlotStyle→{RGBColor[.2,.2,.1],Dashing[0.01]}],*)


]
```

Power: Infinite expression $\frac{1}{0.}$ encountered.

Infinity: Indeterminate expression 0. ComplexInfinity encountered.

Power: Infinite expression $\frac{1}{0.}$ encountered.

Infinity: Indeterminate expression 0. ComplexInfinity encountered.

Power: Infinite expression $\frac{1}{0.}$ encountered.

General: Further output of Power::infy will be suppressed during this calculation.

Infinity: Indeterminate expression 0. ComplexInfinity encountered.

General: Further output of Infinity::indet will be suppressed during this calculation.

Out[ ]=

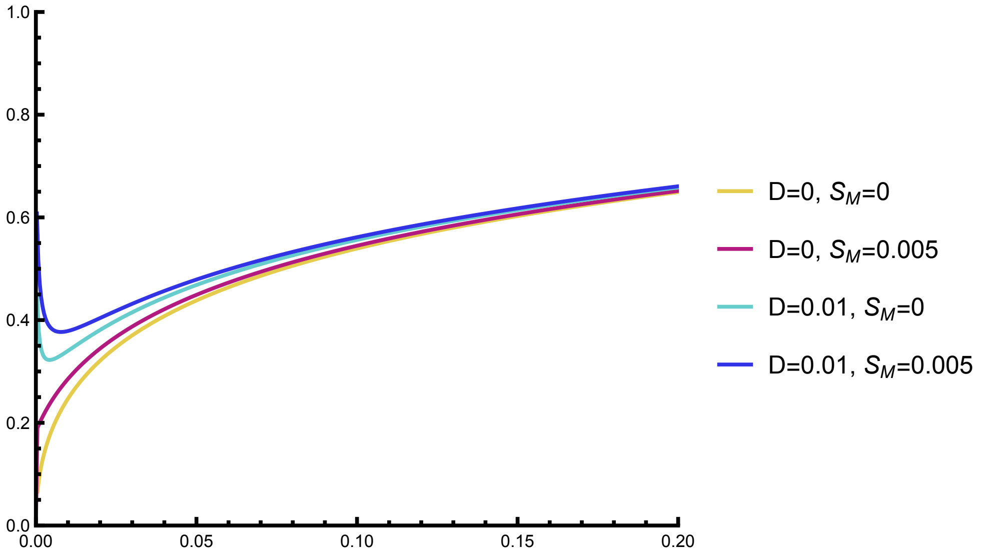

## Fig G: Growth laws_exact_density's effect

```
In[∘]:= useparameters = {ϕS → 0.5, c → 0.8, ϵB → 2, κB → 1, SM → 0.005, D → 0.01};
Show[ListPlot[Table[
    {birthExact /. ϕB → BiosynthesisOptimalExactRelevant [ϵP, ϵB, ϕS, c, D, SM, κB, ρ],
       BiosynthesisOptimalExactRelevant [ϵP, ϵB, ϕS, c, D, SM, κB, ρ]} /.
     useparameters /. ρ → 0.1, {ϵP, 0., 5, 0.001}], Joined → True,
   PlotStyle → ■, AxesStyle → Directive[Black, Thick], AspectRatio → .8,
   PlotRange → {{0, .15}, {0, 0.3}}],
  ListPlot[Table[
    {birthExact /. ϕB → BiosynthesisOptimalExactRelevant [ϵP, ϵB, ϕS, c, D, SM, κB, ρ],
       BiosynthesisOptimalExactRelevant [ϵP, ϵB, ϕS, c, D, SM, κB, ρ]} /.
     useparameters /. ρ → 1, {ϵP, 0, 10, 0.001}],
   Joined → True, PlotStyle → ■, PlotRange → All],
  ListPlot[Table[
    {birthExact /. ϕB → BiosynthesisOptimalExactRelevant [ϵP, ϵB, ϕS, c, D, SM, κB, ρ],
       BiosynthesisOptimalExactRelevant [ϵP, ϵB, ϕS, c, D, SM, κB, ρ]} /.
     useparameters /. ρ → 10, {ϵP, 0.0, 10, 0.001}],
   Joined → True, PlotStyle → ■, PlotRange → All],
  ListPlot[Table[
    {birthExact /. ϕB → BiosynthesisOptimalExactRelevant [ϵP, ϵB, ϕS, c, D, SM, κB, ρ],
       BiosynthesisOptimalExactRelevant [ϵP, ϵB, ϕS, c, D, SM, κB, ρ]} /.
     useparameters /. ρ → 100, {ϵP, .0, 30, 0.001}],
   Joined → True, PlotStyle → ■, PlotRange → All]
  (*ListPlot[Table[{birthExact /.BiosynthesisOptimalExactrelevant[ϵP,ϵB/.ϵB→2],
        ϕB/.BiosynthesisOptimalExactrelevant[ϵP,ϵB/.ϵB→2]}/.useparameters/.
     SM→0.5/.D→0.5,{ϵP,.8,30,0.01}],Joined→True,
   PlotStyle→{RGBColor[.2,.2,.1],Dashing[0.01]}],*)

]
```

Power: Infinite expression $\frac{1}{0.}$ encountered.

Infinity: Indeterminate expression 0. ComplexInfinity encountered.

Power: Infinite expression $\frac{1}{0.}$ encountered.

Infinity: Indeterminate expression 0. ComplexInfinity encountered.

Power: Infinite expression $\frac{1}{0.}$ encountered.

General: Further output of Power::infy will be suppressed during this calculation.

Infinity: Indeterminate expression 0. ComplexInfinity encountered.

General: Further output of Infinity::indet will be suppressed during this calculation.

Out[ ]=

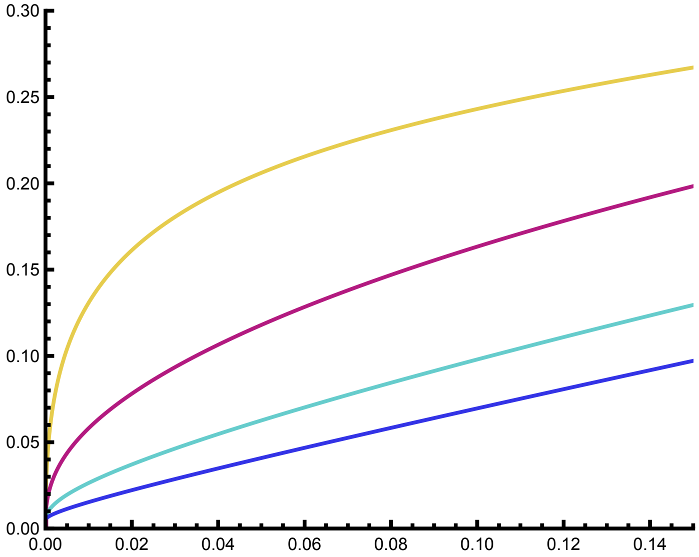

In[ ]:=

```
useparameters = {ϕS → 0.5, c → 0.8, ϵB → 2, κB → 1, SM → 0.005, D → 0.01};
Show[ListPlot[Table[
    {birthExact /. ϕB → BiosynthesisOptimalExactRelevant [ϵP, ϵB, ϕS, c, D, SM, κB, ρ],
       SaturationOptimalExact [ϵP, ϵB, ϕS, c, D, SM, κB, ρ]} /. useparameters /.
     ρ → 0.1, {ϵP, 0., 25, 0.001}], Joined → True, PlotStyle → ■,
  AxesStyle → Directive[Black, Thick], AspectRatio → .8,
  PlotRange → {{0, .3}, {0, 1}}, PlotLegends → {"κB/ρ=10"}],
 ListPlot[Table[
    {birthExact /. ϕB → BiosynthesisOptimalExactRelevant [ϵP, ϵB, ϕS, c, D, SM, κB, ρ],
       SaturationOptimalExact [ϵP, ϵB, ϕS, c, D, SM, κB, ρ]} /. useparameters /. ρ → 1,
    {ϵP, 0, 10, 0.001}], Joined → True, PlotStyle → ■,
  PlotRange → All, PlotLegends → {"κB/ρ=1"}],
 ListPlot[Table[
    {birthExact /. ϕB → BiosynthesisOptimalExactRelevant [ϵP, ϵB, ϕS, c, D, SM, κB, ρ],
       SaturationOptimalExact [ϵP, ϵB, ϕS, c, D, SM, κB, ρ]} /. useparameters /. ρ → 10,
    {ϵP, 0.0, 10, 0.001}], Joined → True, PlotStyle → ■,
  PlotRange → All, PlotLegends → {"κB/ρ=0.1"}],
 ListPlot[Table[
    {birthExact /. ϕB → BiosynthesisOptimalExactRelevant [ϵP, ϵB, ϕS, c, D, SM, κB, ρ],
       SaturationOptimalExact [ϵP, ϵB, ϕS, c, D, SM, κB, ρ]} /. useparameters /.
     ρ → 100, {ϵP, .0, 30, 0.001}], Joined → True, PlotStyle → ■,
  PlotRange → All, PlotLegends → {"κB/ρ=0.01"}]
 (*ListPlot[Table[{birthExact /.BiosynthesisOptimalExactrelevant[ϵP,ϵB/.ϵB→2],
       ϕB/.BiosynthesisOptimalExactrelevant[ϵP,ϵB/.ϵB→2]}/.useparameters/.
     SM→0.5/.D→0.5,{ϵP,.8,30,0.01}],Joined→True,
  PlotStyle→{RGBColor[.2,.2,.1],Dashing[0.01]}],*)

]
```

Power: Infinite expression 1/0. encountered.

Infinity: Indeterminate expression 0. ComplexInfinity encountered.

Power: Infinite expression 1/0. encountered.

Infinity: Indeterminate expression 0. ComplexInfinity encountered.

Power: Infinite expression 1/0. encountered.

General: Further output of Power::infy will be suppressed during this calculation.

••• Infinity: Indeterminate expression 0. ComplexInfinity encountered.

••• General: Further output of Infinity::indet will be suppressed during this calculation.

Out[ ]=

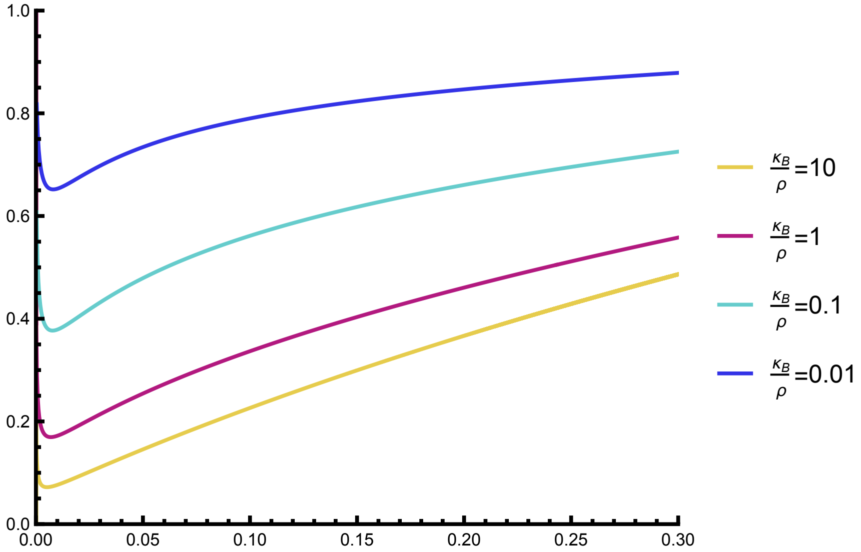

## Fig A: Growth laws_altering $\epsilon_B$

In[ ]:=
```
useparameters = {ϕS → 0.5, c → .8, ϵP → 2};
Show[
 ListPlot[Table[{birthOptimal /. δ → ϵB/ϵP, ϕB /. biosynthesisOptimal[[1]] /. δ → ϵB/ϵP} /.
     useparameters /. SM → 0 /. D → 0, {ϵB, 0.00, 5, 0.001}],
  Joined → True, PlotStyle → ■, AxesStyle → Directive[Black, Thick],
  AspectRatio → .8, PlotRange → All],
 ListPlot[Table[
   {birthOptimal /. δ → ϵB/ϵP, ϕB /. biosynthesisOptimal[[1]] /. δ → ϵB/ϵP} /. useparameters /.
    SM → 0.05 /. D → 0, {ϵB, 0., 10, 0.001}],
  Joined → True, PlotRange → All, PlotStyle → ■, PlotRange → All],
 ListPlot[Table[
   {birthOptimal /. δ → ϵB/ϵP, ϕB /. biosynthesisOptimal[[1]] /. δ → ϵB/ϵP} /. useparameters /.
    SM → 0 /. D → .1, {ϵB, 0.205, 10, 0.001}],
  Joined → True, PlotStyle → ■, PlotRange → All],
 ListPlot[Table[
   {birthOptimal /. δ → ϵB/ϵP, ϕB /. biosynthesisOptimal[[1]] /. δ → ϵB/ϵP} /. useparameters /.
    SM → 0.05 /. D → 0.1, {ϵB, 0.215, 10, 0.001}],
  Joined → True, PlotStyle → ■, PlotRange → All],
 (*ListPlot[Table[
   {birthOptimalTaylor1/.δ →ϵB/ϵP,biosynthesisOptimalTaylor1/.δ →ϵB/ϵP}/.useparameters/.
    SM→0.5/.D→0.5,{ϵP,.8,30,0.01}],
  Joined→True,PlotStyle→{RGBColor[.2,.2,.1],Dashing[0.01]}],*)
 PlotRange → {{0, 0.3}, {0, 0.5}}
]
```

Power: Infinite expression $\frac{1}{0.^2}$ encountered.

Power: Infinite expression $\frac{1}{0.}$ encountered.

Power: Infinite expression $\frac{1}{0.^2}$ encountered.

General: Further output of Power::infy will be suppressed during this calculation.

Out[ ]=

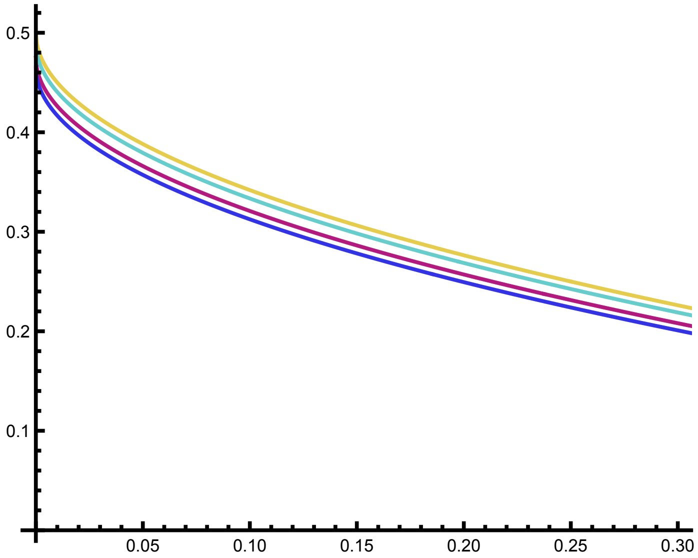


In[ ]:= (*Calculating the $\epsilon_P$ which results in birth rate being equal to zero*)

$$\frac{D\,\epsilon_P}{-D + c\,D - S_M + \epsilon_P - \epsilon_P\,\phi_S} \;/.\; \{S_M \to 0.0,\ D \to 0.1,\ \phi_S \to 0.5,\ c \to .8,\ \epsilon_P \to 2\}$$

Out[ ]=

0.204082

```
In[•]:= useparameters = {ϕS → 0.2, c → .8, ϵP → 2};
Show[ListPlot[
   Table[{birthOptimal /. δ → ϵB/ϵP, saturationOptimal[[1]] /. δ → ϵB/ϵP} /. useparameters /.
      SM → 0 /. D → 0, {ϵB, 0.001, 5, 0.001}],
   Joined → True, PlotStyle → ■, AxesStyle → Directive[Black, Thick],
   AspectRatio → .8, PlotLegends → {"D=0, SM=0"}],
  ListPlot[
   Table[{birthOptimal /. δ → ϵB/ϵP, saturationOptimal[[1]] /. δ → ϵB/ϵP} /. useparameters /.
      SM → 0.05 /. D → 0, {ϵB, 0.001, 10, 0.001}], Joined → True,
   PlotRange → All, PlotStyle → ■, PlotLegends → {"D=0, SM=0.05"}],
  ListPlot[
   Table[{birthOptimal /. δ → ϵB/ϵP, saturationOptimal[[1]] /. δ → ϵB/ϵP} /. useparameters /.
      SM → 0 /. D → .1, {ϵB, 0.0, 10, 0.001}],
   Joined → True, PlotStyle → ■, PlotLegends → {"D=0.1, SM=0"}],
  ListPlot[
   Table[{birthOptimal /. δ → ϵB/ϵP, saturationOptimal[[1]] /. δ → ϵB/ϵP} /. useparameters /.
      SM → 0.05 /. D → 0.1, {ϵB, .05, 30, 0.001}], Joined → True,
   PlotRange → All, PlotStyle → ■, PlotLegends → {"D=0.1, SM=0.05"}],
  PlotRange → {{0, 0.1}, {0.6, 1}}, Axes → True
]
```

••• Power: Infinite expression $\frac{1}{0.^2}$ encountered.

••• Power: Infinite expression $\frac{1}{0.^2}$ encountered.

••• Power: Infinite expression $\frac{1}{0.^2}$ encountered.

••• General: Further output of Power::infy will be suppressed during this calculation.

Out[∘]=

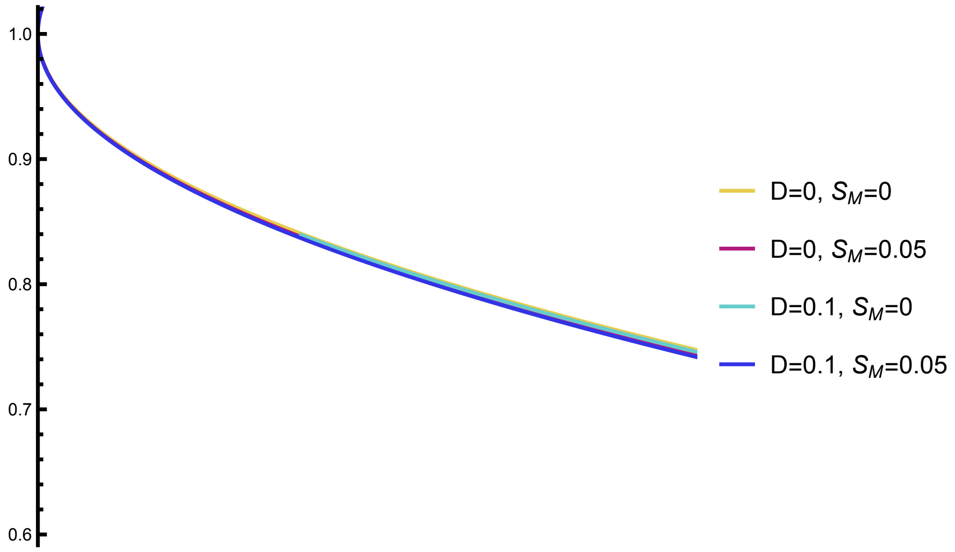


## Fig F: Heat maps for the slope at slow growth

In[∘]:= $\text{biosynthesisOptimal} \;/.\; \delta \to \frac{\epsilon_B}{\epsilon_P} \;/.\; \epsilon_B \to \epsilon B \;/.\; \epsilon_P \to \epsilon P \;/.\; \phi_S \to \phi S \;/.\; S_M \to SM$

Out[∘]=

$$\left\{\phi_B \to \frac{D - c\,D + SM - \epsilon P + \sqrt{\left(D - \frac{c\,D\,\epsilon B}{\epsilon P} + \frac{SM\,\epsilon B}{\epsilon P} + \epsilon B\,(-1 + \phi S)\right)\,(D - c\,D + SM + \epsilon P\,(-1 + \phi S))} + \epsilon P\,\phi S}{\left(-1 + \frac{\epsilon B}{\epsilon P}\right)\,\epsilon P}\right\}$$

In[∘]:= Clear[BiosynthesisOptimal];
BiosynthesisOptimal [εP_, εB_, ϕS_, c_, D_, SM_] :=
BiosynthesisOptimal [εP, εB, ϕS, c, D, SM] =

$$\frac{D - c\,D + SM - \epsilon P + \sqrt{\left(D - \frac{c\,D\,\epsilon B}{\epsilon P} + \frac{SM\,\epsilon B}{\epsilon P} + \epsilon B\,(-1 + \phi S)\right)\,(D - c\,D + SM + \epsilon P\,(-1 + \phi S))} + \epsilon P\,\phi S}{\left(-1 + \frac{\epsilon B}{\epsilon P}\right)\,\epsilon P}$$

In[∘]:= $\text{birthOptimal} \;/.\; \delta \to \frac{\epsilon_B}{\epsilon_P} \;/.\; \epsilon_B \to \epsilon B \;/.\; \epsilon_P \to \epsilon P \;/.\; \phi_S \to \phi S \;/.\; S_M \to SM$

Out[∘]=

$$-\frac{1}{\left(-1 + \frac{\epsilon B}{\epsilon P}\right)^2}\left(D + \frac{D\,\epsilon B^2}{\epsilon P^2} - \frac{c\,D\,\epsilon B^2}{\epsilon P^2} - \frac{c\,D\,\epsilon B}{\epsilon P} + \frac{SM\,\epsilon B\,\left(1 + \frac{\epsilon B}{\epsilon P}\right)}{\epsilon P} + \epsilon B\left(1 + \frac{\epsilon B}{\epsilon P}\right)(-1 + \phi S) + \frac{2\,\epsilon B\,\sqrt{((-1 + c)\,D - SM - \epsilon P\,(-1 + \phi S))\,\left(D\left(-1 + \frac{c\,\epsilon B}{\epsilon P}\right) - \frac{SM\,\epsilon B}{\epsilon P} + \epsilon P\left(\frac{\epsilon B}{\epsilon P} - \frac{\epsilon B\,\phi S}{\epsilon P}\right)\right)}}{\epsilon P}\right)$$

In[ ]:= Clear[BirthOptimal];
BirthOptimal[ϵP_, ϵB_, ϕS_, c_, D_, SM_] :=
BirthOptimal[ϵP, ϵB, ϕS, c, D, SM] =

$$-\frac{1}{\left(-1+\frac{\epsilon B}{\epsilon P}\right)^2}\left(D+\frac{D\,\epsilon B^2}{\epsilon P^2}-\frac{c\,D\,\epsilon B^2}{\epsilon P^2}-\frac{c\,D\,\epsilon B}{\epsilon P}+\frac{SM\,\epsilon B\left(1+\frac{\epsilon B}{\epsilon P}\right)}{\epsilon P}+\epsilon B\left(1+\frac{\epsilon B}{\epsilon P}\right)(-1+\phi S)+\frac{2\,\epsilon B\sqrt{((-1+c)\,D-SM-\epsilon P\,(-1+\phi S))\left(D\left(-1+\frac{c\,\epsilon B}{\epsilon P}\right)-\frac{SM\,\epsilon B}{\epsilon P}+\epsilon P\left(\frac{\epsilon B}{\epsilon P}-\frac{\epsilon B\,\phi S}{\epsilon P}\right)\right)}}{\epsilon P}\right)$$

In[ ]:= Clear[slopeFinder];
slopeFinder[ϵP_, ϵB_, ϕS_, c_, D_, SM_, δ_] :=
slopeFinder[ϵP, ϵB, ϕS, c, D, SM, δ] =
(BiosynthesisOptimal[ϵP + δ, ϵB, ϕS, c, D, SM] -
BiosynthesisOptimal[ϵP, ϵB, ϕS, c, D, SM]) /
(BirthOptimal[ϵP + δ, ϵB, ϕS, c, D, SM] - BirthOptimal[ϵP, ϵB, ϕS, c, D, SM])

In[ ]:= Clear[Concavity];
Concavity[ϵP0_, ϵB_, ϕS_, c_, D_, SM_, δ_] :=
Concavity[ ϵP0, ϵB, ϕS, c, D, SM, δ] =

$$\frac{\text{slopeFinder}[\epsilon P,\ \epsilon B,\ \phi S,\ c,\ D,\ SM,\ \delta]}{\text{slopeFinder}[20,\ \epsilon B,\ \phi S,\ c,\ D,\ SM,\ \delta]}\ /.\ \epsilon P\to\frac{((-1+c)\,D-SM)\,\epsilon B}{D+\epsilon B\,(-1+\phi S)}+\epsilon P0$$

In[ ]:=
```
useparameters = {ϵP0 → 0.1, ϵB → 3, ϕS → 0.5};
Table[
 Labeled[DensityPlot[Concavity[ϵP0, ϵB, ϕS, c, D, SM, 0.01] /. useparameters,
   {SM, 0, 0.5}, {D, 0, .5}, Ticks → None, MeshFunctions → {#3 &}, Mesh → {{0, 1}},
   ImageSize → Small, MeshStyle → Directive[Dashed, GrayLevel[0.3]],
   Exclusions → None, PlotLegends → Automatic,
   ColorFunction → (ColorData["SunsetColors"][#] &), ColorFunctionScaling → True,
   FrameLabel → {"Maintenance demands(S_M)", "Degradation(D)"}],
  "c=" <> ToString[c], Top], {c, {0, 0.5, 0.85, 1}}]
```

Out[ ]=

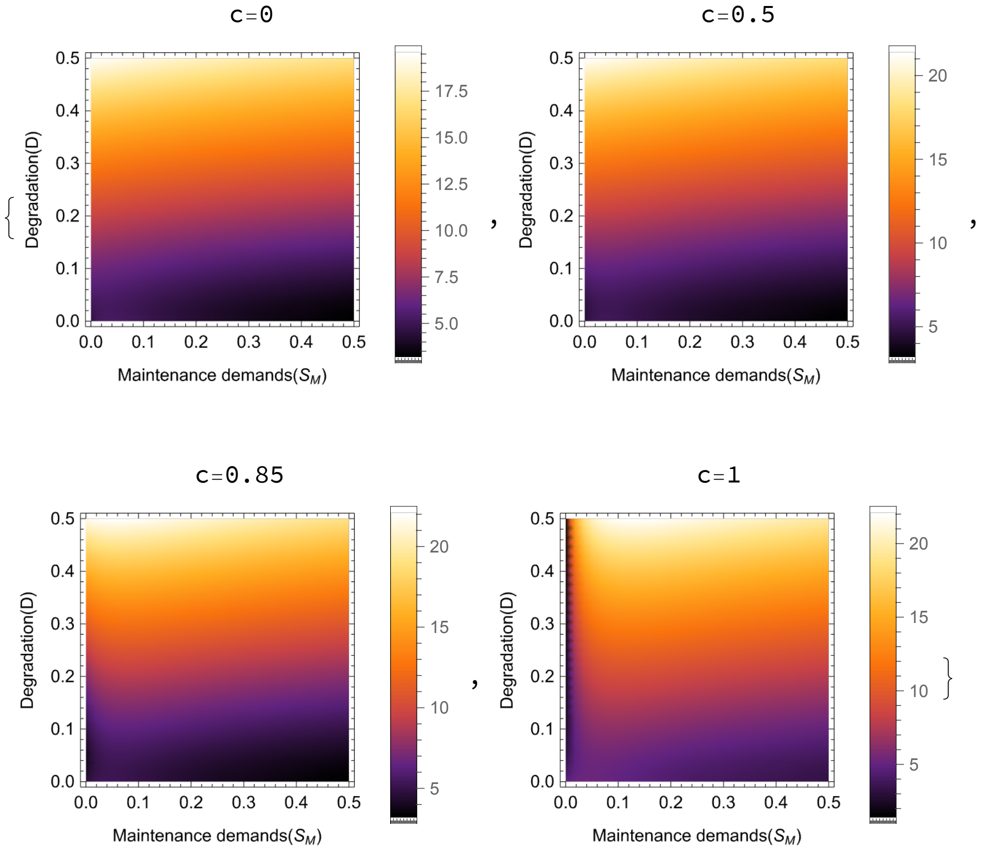


## Fig E: Heat maps for the slope at slow growth_Exact

In[ ]:=
```
Clear[slopeFinderExact];
slopeFinderExact[ϵP_, ϵB_, ϕS_, c_, D_, SM_, κB_, ρ_, δ_] :=
 slopeFinderExact[ϵP, ϵB, ϕS, c, D, SM, κB, ρ, δ] =
  (BiosynthesisOptimalExactRelevant[ϵP + δ, ϵB, ϕS, c, D, SM, κB, ρ] -
     BiosynthesisOptimalExactRelevant[ϵP, ϵB, ϕS, c, D, SM, κB, ρ]) /
   (BirthOptimalExact[ϵP + δ, ϵB, ϕS, c, D, SM, κB, ρ] -
     BirthOptimalExact[ϵP, ϵB, ϕS, c, D, SM, κB, ρ])
```

In[∘]:=
```
slopeFinderExact [1, 2, 0.5, 0.85, 0.01, 0.05, 1, 1, 0.01]
```
Out[∘]=
```
0.692924
```

In[∘]:=
```
Clear[ConcavityExact];
ConcavityExact[ϵP0_, ϵB_, ϕS_, c_, D_, SM_, ϰB_, ρ_, δ_] :=
 ConcavityExact[ ϵP0, ϵB, ϕS, c, D, SM, ϰB, ρ, δ] =
```
$$\frac{\text{slopeFinderExact}[\epsilon P, \epsilon B, \phi S, c, D, SM, \varkappa B, \rho, \delta]}{\text{slopeFinderExact}[20, \epsilon B, \phi S, c, D, SM, \varkappa B, \rho, \delta]} \;/.\; \epsilon P \to \frac{((-1 + c)\, D - SM)\, \epsilon B}{D + \epsilon B\, (-1 + \phi S)} + \epsilon P0$$

In[∘]:=
```
ConcavityExact [1, 2, 0.5, 0.85, 0.01, 0.05, 1, 1, 0.01]
```
Out[∘]=
```
1.98917
```

In[∘]:=
```
useparameters = {ϵP0 → 0.1, ϵB → 3, ϕS → 0.5, ϰB → 1, ρ → 10};
Table[Labeled[
  DensityPlot[ConcavityExact[ϵP0, ϵB, ϕS, c, D, SM, ϰB, ρ, 0.01] /. useparameters,
   {SM, 0, 0.5}, {D, 0, .5}, Ticks → None, MeshFunctions → {#3 &}, Mesh → {{0, 1}},
   ImageSize → Small, MeshStyle → Directive[Dashed, GrayLevel[0.3]],
   Exclusions → None, PlotLegends → Automatic,
   ColorFunction → (ColorData["SunsetColors"][#] &), ColorFunctionScaling → True,
   FrameLabel → {"Maintenance demands(S_M)", "Degradation(D)"}],
  "c=" <> ToString[c], Top], {c, {0, 0.5, 0.85, 1}}]
```

Out[∘]=

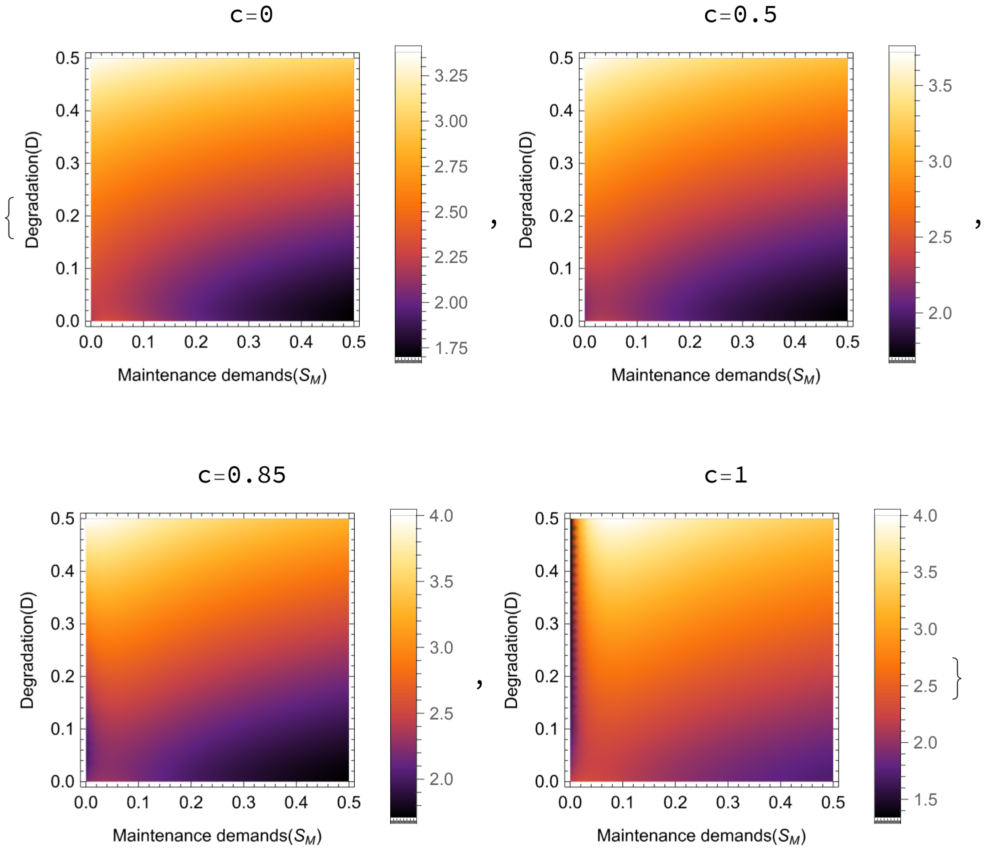

Supplement: Supplementary file 3 [file pbio.3003995.s002.zip › S1 File/Supplementary_Mathematica_2026_09_14.pdf]
