# Supplementary material for: Bridging energy and proteome allocation models allows prediction of the resource cost of traits across environments
Source: PLoS Biol. 2026 Oct 13;24(10):e3003995. doi: 10.1371/journal.pbio.3003995 (PMC13660896; doi:10.1371/journal.pbio.3003995)
Supplement: Peer review history

# Editor report: Decision Letter 0

**DOI:** 10.1371/journal.pbio.3003995.r001

**Authors:** Ankiit Ahluwalia

28 Jan 2026

Dear Dr Meghrazi,

Thank you for submitting your manuscript entitled "Bridging energy and proteome allocation models to predict the resource cost of traits in different environments" for consideration as a Research Article by PLOS Biology.

Your manuscript has now been evaluated by the PLOS Biology editorial staff (as well as by an academic editor with relevant expertise) and I am writing to let you know that we would like to send your submission out for external peer review.

However, before we can send your manuscript to reviewers, we need you to complete your submission by providing the metadata that is required for full assessment. To this end, please login to Editorial Manager where you will find the paper in the 'Submissions Needing Revisions' folder on your homepage. Please click 'Revise Submission' from the Action Links and complete all additional questions in the submission questionnaire.

Once your full submission is complete, your paper will undergo a series of checks in preparation for peer review. After your manuscript has passed the checks it will be sent out for review. To provide the metadata for your submission, please Login to Editorial Manager (https://www.editorialmanager.com/pbiology) within two working days, i.e. by Jan 30 2026 11:59PM.

If your manuscript has been previously peer-reviewed at another journal, PLOS Biology is willing to work with those reviews in order to avoid re-starting the process. Submission of the previous reviews is entirely optional and our ability to use them effectively will depend on the willingness of the previous journal to confirm the content of the reports and share the reviewer identities. Please note that we reserve the right to invite additional reviewers if we consider that additional/independent reviewers are needed, although we aim to avoid this as far as possible. In our experience, working with previous reviews does save time.

If you would like us to consider previous reviewer reports, please edit your cover letter to let us know and include the name of the journal where the work was previously considered and the manuscript ID it was given. In addition, please upload a response to the reviews as a 'Prior Peer Review' file type, which should include the reports in full and a point-by-point reply detailing how you have or plan to address the reviewers' concerns.

During the process of completing your manuscript submission, you will be invited to opt-in to posting your pre-review manuscript as a bioRxiv preprint. Visit http://journals.plos.org/plosbiology/s/preprints for full details. If you consent to posting your current manuscript as a preprint, please upload a single Preprint PDF.

Feel free to email us at plosbiology@plos.org if you have any queries relating to your submission.

Kind regards,

Ankiit

Ankiit Ahluwalia,

PLOS Biology

aahluwalia@plos.org

# Peer review: Decision Letter 1

**DOI:** 10.1371/journal.pbio.3003995.r002

**Authors:** Ankiit Ahluwalia

25 Mar 2026

Dear Dr Meghrazi,

Thank you for your patience while your manuscript "Bridging energy and proteome allocation models to predict the resource cost of traits in different environments" was peer-reviewed at PLOS Biology. It has now been evaluated by the PLOS Biology editors, an Academic Editor with relevant expertise, and by several independent reviewers.

In light of the reviews, which you will find at the end of this email, we would like to invite you to revise the work to thoroughly address the reviewers' reports.

As you will see below, both reviewers find the study potentially interesting, however they also raise a number of concerns and they offer suggestions to strengthen the study further. Overall, these concerns are related to providing greater clarification, reframing and more detailed description of the assumptions and the model that need to be addressed before we can consider your study for publication.

After discussing these comments with the Academic Editor and the rest of the team, we have decided to invite a Major revision that should address all the points raised by the reviewers.

Given the extent of revision needed, we cannot make a decision about publication until we have seen the revised manuscript and your response to the reviewers' comments. Your revised manuscript is likely to be sent for further evaluation by all or a subset of the reviewers.

In addition to these revisions, you will need to complete some formatting changes, which you will receive in a follow up email. A member of our team will be in touch with a set of requests shortly.

We expect to receive your revised manuscript within 3 months. Please email us (plosbiology@plos.org) if you have any questions or concerns, or would like to request an extension.

At this stage, your manuscript remains formally under active consideration at our journal; please notify us by email if you do not intend to submit a revision so that we may withdraw it.

**IMPORTANT - SUBMITTING YOUR REVISION**

Your revisions should address the specific points made by each reviewer. Please submit the following files along with your revised manuscript:

1. A 'Response to Reviewers' file - this should detail your responses to the editorial requests, present a point-by-point response to all of the reviewers' comments, and indicate the changes made to the manuscript.

*NOTE: In your point-by-point response to the reviewers, please provide the full context of each review. Do not selectively quote paragraphs or sentences to reply to. The entire set of reviewer comments should be present in full and each specific point should be responded to individually, point by point.

You should also cite any additional relevant literature that has been published since the original submission and mention any additional citations in your response.

2. In addition to a clean copy of the manuscript, please also upload a 'track-changes' version of your manuscript that specifies the edits made. This should be uploaded as a "Revised Article with Changes Highlighted" file type.

*Re-submission Checklist*

When you are ready to resubmit your revised manuscript, please refer to this re-submission checklist: https://plos.io/Biology_Checklist

To submit a revised version of your manuscript, please go to https://www.editorialmanager.com/pbiology/ and log in as an Author. Click the link labelled 'Submissions Needing Revision' where you will find your submission record.

Please make sure to read the following important policies and guidelines while preparing your revision:

*Published Peer Review*

Please note while forming your response, if your article is accepted, you may have the opportunity to make the peer review history publicly available. The record will include editor decision letters (with reviews) and your responses to reviewer comments. If eligible, we will contact you to opt in or out. Please see here for more details:

https://blogs.plos.org/plos/2019/05/plos-journals-now-open-for-published-peer-review/

*PLOS Data Policy*

Please note that as a condition of publication PLOS' data policy (http://journals.plos.org/plosbiology/s/data-availability) requires that you make available all data used to draw the conclusions arrived at in your manuscript. If you have not already done so, you must include any data used in your manuscript either in appropriate repositories, within the body of the manuscript, or as supporting information (N.B. this includes any numerical values that were used to generate graphs, histograms etc.). For an example see here: http://www.plosbiology.org/article/info%3Adoi%2F10.1371%2Fjournal.pbio.1001908#s5

*Blot and Gel Data Policy*

We require the original, uncropped and minimally adjusted images supporting all blot and gel results reported in an article's figures or Supporting Information files. We will require these files before a manuscript can be accepted so please prepare them now, if you have not already uploaded them. Please carefully read our guidelines for how to prepare and upload this data: https://journals.plos.org/plosbiology/s/figures#loc-blot-and-gel-reporting-requirements

*Protocols deposition*

To enhance the reproducibility of your results, we recommend that if applicable you deposit your laboratory protocols in protocols.io, where a protocol can be assigned its own identifier (DOI) such that it can be cited independently in the future. Additionally, PLOS ONE offers an option for publishing peer-reviewed Lab Protocol articles, which describe protocols hosted on protocols.io. Read more information on sharing protocols at https://plos.org/protocols?utm_medium=editorial-email&utm_source=authorletters&utm_campaign=protocols

Thank you again for your submission to our journal. We hope that our editorial process has been constructive thus far, and we welcome your feedback at any time. Please don't hesitate to contact us if you have any questions or comments.

Sincerely,

Ankiit

Ankiit Ahluwalia,

PLOS Biology

aahluwalia@plos.org

------------------------------------

REVIEWS:

Reviewer #1: To understand how specific cellular traits evolve and why they persist, it is important to account for both benefits and costs, to obtain a net benefit. The introduction of a new trait is almost always a drain on one or more cellular resources. This could be the cell energy budget or biosynthesis capacity, but also the cell volume, surface area, or the availability of specific limiting elements like N, P, or Fe. It has remained a puzzle how drains on a combination of these various resources collectively affect the growth rate (and/or survival) of a cell, and therefore it is unclear what cost is associated with the introduction of a new cellular trait.

In the present work, the authors tackle this important problem and present a theoretical framework that accounts for both the cell energy budget and the biosynthesis capacity. They build on the proteome allocation model approach that has been used in the past by various other authors. Such models typically subdivide the proteome in a fraction that produces proteins, a fraction that produces substrates, and an "other" fraction that houses all other processes. The authors present a similar model but include (1) a drain on the substrate pool that represents maintenance costs, (2) a drain on the macromolecule pool that represents degradation (with an adjustable degree of recycling substrates), and (3) a degree of saturation of the biosynthesis machinery that depends (directly or indirectly) on the pool of substrates. Addition (1) and (2) introduce an important aspect of energy allocation models into the proteome allocation model.

The resulting model can be used to predict the optimal partitioning between the two nonstructural fractions (biosynthesis and provisioning) as well as the birthrate at this optimum. The model can also be used to examine and the consequences of various alterations to the parameters to this optimal partitioning and the birthrate. The structural fraction (ϕS), degradation rate (D), and maintenance demands (SM) are parameters of particular importance in analyzing the fitness consequences of adding a new cellular trait, and it is revealed in this study that these have rather different consequences. It is also revealed that the impact of various cellular alterations depends on the starting size of the fractions that are affected by these alterations, a finding that is intuitive after reading through the analysis presented in this work.

I recommend publishing this paper, with minor adjustments.

Issues that need to be addressed:

1)It is stated in line 92 that "we include all macromolecules in our model rather than just proteins". This is mentioned as one of the key differences between the present model and the previous proteome allocation models. However, it is not clear how the previous models specifically include proteins and how this model includes also other macromolecules. Is this related to the parameter values? Or, is there a structural difference in the model that accounts for this difference? Since this is one of the three key differences between this and previous work, this needs to be clarified.

2)There appears to be a mismatch by the rate of substrates produced by the provisioning fraction and the rate of substrates utilized by the biosynthesis fraction. I calculate the substrate production rate from the equation on line 118 and the biosynthesis rate from the equation on line 120, finding that substrate production is ~2.4-fold higher than its use in biosynthesis at the optimal biosynthesis fraction (using eq 1, parameters as in Fig 3a). This means that either 60% of all substrates that are produced are lost, or that these form a really large pool of substrates in the cell, or some combination of the two. Whether this is really a problem depends on what the physiological ranges of the other parameters are, as the quantity of the excess substrate production is affected by the parameter choices. Perhaps the authors can explain this discrepancy and offer a reason for why this is not a problem. If no such reason can be given there should perhaps be an additional constraint in the model that includes the size of the substrate pool, for example by making substrates a separate biomass fraction.

3)The description of the energy allocation model is incomplete as it contains only the maintenance aspect. It is stated that "Two different frameworks have been proposed to quantify the resource costs of traits, focusing on either energy allocation or proteome allocation. These frameworks implicitly assume energy provisioning or protein production limits growth, respectively". The energy allocation framework used by Lynch and Marinov (2015) contains both a maintenance cost component and a growth (or construction) cost component. The maintenance cost component functions as the authors present. However, the growth cost component probably functions more like a proteome allocation model. This is because the growth costs are mostly opportunity costs, i.e. food molecules diverted to making carbon backbones for amino acids in proteins, etc. The difference between the proteome allocation model and the energy allocation model being that in proteome allocation models, biosynthesis is modeled explicitly whereas in the energy allocation models biosynthesis is implicit. Despite this quibble, I think the authors made a novel and important contribution in uniting the two perspectives.

Minor issues/comments (it is up to the authors if they want to address these):

1)An assumption in the energetic cost models is that the relative energetic cost scales linearly with the fitness penalty (equal to the relative depression in growth rate if differences in survival are not relevant). A proteome allocation model as presented here could be used to test this assumption. This is already mentioned in the text in the discussion, e.g. lines 354-373. However, this only examines maintenance costs. To examine the effect of what in the energy allocation models is called growth or construction cost (mostly opportunity costs) on fitness one would have to examine the effect of a change in the structural fraction (from a starting point of ϕS=0). When I examine this using Eq 8 it appears that there is a linear relation between ΔϕS and the birth rate under optimal allocation. But this is for the Taylor expansion (Eq 8) and I'm curious to see if the full model yields the same linear result. It would be interesting to see a plot of birth rate as a function of the structural fraction. Examining the effect of SM in the same way reveals that its relationship with ΔϕS is more complicated (as was already mentioned by the authors in the discussion).

2)For the claim in the abstract that "increasing food quality increases the cost of traits that require the production of new structures", would it still be true if a relative birth rate is examined rather than an absolute one.

3)On lines 138 and 139 it is stated that "As the biosynthesis fraction increases, there are fewer provisioning enzymes to supply each biosynthetic enzyme, and the biosynthesis saturation level decreases". Can't a small provisioning fraction with a large efficiency not supply enough substrates to saturate biosynthesis?

4)One wonders if we should expect a hyperbolic function relating the substrate concentration to the biosynthesis rate since there are multiple steps between an amino acid just having been produced and protein chain elongation (i.e. tRNA charging, elongation factor binding, and ternary complex binding to ribosomes). My suspicion is that the assumption of a hyperbolic relation is fine because even whole cell growth rate is related to external nutrient concentration by a hyperbolic function.

5)The level of activity of the biosynthesis fraction is affected by the substrate pool, and thereby by the provisioning fraction. However, the reverse could also be true that removal of substrates by the biosynthesis fraction could increase the flux through the provisioning fraction and thereby make the provisioning fraction more efficient. This does not need to be addressed here.

6)I want to draw attention to some literature that deals with similar questions of multiple or other resource types (or constraints). It is up to the authors whether they want to discuss any of this in their paper, but it does present some other directions that the approach of the authors could be extended into in the future. John Raven has written many articles on cost-benefit analyses in which both energy and specific elemental resources are examined though not combined into a single fitness metric (Raven (1987) New Phytol; Raven (2011) Physiol Plant; Raven and Richardson (1984) New Phytol). There is also work on the effects of cell surface area occupation: (Szenk, Dill, and de Graff (2017) Cell Syst; Zhuang, Vemuri, and Mahadevan (2011) Mol Syst Biol) and on the combination of cell surface area and the energetic approach in one fitness calculation (Schavemaker and Lynch (2025) Cell Reports).

7)The ratio kB/ρ has a significant impact on the birth rate and, by using the approximation, is essentially set to 1 in the model in the main text, even though the discussion in the supplement suggests the real value of kB/ρ to be on the order of 0.1. This makes the model not directly applicable to fitness calculations, which is unfortunate but doesn't negate the advances made in this work. This can be addressed in subsequent work by people interested in applying the model to specific biological questions.

8)It may be useful to plot the findings of Fig 4 not just as an absolute change in birth rate but also as a relative change in birth rate, as that is more relevant to evolutionary questions.

9)The terms "environmental quality" and "genetic background" could use some elaboration with biological examples in the introduction.

10)Shouldn't "predicted" in line 313 be "observed"?

11)In the text it is stated that some of the lines in Fig 5a have a non-zero intercept (lines 308-310). This is not obvious from Fig 5a, so it may be helpful to include a zoomed version of Fig 5a at the low birthrate region of the plot that shows the intercepts.

12)The tick labels on some of the axes could be bigger to increase legibility (Fig 3b-f, Fig 4, and Fig 5).

13)In the equation that gives Sm in the "Maintenance demands" section of the supplement there is a typo in Avogadro's constant. (Note also that the line numbering is absent in this section.)

14)In the supplement, line 83 has a typo in the equation. It says "vollume" instead of "volume".

15)In the supplement, line 124 states "the effect" twice.

Reviewer #2: This manuscript proposes a unified macromolecule allocation framework that simultaneously considers energy provisioning and biosynthesis limitations. Addressing this problem is highly relevant and could represent an important contribution to ecological theory. The text is well written and generally clear.

The following are my comments to the authors:

I appreciate the authors' effort to produce a unified framework to understand the cost of diverse traits and to explain observed patterns of bacterial growth. In my view, quantifying cost as an absolute reduction in the birth rate of cells is appropriate, as growth rate is one of the most broadly used measures of Darwinian fitness. However, a crucial assumption in the optimal allocation analysis (fig 3), cost of traits analysis (fig 4), and the bacterial growth laws (fig 5) is that the structural fraction is constant. The manuscript does not present evidence that this assumption holds across environmental conditions or genotypes. Because deviations from this assumption could bias optimal allocations, trait cost predictions, and the correspondence with growth laws, the authors should either provide empirical support for this assumption or explicitly acknowledge this limitation in the discussion.

Another concern is that the framework appears to be formulated primarily for high-quality environments in which food has a stoichiometric composition similar to that of the cells. However, in low-quality environments, for example, those with high carbon availability (e.g., sugars) but limited phosphorus or nitrogen, stoichiometric imbalances could influence how macromolecules are allocated between energy provisioning and biosynthesis. The authors partially mention this limitation in the supplementary material when discussing assumptions of parameter independence. However, I consider this a central limitation of the model that should be discussed in the main text. For this reason, I also find the claim that the model facilitates a synthesis between ecological stoichiometry theory and the metabolic theory of ecology (L349-353) somewhat overstated. Such a synthesis may be better framed as a direction for future work rather than a conclusion of the present study.

Despite these limitations, I value the contribution of this framework. Scientific progress often occurs incrementally, and clearly acknowledging model limitations can help guide future work to address them. In this sense, the present study advances the field by building upon the framework of Scott et al. (2010).

# Author comment: Author response to Decision Letter 2

**DOI:** 10.1371/journal.pbio.3003995.r003

11 Jun 2026

Attachment Submitted filename: MMallocation_plos_reviews.pdf

# Peer review: Decision Letter 2

**DOI:** 10.1371/journal.pbio.3003995.r004

**Authors:** Ankiit Ahluwalia

6 Aug 2026

Dear Dr Meghrazi,

Thank you for your patience while we considered your revised manuscript "Bridging energy and proteome allocation models to predict the resource cost of traits in different environments" for publication as a Research Article at PLOS Biology. This revised version of your manuscript has been evaluated by the PLOS Biology editors, the Academic Editor and the original reviewers.

Based on the reviews, we are likely to accept this manuscript for publication, provided you satisfactorily address the remaining points raised by the reviewers (if any). Please also make sure to address the following data and other policy-related requests.

IMPORTANT - please attend to the following:

a)We noted that you’ve included your Mathematica notebook as a PDF. Please also supply your .nb file (I think that's what they're called). This could either be another supp file, or you could put it on Github/Zenodo and supply the Zenodo URL; either way would work. Because Github depositions can be readily changed or deleted, please make a permanent DOI’d copy (e.g. in Zenodo - see instructions below my signature).

b)Please clearly cite the location of the data in all relevant main and supplementary figure legends. For example, you can add: “The data underlying this figure can be found at doi.org/10.xxx/zenodo.xxxxx.” We understand that this may appear repetitive, but it helps make the figures and their legends more self-contained.

c)In order to make the Title more specific and appealing, please change it to "Bridging energy and proteome allocation models allows prediction of the resource cost of traits across environments."

d)Our Academic editor has advised to adjust figure 3 b-f and the inset of figure 5 to make them readable (especially the axis labels).

As you address these items, please take this last chance to review your reference list to ensure that it is complete and correct. If you have cited papers that have been retracted, please include the rationale for doing so in the manuscript text, or remove these references and replace them with relevant current references. Any changes to the reference list should be mentioned in the cover letter that accompanies your revised manuscript.

In addition to these revisions, you may need to complete some formatting changes, which you will receive in a follow up email. A member of our team will be in touch with a set of requests shortly. If you do not receive a separate email within a few days, please assume that checks have been completed, and no additional changes are required.

We expect to receive your revised manuscript within two weeks.

To submit your revision, please go to https://www.editorialmanager.com/pbiology/ and log in as an Author. Click the link labelled 'Submissions Needing Revision' to find your submission record. Your revised submission must include the following:

- a cover letter that should detail your responses to any editorial requests, if applicable, and whether changes have been made to the reference list

- a Response to Reviewers file that provides a detailed response to the reviewers' comments (if applicable, if not applicable please do not delete your existing 'Response to Reviewers' file.)

- a track-changes file indicating any changes that you have made to the manuscript.

NOTE: If Supporting Information files are included with your article, note that these are not copyedited and will be published as they are submitted. Please ensure that these files are legible and of high quality (at least 300 dpi) in an easily accessible file format. For this reason, please be aware that any references listed in an SI file will not be indexed. For more information, see our Supporting Information guidelines:

https://journals.plos.org/plosbiology/s/supporting-information

*Published Peer Review History*

Please note that you may have the opportunity to make the peer review history publicly available. The record will include editor decision letters (with reviews) and your responses to reviewer comments. If eligible, we will contact you to opt in or out. Please see here for more details:

https://plos.org/published-peer-review-history/

*Press*

Should you, your institution's press office or the journal office choose to press release your paper, please ensure you have opted out of Early Article Posting on the submission form. We ask that you notify us as soon as possible if you or your institution is planning to press release the article.

*Protocols deposition*

To enhance the reproducibility of your results, we recommend that if applicable you deposit your laboratory protocols in protocols.io, where a protocol can be assigned its own identifier (DOI) such that it can be cited independently in the future. Additionally, PLOS ONE offers an option for publishing peer-reviewed Lab Protocol articles, which describe protocols hosted on protocols.io. Read more information on sharing protocols at https://plos.org/protocols?utm_medium=editorial-email&utm_source=authorletters&utm_campaign=protocols

Please do not hesitate to contact me should you have any questions.

Sincerely,

Ankiit

Ankiit Ahluwalia,

aahluwalia@plos.org

PLOS Biology

------------------------------------------------------------------------

****[DELETE AS APPROPRIATE]****

ETHICS STATEMENT:

-- Please include the full name of the IACUC/ethics committee that reviewed and approved the animal care and use protocol/permit/project license. Please also include an approval number.

-- Please include the specific national or international regulations/guidelines to which your animal care and use protocol adhered. Please note that institutional or accreditation organization guidelines (such as AAALAC) do not meet this requirement.

-- Please include information about the form of consent (written/oral) given for research involving human participants. All research involving human participants must have been approved by the authors' Institutional Review Board (IRB) or an equivalent committee, and must have been conducted according to the principles expressed in the Declaration of Helsinki.

------------------------------------------------------------------------

DATA POLICY:

You may be aware of the PLOS Data Policy, which requires that all data be made available without restriction: http://journals.plos.org/plosbiology/s/data-availability. For more information, please also see this editorial: http://dx.doi.org/10.1371/journal.pbio.1001797

Note that we do not require all raw data. Rather, we ask that all individual quantitative observations that underlie the data summarized in the figures and results of your paper be made available in one of the following forms:

1) Supplementary files (e.g., excel). Please ensure that all data files are uploaded as 'Supporting Information' and are invariably referred to (in the manuscript, figure legends, and the Description field when uploading your files) using the following format verbatim: S1 Data, S2 Data, etc. Multiple panels of a single or even several figures can be included as multiple sheets in one excel file that is saved using exactly the following convention: S1_Data.xlsx (using an underscore).

2) Deposition in a publicly available repository. Please also provide the accession code or a reviewer link so that we may view your data before publication.

Regardless of the method selected, please ensure that you provide the individual numerical values that underlie the summary data displayed in the following figure panels as they are essential for readers to assess your analysis and to reproduce it:

[Figs….]

NOTE: the numerical data provided should include all replicates AND the way in which the plotted mean and errors were derived (it should not present only the mean/average values).

Please also ensure that figure legends in your manuscript include information on where the underlying data can be found, and ensure your supplemental data file/s has a legend.

Please ensure that your Data Statement in the submission system accurately describes where your data can be found.

------------------------------------------------------------------------

APPROPRIATE DATA PRESENTATION?

(If you think the paper is not using best practice, add the following)

- Please ensure that you are using best practice for statistical reporting and data presentation. These are our guidelines https://journals.plos.org/plosbiology/s/best-practices-in-research-reporting#loc-statistical-reporting and a useful resource on data presentation https://journals.plos.org/plosbiology/article?id=10.1371/journal.pbio.1002128

- If you are reporting experiments where n ≤ 5, please plot each individual data point.

------------------------------------------------------------------------

CODE POLICY

Per journal policy, if you have generated any custom code during the course of this investigation, please make it available without restrictions. Please ensure that the code is sufficiently well documented and reusable, and that your Data Statement in the Editorial Manager submission system accurately describes where your code can be found. More information on our Code Policy, what and how to share can be found here: https://journals.plos.org/plosbiology/s/code-availability

Please note that we cannot accept sole deposition of code in GitHub, as this could be changed after publication. However, you can archive this version of your publicly available GitHub code to Zenodo. Once you do this, it will generate a DOI number, which you will need to provide in the Data Accessibility Statement (you are welcome to also provide the GitHub access information). See the process for doing this here: https://docs.github.com/en/repositories/archiving-a-github-repository/referencing-and-citing-content

------------------------------------------------------------------------

SPECIES INDICATED IN THE ABSTRACT?

- Please note that per journal policy, the model system/species studied should be clearly stated in the abstract of your manuscript.

**EDITOR, CONSIDER ALSO INCLUDING ORGANISM IN THE TITLE, IF SENSICAL/RELEVANT AND/OR TO AVOID BEING MISLEADING/OVERREACHING (not always preferred)**

------------------------------------------------------------------------

BLOT AND GEL REPORTING REQUIREMENTS:

We require the original, uncropped and minimally adjusted images supporting all blot and gel results reported in an article's figures or Supporting Information files. We will require these files before a manuscript can be accepted so please prepare and upload them now. Please carefully read our guidelines for how to prepare and upload this data: https://journals.plos.org/plosbiology/s/figures#loc-blot-and-gel-reporting-requirements

------------------------------------------------------------------------

DATA NOT SHOWN?

- Please note that per journal policy, we do not allow the mention of "data not shown", "personal communication", "manuscript in preparation" or other references to data that is not publicly available or contained within this manuscript. Please either remove mention of these data or provide figures presenting the results and the data underlying the figure(s).

------------------------------------------------------------------------

Reviewer remarks:

Reviewer #1: The authors have addressed my comments on their paper to my satisfaction and, as far as I'm concerned, the paper is acceptable for publication.

Reviewer #2: The authors have addressed my comments in the new version. I recommend the new version for publication.

# Author comment: Author response to Decision Letter 3

**DOI:** 10.1371/journal.pbio.3003995.r005

20 Aug 2026

Attachment Submitted filename: MMallocation_plos_reviews_auresp_3.pdf

# Editor report: Decision Letter 3

**DOI:** 10.1371/journal.pbio.3003995.r006

**Authors:** Ankiit Ahluwalia

28 Aug 2026

Dear Dr Meghrazi,

Thank you for the submission of your revised Research Article "Bridging energy and proteome allocation models allows prediction of the resource cost of traits across environments" for publication in PLOS Biology. I'm handling your paper termporarily while my colleague Dr Ankiit Ahluwalia is out of the office. On behalf of my colleagues and the Academic Editor, Claudia Bank, I'm pleased to say that we can in principle accept your manuscript for publication, provided you address any remaining formatting and reporting issues. These will be detailed in an email you should receive within 2-3 business days from our colleagues in the journal operations team; no action is required from you until then. Please note that we will not be able to formally accept your manuscript and schedule it for publication until you have completed any requested changes.

Please take a minute to log into Editorial Manager at http://www.editorialmanager.com/pbiology/, click the "Update My Information" link at the top of the page, and update your user information to ensure an efficient production process.

PRESS: We frequently collaborate with press offices. If your institution or institutions have a press office, please notify them about your upcoming paper at this point, to enable them to help maximise its impact. If the press office is planning to promote your findings, we would be grateful if they could coordinate with biologypress@plos.org. If you have previously opted in to the early version process, we ask that you notify us immediately of any press plans so that we may opt out on your behalf.

We also ask that you take this opportunity to read our Embargo Policy regarding the discussion, promotion and media coverage of work that is yet to be published by PLOS. As your manuscript is not yet published, it is bound by the conditions of our Embargo Policy. Please be aware that this policy is in place both to ensure that any press coverage of your article is fully substantiated and to provide a direct link between such coverage and the published work. For full details of our Embargo Policy, please visit http://www.plos.org/about/media-inquiries/embargo-policy/.

Thank you again for choosing PLOS Biology for publication and supporting Open Access publishing. We look forward to publishing your study.

Sincerely,

Roli Roberts

Roland G Roberts, PhD

Senior Editor

PLOS Biology

rroberts@plos.org

on behalf of

Ankiit Ahluwalia, PhD

Associate Editor

PLOS Biology

aahluwalia@plos.org
